# Changes in vascular structure and function in primary hyperparathyroidism: a systematic review and meta-analysis

**DOI:** 10.3389/fendo.2026.1896180

**Published:** 2026-08-11

**Authors:** Ana Gheorghe-Milea, Carmen Emanuela Georgescu

**Affiliations:** 1 5^th^ Department of Medical Sciences, Department of Endocrinology, Iuliu Haţieganu University of Medicine and Pharmacy, Cluj-Napoca, Romania; 2 Endocrinology Clinic, Cluj County Emergency Clinical Hospital, Cluj-Napoca, Romania

**Keywords:** cardiovascular disease, carotid intima-media thickness, endothelial dysfunction, flow-mediated dilation, nitroglycerin-mediated dilation, primary hyperparathyroidism

## Abstract

**Introduction:**

Cardiovascular (CV) diseases, particularly ischemic heart disease and stroke, remain the leading cause of mortality worldwide and constitute a major global health challenge. Primary hyperparathyroidism (PHPT), a prevalent endocrine disorder, has recently emerged as a potential contributor to CV risk, although its impact on vascular structure and function has yet to be fully elucidated.

**Aim:**

This systematic review and meta-analysis aimed to provide an in-depth characterization of vascular involvement by assessing markers of vascular remodeling and function in PHPT, including carotid intima-media thickness (IMT), carotid plaque prevalence, brachial artery flow-mediated dilation (FMD) and nitroglycerin-mediated dilation (NMD), as well as the effects of parathyroidectomy (PTX) on these parameters.

**Methods:**

A comprehensive literature search was conducted across four databases (PubMed, Embase, Web of Science and Scopus) until March 2026. Original studies written in English enrolling patients with PHPT and assessing the pre-specified vascular markers using ultrasound evaluation were included. Thirty-two studies were eligible for the qualitative synthesis, of which thirty were included in the meta-analysis.

**Results:**

Compared with controls, PHPT patients exhibited significantly increased carotid IMT and markedly reduced FMD, indicating early structural arterial changes and endothelial dysfunction. No significant differences were observed in carotid plaque prevalence or in NMD, the latter suggesting preserved endothelium-independent vascular function. PTX was associated with a modest short-term reduction in carotid IMT at 6 months, while improvements in endothelial function were inconsistent and did not reach statistical significance.

**Conclusion:**

Overall, these findings support a model in which PHPT primarily affects vascular function through endothelial impairment, followed by mild structural remodeling, rather than advanced atherosclerotic plaque formation. These alterations may represent an early and potentially reversible stage of vascular disease. Although current guidelines do not include CV involvement as an indication for surgery, vascular markers may contribute to improved risk stratification and management decisions in PHPT. Further prospective studies employing standardized vascular assessment methodologies are needed to clarify the clinical significance of these findings.

## 1 Introduction

Cardiovascular (CV) diseases, particularly ischemic heart disease and stroke, remain the leading cause of mortality worldwide and constitute a major global health challenge (1). Primary hyperparathyroidism (PHPT) is a relatively common endocrine disorder, affecting women more frequently than men, with prevalence estimates of 233 and 85 cases per 100, 000, respectively (2). The observed increase in its detection over recent decades is largely attributable to the routine use of serum calcium measurements (3).

The presence of parathyroid hormone (PTH) receptors in vascular and cardiac tissues suggests that PTH may exert direct CV effects beyond its role in the mineral metabolism (4). PHPT was found to be associated with increased total and CV mortality, as well as a higher risk of cerebrovascular and cardiac events (5–7). Moreover, some studies have reported an increased prevalence of traditional CV risk factors in patients with PHPT, including hypertension, obesity, dyslipidemia, and insulin resistance (5, 8, 9), along with structural alterations of the heart (10). Even within the high-normal range, PTH levels have been associated with a higher risk of CV disease and greater atherosclerotic burden (11–13). Although several studies have reported beneficial effects of parathyroidectomy (PTX) on clinical outcomes related to CV disease (14–16), inconsistent findings across the literature might explain why CV involvement is not currently included among the indications for surgery (17).

A better understanding of the vascular phenotype of PHPT may help elucidate the mechanisms underlying its CV risk profile and improve risk stratification and treatment strategies. Most CV events result from atherosclerosis, a progressive process characterized by accumulation of lipid-rich and fibrous material within the arterial intima. As plaques enlarge, they protrude into the lumen, restricting blood flow and causing tissue ischemia (18). Arterial stiffness, commonly assessed by pulse wave velocity, shows a bidirectional relationship with atherosclerosis: increased stiffness promotes atherosclerotic progression, which in turn further exacerbates arterial wall stiffening (19). A recent meta-analysis including patients with mild PHPT reported increased arterial stiffness in this population (20). However, vascular health is multidimensional, and several ultrasound (US)-derived indices might offer important insights into earlier stages of vascular involvement: carotid intima-media thickness (IMT) and carotid plaque reflect structural atherosclerotic remodeling, whereas flow-mediated dilation (FMD) and nitroglycerin-mediated dilation (NMD) assess endothelium-dependent and endothelium-independent vascular function, respectively. Endothelial dysfunction is regarded as an early step in the atherosclerotic process because it promotes the focal entry, retention, and modification of circulating lipoproteins in the subendothelial compartment, while also causing destabilization of established plaques, therefore increasing the risk of CV events (21, 22). Moreover, together with medial layer remodeling, it contributes to arterial stiffness. FMD of the brachial artery evaluates endothelial function by measuring vasodilation in response to increased blood flow and shear stress, with lower values indicating impairment (23). Decreased brachial artery FMD was found to be closely correlated with the angiographic extent of coronary artery disease (24). NMD evaluates vascular smooth muscle (VSM) responsiveness to externally supplied nitric oxide (NO). Reduced smooth muscle reactivity to NO has been linked to a higher risk of CV events (25). Carotid IMT refers to the combined thickness of the intima and media layers of the carotid artery and is widely used as a surrogate marker of early atherosclerotic changes and subclinical vascular damage (26).

Individual observational studies evaluating these markers in patients with PHPT have yielded heterogeneous and sometimes conflicting results, likely reflecting small sample sizes, differences in disease severity and CV risk burden, as well as variability in US assessment methodology. To provide a more comprehensive characterization of vascular involvement, we conducted a systematic review and meta-analysis to evaluate the impact of PHPT and, when available, PTX on vascular remodeling and function.

## 2 Methods

A systematic review of literature was conducted until March 2026, interrogating four electronic databases (PubMed, Embase, Web of Science and Scopus). The search strategy included all possible combinations between one set of terms related to PHPT (“primary hyperparathyroidism”, “PHPT”, “parathyroid adenoma”, “parathyroid hormone excess”, “parathyroid neoplasm”, “mild primary hyperparathyroidism”, “asymptomatic primary hyperparathyroidism”, “normocalcemic primary hyperparathyroidism”, “parathyroidectomy”) and another set related to the assessment of vascular function and structure (“vascular function”, “vascular dysfunction”, “arterial function”, “arterial dysfunction”, “macrovascular function”, “macrovascular reactivity”, “endothelium”, “endothelial”, “vasorelaxation”, “vascular reactivity”, “vascular responsiveness”, “vasodilatory response”, “vasodilation”, “brachial artery reactivity”, “endothelium function”, “endothelial function”, “endothelial dysfunction”, “endothelium dependent dilation”, “endothelium independent dilation”, “endothelium-dependent vasodilation”, “endothelium dependent vasodilation”, “endothelium-independent vasodilation”, “endothelium-independent vasodilation”, “endothelial-dependent flow”, “vascular smooth muscle”, “smooth muscle function”, “blood flow”, “flow mediated dilation”, “flow-mediated dilation”, “flow-mediated dilatation”, “FMD”, “brachial artery”, “nitroglycerin-mediated dilation”, “NMD”, “nitroglycerin test”, “reactive hyperemia”, “nitric oxide”, “nitrate”, “nitrite”, “vascular tone”, “intima media thickness”, “intima-media thickness”, “IMT”, “carotid artery”, “carotid artery intima-media thickness”, “carotid intima-media thickness”, “carotid thickness”, “cIMT”, “vascular structure”, “vascular wall thickness”, “vascular remodeling”, “arterial wall thickening”, “arterial morphology”, “arterial wall structure”, “vascular morphology”, “arterial remodeling”, “carotid plaque”, “carotid plaques”, “carotid atheroma”, “carotid atherosclerotic lesion”, “carotid atherosclerosis”, “Doppler ultrasound”, “duplex ultrasound”, “vascular ultrasound”, “B-mode ultrasound”, “carotid ultrasound”, “vascular imaging”).

We included original studies written in English enrolling patients with PHPT and assessing vascular function and/or structure. To ensure methodological and clinical homogeneity and enable quantitative synthesis, only measurements obtained through US examination were considered, and outcomes were restricted to prespecified markers of arterial function (brachial artery FMD and NMD) and remodeling (carotid IMT and carotid plaque prevalence). In the quantitative analysis, comparators included control groups and/or within-participant pre–post comparisons following PTX.

The exclusion criteria were: 1) evaluation of other endocrine pathologies (e.g. secondary or tertiary hyperparathyroidism) or mixed populations where PHPT data cannot be separated from other disorders, 2) studies not evaluating any of the four prespecified parameters, 3) wrong publication type (case reports, editorials, letters, errata, notes, commentaries, reviews, meta-analyses, study protocols, conference abstracts, book chapters), 4) languages other than English, 5) studies performed on animals or *in vitro*, 6) studies using assessment methods other than US and 7) studies performed on a duplicate group of patients with the same outcomes. Additionally, studies were excluded from the meta-analysis if they reported vascular outcomes without extractable quantitative data or did not include a control group or a within-participant pre–post PTX comparison.

This systematic review was reported according to the recommendations of the Preferred Reporting Items for Systematic Reviews and Meta-analyses Protocols (PRISMA) (27). The screening of literature was performed by two researchers (AGM and CEG) independently, taking into consideration the inclusion and exclusion criteria. Risk of bias (ROB) was assessed by the same researchers using the quality assessment tools developed by the National Heart, Lung, and Blood Institute (NHLBI): the observational cohort and cross-sectional tool and the before–after (pre-post) tool. When studies included multiple designs, the corresponding tool was applied to each component. Each domain was rated as “Yes, “ “No, “ “Cannot Determine, “ “Not Reported, “ or “Not Applicable.” Overall study quality was classified as “Good”, “Fair”, or “Poor”, according to NHLBI guidance (28). Studies were rated as “Good” when most key domains were satisfied with low ROB. Studies were rated as “Fair” when some concerns were present but without major threats to validity. Studies were rated as “Poor” when multiple critical domains were unmet or unclear, indicating substantial ROB (particularly when lack of blinded outcome assessment, absence of adjustment for confounding, inadequate follow-up, and/or unclear outcome measurement reliability were present). Disagreements were resolved by discussion.

### 2.1 Statistical analysis

Meta-analyses were performed using JASP (version 0.95.4.0). Given anticipated clinical and methodological diversity among studies, random-effects models (REML) were applied to account for residual between-study variability. Mean differences (MDs) were calculated for carotid IMT, brachial artery FMD and NMD, while odds ratios (ORs) were obtained for carotid plaque prevalence. Results were reported as pooled effect sizes with corresponding 95% confidence intervals (CI), p-values, and forest plots. IMT results were expressed in mm, whereas FMD, NMD, and carotid plaque prevalence were expressed as percentages.

When means and standard deviation (SDs) were not reported, they were estimated from the available summary statistics using established approximation methods (29, 30). If numerical data were presented only graphically, values were extracted by digitizing figures with WebPlotDigitizer (version 5.0; calibrated to the figure axes) (31). For pre–post comparisons, when studies did not report the mean change and corresponding SD, these values were calculated from baseline and follow-up data with the methods described in the Cochrane Handbook (29). A moderate correlation coefficient (r = 0.5) was assumed for the primary analysis. Sensitivity analyses were conducted using alternative values (r = 0.3 and r = 0.7) to evaluate the robustness of the findings. For all analyses, a p-value < 0.05 was considered statistically significant.

Publication bias was assessed using funnel plots and the Egger test when at least 10 studies were included in the meta-analysis. Statistical heterogeneity was categorized as low (25–50%), moderate (50–75%), or high (>75%) based on I² values.

To assess the robustness of the results and identify potential outliers or influential studies, case-wise influence diagnostics (including standardized residuals and Cook’s distance) were examined, and leave-one-out sensitivity analyses were performed to evaluate the impact of individual studies on pooled effect estimates and heterogeneity.

In the carotid IMT analyses, studies were classified according to the carotid segment used for measurement—common carotid artery (CCA) or composite carotid measurements (CCA + carotid bulb + internal carotid artery- ICA)—although subgroup analyses were not feasible for all outcomes because of the limited number of available studies. In one study, the carotid segment and side were not reported (32); therefore, the study was excluded from the primary segment-specific meta-analysis to avoid potential misclassification. To evaluate the robustness of the findings, sensitivity analyses were performed in which the values from this study were alternatively assigned to the CCA IMT group and the composite carotid IMT group. The consistency of pooled estimates across these scenarios was subsequently examined.

Subgroup exploratory analyses and univariable meta-regression were conducted to identify potential sources of heterogeneity in outcomes including at least 10 studies. Multivariable meta-regression was not performed due to the limited number of studies available per analysis. For CCA IMT, subgroup analyses were conducted according to the carotid measurement approach (single-side measurements, bilateral measurements, or unspecified), the PHPT phenotype (hypercalcemic or normocalcemic) and the presence or absence of CV risk factors; the last subgroup analysis was also applied to FMD. Differences between subgroups were evaluated using the test of moderators (*Q_M_*). For studies reporting bilateral carotid IMT measurements, the authors’ reported average was used when available; otherwise, left and right values were combined into a single study-level estimate by averaging the means and estimating the SD according to Cochrane Handbook guidance (assuming r = 0.5) (29). When both mean and maximal IMT values were reported, mean IMT was used for the meta-analysis.

Given the heterogeneity in follow-up duration across pre-post studies, we performed time-stratified meta-analyses distinguishing early (≤6 months) from later (>6 months) follow-up. Sensitivity analyses with all follow-up durations were also performed. When studies reported multiple follow-up assessments within the same follow-up category, the timepoint closest to 6 months was selected for the primary analysis, while alternative timepoints were examined in sensitivity analyses.

The study by Petramala et al. (33) included two control groups (healthy subjects and individuals with essential hypertension); for the purposes of the present meta-analysis, the healthy group was selected to ensure consistency with the control populations of the other included studies. When studies reported PHPT participants stratified into different clinical subsets but used a shared control group, an overall estimate for the entire PHPT cohort, obtained from the study or calculated with recommended formulas (29), was used in the primary analysis so that each study contributed only once. Sensitivity analyses were also conducted by substituting the overall estimate with data from each individual stratum within the study.

## 3 Results

### 3.1 Systematic review

**Figure 1:**
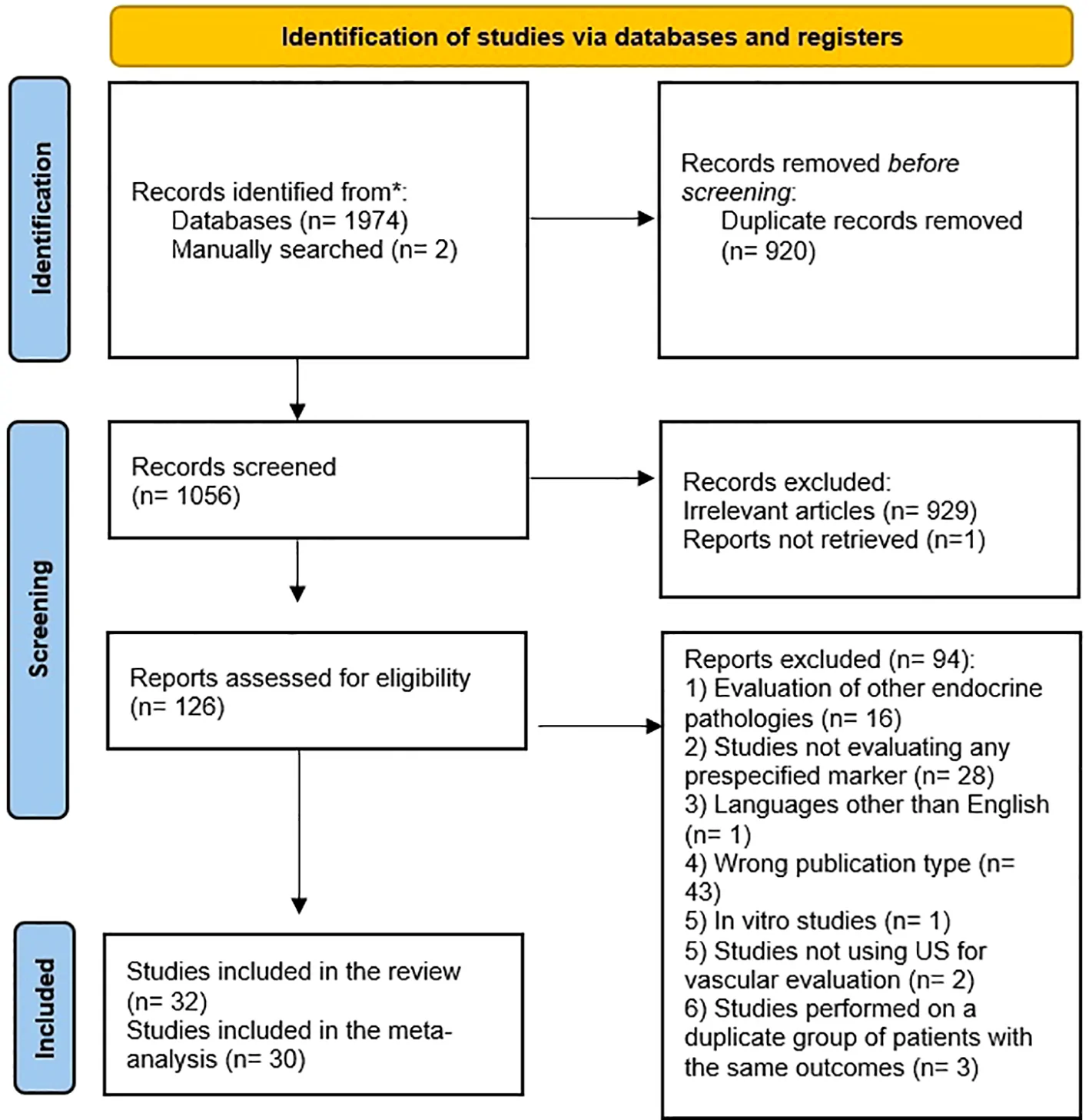
PRISMA diagram of the selection process. US, ultrasound.

Following the electronic database search, 1974 articles were identified. Additionally, two articles were retrieved through manual searching. After exclusion of the duplicates (n = 920), 1056 articles remained. Titles and abstracts were evaluated, and 929 irrelevant articles were excluded, while for one article the full text could not be retrieved (34). The remaining studies were assessed for eligibility: 16 articles were excluded because they evaluated other endocrine disorders, together with 28 studies which did not assess any of the prespecified markers, and two in which vascular assessment methods other than US were employed. Additionally, 43 articles were excluded due to unsuitable publication type, while one study not written in English, one *in vitro* study, and three studies performed on duplicate groups of patients using the same outcomes were also excluded (**Figure 1**).

Thirty-two studies were included in the qualitative analysis, encompassing 1348 patients with PHPT (out of which 474 patients underwent PTX), and 1864 controls. In the study by Walker et al. (35) the authors performed an internal comparison by stratifying PHPT participants according to 25(OH) vitamin D status (<50 nmol/L vs ≥50 nmol/L) and this study was only included in the qualitative analysis as it lacked a control or post-PTX group. The study by Yilmaz et al. (36) was excluded from the quantitative analyses because the reported carotid IMT and brachial artery FMD values were not consistent with the stated measurement units. We attempted to contact the authors for clarification but did not receive a response. Therefore, the meta-analysis included a total of thirty studies, published between 1998 and 2025. Follow-up duration after PTX ranged from 3 to 49 months.

The available data regarding the number of enrolled participants, age, sex, body mass index (BMI), CV risk factors (smoking, diabetes, hypertension, dyslipidemia), established coronary artery disease, systolic blood pressure (SBP), diastolic blood pressure (DBP), total cholesterol, LDL, HDL, triglycerides, PTH or calcium levels and vascular outcomes were extracted from the studies and presented in **Tables 1** and **2**. Data regarding inclusion and exclusion criteria for PHPT and controls, definitions and comments regarding CV risk factors, US assessment methodologies and other vascular outcomes that were evaluated can be found in **Supplementary Table 1**.

**Table 1:** Characteristics of patients enrolled in cross-sectional studies.

First author country (year) [ref]	Characteristics of PHPT patients	Characteristics of control groups	Evaluated parameter	NHLBI rating
Neunteufl Austria (1998) (37)	- n = 26 (69% F); age: 55 ± 15 y; BMI: 25 ± 5 kg/m^2^ - Smokers: 31%; DM: 8%; HT: 23% - Chol: 5.96 ± 1.03 mmol/L - TCa*: 3 ± 0.37 mmol/L; PTH*: 26.4 ± 27.8 pmol/L	- n = 26 (58% F); age: 51 ± 12 y; BMI: 26 ± 3 kg/m^2^ - Smokers: 42%; DM: 12%; HT: 38% - Chol: 5.9 ± 0.8 mmol/L - TCa: 2.32 ± 0.1 mmol/L; PTH: NA	FMD (%): PHPT: 11.6 ± 4.6 Controls: 12.6 ± 4.9 NMD (%)*: PHPT: 11.9 ± 3.9 Controls: 15.6 ± 5.7	Fair
Nilsson Sweden (1999) (38)	- n = 25 (72% F); age: 66 ± 60 y; BMI: 25.8 ± 4.05 kg/m^2^ - Smokers: 20%; DM: 0%; HT: 28%, CAD: 28% - SBP: 130 ± 19.5 mmHg; DBP: 80 ± 9 mmHg; Chol: 5.1 ± 1 mmol/L, TG: 1.6 ± 4 mmol/L - TCa*: 3.01 ± 0.27 mmol/L; PTH*: 15.8 ± 9.5 pmol/L	- n = 25 (72% F); age: 66 ± 55 y; BMI: 26.5 ± 4.05 kg/m^2^ - Smokers: 8%; DM: 8%; HT: 28%; CAD: 24% - SBP: 127 ± 21.79 mmHg; DBP: 77 ± 13.08 mmHg; Chol: 5.7 ± 1.5 mmol/L, TG: 1.5 ± 1 mmol/L - TCa: 2.37 ± 0.1 mmol/L; PTH: 4.6 ± 2.35 pmol/L	Carotid IMT (mm): PHPT: 0.76 ± 0.15 Controls: 0.79 ± 0.25	Fair
Kosch Germany (2000) (39)	- n = 19 (58% F); age: 45 ± 20.5 y; BMI: 24.2 ± 13.95 kg/m^2^ - SBP: 127 ± 21.79 mmHg; DBP: 77 ± 13.08 mmHg; Chol: 5.48 ± 0.87 mmol/L; TG: 1.45 ± 0.92 mmol/L - TCa*: 3 ± 0.35 mmol/L; PTH*: 25.23 ± 24.02 pmol/L	- n = 20 (55% F); age: 46 ± 15.6 y; BMI: 23.6 ± 15.21 kg/m^2^ - SBP: 125 ± 8.94 mmHg; DBP: 76 ± 8.94 mmHg; Chol: 5.56 ± 1.03 mmol/L; TG: 1.37 ± 0.8 mmol/L - TCa: 2.5 ± 0.13 mmol/L; PTH: 2.96 ± 1.39 pmol/L	Carotid IMT (mm): PHPT: 0.64 ± 0.22 Controls: 0.62 ± 0.18 FMD (%)*: PHPT: 4.6 ± 2.62 Controls: 18.4 ± 7.92 NMD (%): PHPT: 22.7 ± 7.85 Controls: 21.8 ± 8.05	Fair
Barletta Italy (2000) (40)	- n = 14 (86% F); age: 60 ± 11 y - SBP: 136 ± 10 mmHg; DBP: 77 ± 7 mmHg - TCa*: 2.88 ± 0.26 mmol/L; ICa*: 1.49 ± 0.1 mmol/L; PTH*: 22.8 ± 19.93 pmol/L	- n = 20 (85% F); age: 60 ± 8 y - SBP: 135 ± 9 mmHg; DBP: 76 ± 5 mmHg - TCa: 2.37 ± 0.18 mmol/L; ICa: 1.2 ± 0.04 mmol/L; PTH: 5.09 ± 0.95 pmol/L	Carotid IMT (mm): PHPT: 0.7 ± 0.1 Controls: 0.7 ± 0.1	Fair
Nuzzo Italy (2002) (41)	- n = 20 (60% F); age: 52.9 ± 9.3 y; BMI: 24.2 ± 1.2 kg/m^2^ - Smokers: 5% - SBP: 135.7 ± 4.6 mmHg; DBP: 82.1 ± 2.4 mmHg - TCa*: 3.04 ± 0.21 mmol/L; ICa*: 1.86 ± 0.7 mmol/L; PTH*: 29.7 ± 17.5 pmol/L	- n = 20 (60% F); age: 54.6 ± 8.6 y; BMI: 23.8 ± 0.9 kg/m^2^ - Smokers: 10% - SBP: 134.3 ± 2.5 mmHg; DBP: 82.1 ± 1.5 mmHg - TCa: 2.27 ± 0.07 mmol/L; ICa: 1.3 ± 0.05 mmol/L; PTH: 4.77 ± 1.27 pmol/L	Left carotid IMT (mm)*: PHPT: 1.5 ± 0.6 Controls: 0.7 ± 0.3 Right carotid IMT (mm)*: PHPT: 1.6 ± 0.5 Controls: 0.68 ± 0.3 Carotid plaque prevalence (%): PHPT: 40 Controls: 10	Fair
Fallo Italy (2003) (42)	*PHPT without CV risk factors (PHPT 1):* - n = 10 (80% F); age: 54 ± 13 y; BMI: 22 ± 3 kg/m^2^ - 0% Smokers, 0% with DM, HT, dyslipidemia or CAD - TCa*: 2.91 ± 0.26 mmol/L; PTH: 20.1 ± 11.1 pmol/L *PHPT with CV risk factors (PHPT 2):* - n = 16 (75% F); age: 58 ± 8 y; BMI*: 28 ± 6 kg/m^2^ - Smokers*: 37.5%; DM*: 25%; HT*: 75%; dyslipidemia*: 62.5%; CAD*: 12.5% - TCa*: 3.05 ± 0.16 mmol/L; PTH: 18.5 ± 14.5 pmol/L	- n = 15 (73% F); age: 59 ± 9 y; BMI: 24 ± 2 kg/m^2^ - 0% Smokers, 0% with DM, HT, Dyslipidemia or CAD - TCa: 2.31 ± 0.22 mmol/L; PTH: NA	Carotid mean IMT (mm): PHPT 1: 0.74 ± 0.1 PHPT 2*: 1.01 ± 0.17 Controls: 0.71 ± 0.14 Carotid maximum IMT (mm): PHPT 1: 0.77 ± 0.1 PHPT 2*: 1.04 ± 0.12 Controls: 0.74 ± 0.12 Carotid plaque prevalence (%): PHPT 1: 0 PHPT 2: 18.75 Controls: 0	Fair
Lumachi Italy (2006) (43)	- n = 27 (81.5% F); age: 61 ± 12 y; BMI: 22.4 ± 4.6 kg/m^2^ - SBP: 136.7 ± 13.9 mmHg; DBP: 83.2 ± 7.1 mmHg; Chol: 3.8 ± 0.8 mmol/L; TG: 1.1 ± 0.5 mmol/L - TCa*: 2.8 ± 0.2 mmol/L; PTH*: 19.4 ± 17.7 pmol/L	- n = 27 (% F: NA); age: 56 ± 14 y; BMI: 23.6 ± 3.8 kg/m^2^ - SBP: 133.5 ± 10.7 mmHg; DBP: 82.7 ± 4.1 mmHg; Chol: 3.9 ± 0.9 mmol/L; TG: 1.7 ± 1.6 mmol/L - TCa: 2.2 ± 0.1 mmol/L; PTH: 5.51 ± 1.27 pmol/L	Carotid IMT (mm): PHPT: 0.86 ± 0.18 Controls: 0.82 ± 0.18	Fair
Baykan Turkey (2007) (44)	- n = 21 (42.8% F); age: 50 ± 11 y; BMI: 28 ± 2.3 kg/m^2^ - SBP: 123 ± 3 mmHg; DBP: 75 ± 2 mmHg; Chol: 5.09 ± 1.16 mmol/L; LDL: 3.6 ± 0.8 mmol/L; HDL: 1.16 ± 0.33 mmol/L; TG: 1.65 ± 1.11 mmol/L - TCa*: 2.89 ± 0.17 mmol/L; PTH*: 51.85 ± 52.49 pmol/L	- n = 27 (48.1% F); age: 49 ± 10 y; BMI: 27 ± 2.7 kg/m^2^ - SBP: 126 ± 4 mmHg; DBP: 74 ± 3 mmHg; Chol: 4.93 ± 1.26 mmol/L; LDL: 3.56 ± 0.75 mmol/L; HDL: 1.18 ± 0.28 mmol/L; TG: 1.64 ± 0.91 mmol/L - TCa: 2.34 ± 0.12 mmol/L; PTH: 2.96 ± 0.9 pmol/L	FMD (%)*: PHPT: 10.2 ± 5.8 Controls: 19.8 ± 5.8	Fair
Walker USA (2009) (45)	- n = 49 (67.3% F*); age: 61.6 ± 7.4 y; BMI*: 25.6 ± 4.1 kg/m^2^ - Smokers: 55%; DM*: 2%; HT: 35% - SBP*: 127 ± 19 mmHg; DBP*: 75 ± 11 mmHg; Chol: 5.45 ± 0.87 mmol/L; LDL: 3.2 ± 0.77 mmol/L; TG*: 1.1 ± 0.63 mmol/L - TCa*: 2.61 ± 0.12 mmol/L; PTH: 17.07 ± 5.4 pmol/L	- n = 991 (95.7% F); age: 63.6 ± 6 y; BMI: 28.3 ± 4.9 kg/m^2^ - Smokers: 51%; DM: 15%; HT: 46% - SBP: 142 ± 21 mmHg; DBP: 85 ± 11 mmHg; Chol: 5.25 ± 1 mmol/L; LDL: 3.38 ± 1 mmol/L; TG: 1.62 ± 0.94 mmol/L - TCa: 2.27 ± 0.09 mmol/L; PTH: NA	Carotid IMT (mm)*: PHPT: 0.96 ± 0.39 Controls: 0.91 ± 0.09 Carotid plaque prevalence (%): PHPT: 39 Controls: 61	Fair
Ekmekci Turkey (2009) (46)	- n = 40 (70% F); age: 48.5 ± 11.64 y; BMI: 26.08 ± 2.06 kg/m^2^ - Smokers: 25% - SBP: 123.6 ± 6.59 mmHg; DBP: 78.02 ± 4.41 mmHg; Chol: 4.89 ± 0.94 mmol/L; LDL: 3.06 ± 0.79 mmol/L; HDL: 1.12 ± 0.13 mmol/L; TG: 1.6 ± 0.73 mmol/L - TCa*: 2.85 ± 0.26 mmol/L; PTH*: 38.07 ± 40.23 pmol/L	- n = 43 (60% F); age: 47.13 ± 8.14 y; BMI: 26.69 ± 3.63 kg/m^2^ - Smokers: 30.2% - SBP: 123.09 ± 8.49 mmHg; DBP: 78.51 ± 4.03 mmHg; Chol: 5.04 ± 1.21 mmol/L; LDL: 3.07 ± 0.96 mmol/L; HDL: 1.21 ± 0.3 mmol/L; TG: 1.57 ± 1.1 mmol/L - TCa: 2.33 ± 0.1 mmol/L; PTH: 4.52 ± 1.66 pmol/L	FMD (%)*: PHPT: 8.48 ± 1.78 Controls: 19.49 ± 2.34	Fair
Petramala Italy (2012) (33)	- n = 30 (73.3% F); age: 54 ± 12 y; BMI: 27.4 ± 4.4 kg/m^2^ - SBP*: 144 ± 10.2 mmHg; DBP*: 90.4 ± 10.3 mmHg; Chol*: 5.88 ± 0.68 mmol/L; LDL*: 3.77 ± 0.55 mmol/L; HDL: 1.39 ± 0.16 mmol/L; TG*: 1.52 ± 0.24 mmol/L - TCa*: 2.79 ± 0.3 mmol/L; ICa*: 1.51 ± 0.2 mmol/L; PTH*: 12.93 ± 5.05 pmol/L	*Healthy controls:* - n = 30 (70% F); age: 55 ± 6 y; BMI: 26.1 ± 2.19 kg/m^2^ - SBP: 129.3 ± 4 mmHg; DBP: 78.4 ± 4 mmHg; Chol: 4.96 ± 0.45 mmol/L; LDL: 3.02 ± 0.48 mmol/L; HDL: 1.48 ± 0.19 mmol/L; TG: 1.06 ± 0.18 mmol/L - TCa: 2.34 ± 0.07 mmol/L; ICa: 1.21 ± 0.02 mmol/L PTH: 3.07 ± 0.25 pmol/L *Controls with essential HT:* - n = 30 (70% F); age: 55 ± 5 y; BMI: 27.1 ± 2.3 kg/m^2^ - SBP*: 131 ± 19 mmHg; DBP*: 82 ± 11 mmHg; Chol*: 5.61 ± 1.07 mmol/L; LDL*: 3.45 ± 0.95 mmol/L; HDL: 1.43 ± 0.36 mmol/L; TG*: 1.58 ± 0.38 mmol/L - TCa: 2.44 ± 0.3 mmol/L; ICa: 1.22 ± 0.03 mmol/L PTH: 3.39 ± 0.56 pmol/L	Carotid IMT (mm): PHPT*: 0.8 ± 0.3 Controls with essential HT*: 0.8 ± 0.1 Healthy controls: 0.6 ± 0.07	Good
Ring Sweden (2012) (47)	- n = 48 (72.9% F); age: 54 ± 8.9 y; BMI: 24 ± 3 kg/m^2^ - SBP*: 126.1 ± 15.8 mmHg; DBP: 79.8 ± 8.8 mmHg; Chol: 5.74 ± 0.8 mmol/L; TG: 1 ± 0.54 mmol/L - TCa*: 2.61 ± 0.12 mmol/L; ICa*: 1.46 ± 0.06 mmol/L; PTH*: 12.72 ± 4.24 pmol/L; 25-OH-D*: 40.6 ± 17.1 nmol/L	- n = 49 (72.9% F); age: 54.8 ± 9 y; BMI: 23.5 ± 2.1 kg/m^2^ - SBP: 119.1 ± 12.6 mmHg; DBP: 75.9 ± 7.8 mmHg; Chol: 5.71 ± 1.03 mmol/L; TG: 0.86 ± 0.43 mmol/L - TCa: 2.29 ± 0.08 mmol/L; ICa*: 1.25 ± 0.04 mmol/L; PTH*: 4.66 ± 1.32 pmol/L; 25-OH-D: 64.1 ± 20.6 nmol/L	Carotid IMT (mm): PHPT: 0.69 ± 0.11 Controls: 0.68 ± 0.14	Good
Agarwal India (2013) (48)	*All patients:* - n = 56 (60.1% F); age: 46.5 ± 13.6 y - TCa*: 2.89 ± 0.32 mmol/L; PTH: 35.87 ± 35.1 pmol/L; 25-OH-D*: 50 ± 6.25 nmol/L *Hypertensive PHPT patients:* - n = 21 - TCa*: 2.82 ± 0.27 mmol/L *Normotensive PHPT patients:* - n = 35 - TCa*: 2.86 ± 0.35 mmol/L	- n = 25 (76% F); age: 44 ± 11.2 y - HT: 0% - TCa: 2.17 ± 0.12 mmol/L; PTH: NA; 25-OH-D: NA	FMD (%): All PHPT*: 9 ± 9 Hypertensive PHPT patients: 9 ± 9 Normotensive PHPT patients*: 10 ± 9 Controls: 12 ± 6 NMD (%): All PHPT*: 20 ± 18 Hypertensive PHPT patients: 19 ± 14 Normotensive PHPT patients*: 21 ± 20 Controls: 24 ± 11	Fair
Asik Turkey (2014) (49)	- n = 38 (89.5% F); age: 57.58 ± 12.22 y; BMI: 28.08 ± 4.34 kg/m^2^ - Smokers: 2.6%; DM*: 15.8%; HT*: 57.9% - SBP*: 138.03 ± 22.89 mmHg; DBP*: 80.29 ± 13.81 mmHg; Chol: 5.07 ± 1.37 mmol/L; LDL: 3.19 ± 1.03 mmol/L; HDL: 1.23 ± 0.35 mmol/L; TG: 3.48 ± 1.68 mmol/L - TCa*: 3.02 ± 0.23 mmol/L; PTH*: 37.62 ± 39.90 pmol/L	- n = 40 (77.5% F); age: 56.48 ± 9.13 y; BMI: 28.5 ± 4.78 kg/m^2^ - Smokers: 5%; DM: 0%; HT: 12.5% - SBP: 120.88 ± 14.41 mmHg; DBP: 74.13 ± 9.33 mmHg; Chol: 4.93 ± 1 mmol/L; LDL: 3.18 ± 0.59 mmol/L; HDL: 1.21 ± 0.28 mmol/L; TG: 4.06 ± 1.79 mmol/L - TCa: 2.31 ± 0.12 mmol/L; PTH: 3.53 ± 1.15 pmol/L	Carotid IMT (mm)*: PHPT: 0.82 ± 0.2 Controls: 0.63 ± 0.11	Fair
Stamatelopoulos Greece (2014) (50)	- n = 102 (100% F); age: 60.6 ± 7.6 y; BMI*: 27.4 ± 4.5 kg/m^2^ - Smokers: 20.6%; HT: 51% - SBP*: 129.8 ± 23.4 mmHg; DBP*: 79.3 ± 13.6 mmHg; Chol: 5.43 ± 0.86 mmol/L; LDL: 3.43 ± 0.83 mmol/L; HDL: 1.61 ± 0.37 mmol/L; TG: 2.67 ± 1.16 mmol/L - TCa*: 2.69 ± 0.16 mmol/L; PTH*: 14.86 ± 6.86 pmol/L; 25-OH-D*: 43.5 ± 22.61 nmol/L	- n = 102 (100% F); age: 60.5 ± 7.5 y; BMI: 25.5 ± 3.5 kg/m^2^ - Smokers: 26.5%; HT: 40.2% - SBP: 120.8 ± 18.4 mmHg; DBP: 73.4 ± 9.4 mmHg; Chol: 5.65 ± 0.88 mmol/L; LDL: 3.52 ± 0.85 mmol/L; HDL: 1.65 ± 0.36 mmol/L; TG: 3.48 ± 1.68 mmol/L - TCa: 2.25 ± 0.86 mmol/L; PTH: 4.27 ± 1.3 pmol/L; 25-OH-D: 59.5 ± 31.13 nmol/L	Carotid IMT (mm): PHPT: 0.69 ± 0.07 Controls: 0.74 ± 0.15 FMD (%): PHPT: 4.7 ± 3.5 Controls: 4.4 ± 4.3 Carotid plaque prevalence (%): PHPT: 25.7 Controls: 20.8	Good
Walker USA (2014) (35)	*Levels of 25-OH-D < 50 nmol/L:* - n = 10 (70% F); age: 59.5 ± 4.7 y; BMI: 25.7 ± 4.9 kg/m^2^ - Smokers: 10%; DM: 10%; HT: 40%; hypercholesterolemia: 40% - SBP: 126 ± 12 mmHg; DBP: 78 ± 9 mmHg - TCa: 2.76 ± 0.22 mmol/L; PTH**: 17.7 ± 10.4 pmol/L; 25-OH-D**: 36 ± 10.25 nmol/L *Levels of 25-OH-D ≥ 50 nmol/L:* - n = 100 (86% F); age: 62.6 ± 9.1 y; BMI: 28.7 ± 7.4 kg/m^2^ - Smokers: 2%; DM: 3%; HT: 38%; hypercholesterolemia: 47% - SBP: 125 ± 17 mmHg; DBP: 75 ± 11 mmHg - TCa: 2.61 ± 0.15 mmol/L; PTH: 8.69 ± 4.24 pmol/L; 25-OH-D: 91 ± 25 nmol/L	- no control group	Carotid IMT (mm): PHPT with levels of 25-OH-D < 50 nmol/L: 0.94 ± 0.11 PHPT with levels of25-OH-D ≥ 50 nmol/L: 0.95 ± 0.11 Carotid plaque prevalence (%): PHPT with levels of 25-OH-D < 50 nmol/L: 60 PHPT with levels of 25-OH-D ≥ 50 nmol/L: 43	Good
Tuna Turkey (2015) (51)	*All patients:* - n = 53 (84.9% F); age: 52.3 ± 3.1 y; BMI: 29.7 ± 6.1 kg/m^2^ - HT*: 34% - LDL: 3.47 ± 0.97 mmol/L; HDL*: 1.41 ± 0.27 mmol/L; TG: 1.6 ± 0.65 mmol/L - TCa*: 2.69 ± 0.17 mmol/L; PTH*: 22.11 ± 9.67 pmol/L; 25-OH-D: 29.7 ± 23.75 nmol/L *Surgically treated:* - n = 35; age: 54 ± 13 y; BMI: 28.4 ± 6.2 kg/m^2^ - TCa: 2.65 ± 0.15 mmol/L; PTH: 23.2 ± 29.4 pmol/L; 25-OH-D: 28.5 ± 24 nmol/L *Observation group:* - n = 18; age: 50 ± 10 y; BMI: 33.2 ± 4.4 kg/m^2^ - TCa: 2.57 ± 0.13 mmol/L; PTH: 16.3 ± 8.5 pmol/L; 25-OH-D: 29.5 ± 24.25 nmol/L	- n = 46 (82.6% F); age: 46.4 ± 9.5 y; BMI: 29.1 ± 5.2 kg/m^2^ - HT: 11% - LDL: 3.25 ± 0.97 mmol/L; HDL: 1.46 ± 0.31 mmol/L; TG: 1.6 ± 0.79 mmol/L - TCa: 2.35 ± 0.07 mmol/L; PTH: 5.45 ± 1.48 pmol/L; 25-OH-D: 37.75 ± 27.5 nmol/L	Carotid IMT (mm)*: All PHPT patients: 0.69 ± 0.18 Surgically treated: 0.65 ± 0.18 Observation group: 0.69 ± 0.12 Controls: 0.61 ± 0.12 FMD (%)*: All PHPT patients: 2.67 ± 1.2 Surgically treated: 3.67 ± 1.2 Observation group: 2.8 ± 9.6 Controls: 14.8 ± 9.6	Fair
Yilmaz Turkey (2015) (36)	- n = 34 (85.2% F); age: 53.58 ± 9.08 y; BMI: 28.33 ± 4.62 kg/m^2^ - Smokers: 20%; HT: 17.6% - SBP*: 130 ± 15 mmHg; DBP*: 78.25 ± 10.75 mmHg; Chol: 4.81 ± 2.38 mmol/L; LDL: 3.02 ± 0.7 mmol/L; HDL: 1.24 ± 0.29 mmol/L; TG: 1.73 ± 0.86 mmol/L - TCa*: 2.72 ± 0.14 mmol/L; PTH*: 15.09 ± 5.23 pmol/L	- n = 29 (79.3% F); age: 56.67 ± 7.56 y; BMI: 29.13 ± 3.99 kg/m^2^ - Smokers: 31%; HT: 10.3% - SBP*: 122.5 ± 12.5 mmHg; DBP*: 77.5 ± 7.5 mmHg; Chol: 5.11 ± 0.71 mmol/L; LDL: 5.11 ± 0.71 mmol/L; HDL: 1.25 ± 0.27 mmol/L; TG: 1.83 ± 0.95 mmol/L - TCa: 2.35 ± 0.07 mmol/L; PTH: 5.71 ± 1.43 pmol/L	Carotid IMT (mm): PHPT: 52 ± 8.5 Controls: 55 ± 8 FMD (%)*: PHPT: 0.1 ± 0.06 Controls: 0.14 ± 0.05	Good
Cansu Turkey (2016) (52)	*Hypercalcemic PHPT group:* - n = 17 (% F: NA); age: 51 ± 8 y; BMI: 28.1 ± 3.7 kg/m^2^ - SBP*: 129 ± 15 mmHg; DBP*: 83 ± 9 mmHg; Chol: 5.53 ± 0.7 mmol/L; LDL: 3.42 ± 0.62 mmol/L; HDL: 1.4 ± 0.26 mmol/L; TG: 1.45 ± 0.45 mmol/L - TCa*: 2.73 ± 0.17 mmol/L; PTH*: 16.11 ± 9.01 pmol/L; 25-OH-D: 66 ± 14 nmol/L *Normocalcemic PHPT group:* - n = 16 (% F: NA); age: 58 ± 7 y; BMI: 28.7 ± 4.6 kg/m2 - SBP*: 131 ± 13 mmHg; DBP*: 81 ± 8 mmHg; Chol: 5.26 ± 0.96 mmol/L; LDL: 3.03 ± 0.75 mmol/L; HDL: 1.5 ± 0.68 mmol/L; TG: 1.5 ± 0.68 mmol/L - TCa: 2.3 ± 0.1 mmol/L; PTH*: 9.65 ± 2.75 pmol/L; 25-OH-D*: 83 ± 22 nmol/L	- n = 15 (100% F); age: 53 ± 4 y; BMI: 27.5 ± 3.9 kg/m^2^ - SBP: 117 ± 14 mmHg; DBP: 73 ± 12 mmHg; Chol: 5.54 ± 0.47 mmol/L; LDL: 3.47 ± 0.44 mmol/L; HDL: 1.48 ± 0.69 mmol/L; TG: 1.48 ± 0.69 mmol/L - TCa: 2.27 ± 0.07 mmol/L; PTH: 4.98 ± 1.27 pmol/L; 25-OH-D: 67 ± 9 nmol/L	Carotid IMT (mm): Hypercalcemic PHPT*: 0.6 ± 0.09 Normocalcemic PHPT*: 0.59 ± 0.07 Controls: 0.52 ± 0.09	Good
Yorulmaz Turkey (2017) (53)	- n = 20; age: 52.7 ± 12.3 y - SBP*: 118 ± 12.8 mmHg; DBP*: 78 ± 7.6 mmHg - TCa*: 2.81 ± 0.12 mmol/L; PTH*: 30.66 ± 8.13 pmol/L	- n = 12; age: NA; - SBP: 104 ± 11.6 mmHg; DBP: 68.3 ± 7.1 mmHg - TCa: 2.34 ± 0.09 mmol/L; PTH: 5.08 ± 0.68 pmol/L	Carotid IMT (mm): PHPT: 0.56 ± 0.09 Controls: 0.48 ± 0.06 FMD (%): PHPT: 8.7 ± 2 Controls: 15.3 ± 2.2 NMD (%): PHPT: 11.9 ± 2.6 Controls: 10.8 ± 3.1	Fair
Colak Turkey (2017) (54)	*Medically treated PHPT group:* - n = 29 (86% F); age: 58.6 ± 9.8 y; BMI: 28 ± 2.5 kg/m^2^ - Smokers: 17.2%; DM: 30%; HT: 41.3% - TCa*: 2.6 ± 0.05 mmol/L; PTH*: 11.65 ± 3.15 pmol/L; 25-OH-D: 56 ± 25 nmol/L *Surgically treated PHPT group:* - n = 25 (72% F); age: 55.7 ± 12.2 y; BMI: 27 ± 3.5 kg/m^2^ - Smokers: 24%; DM: 36%; HT: 40% - TCa*: 2.77 ± 0.1 mmol/L; PTH*: 30.93 ± 18.58 pmol/L; 25-OH-D: 47.25 ± 26.25 nmol/L *Post-PTX group:* - n = 23 (82.6% F); age: 53.4 ± 13.3 y; BMI: 26.5 ± 2.5 kg/m^2^ - Smokers: 17.4%; DM: 8.7%; HT: 43.5% - TCa: 2.3 ± 0.05 mmol/L; PTH: 6.74 ± 2.75 pmol/L; 25-OH-D: 54.75 ± 21.25 nmol/L	- n = 26 (80.7% F); age: 56.9 ± 14.6 y; BMI: 27.25 ± 2.75 kg/m^2^ - Smokers: 17.3%; DM: 27%; HT: 46% - TCa: 2.35 ± 0.07 mmol/L; PTH: 4.32 ± 1.88 pmol/L; 25-OH-D: 97.25 ± 56.25 nmol/L	Carotid IMT (mm): Medically treated PHPT: 0.71 ± 0.12 Surgically treated PHPT: 0.75 ± 0.2 Post-PTX group: 0.69 ± 0.14 Controls: 0.67 ± 0.1 FMD (%)*: Medically treated PHPT: 5.52 ± 2.63 Surgically treated PHPT: 6.02 ± 3.28 Post-PTX group: 7.9 ± 3.05 Controls: 8.62 ± 3.48	Poor
Sambul Turkey (2018) (55)	*Medically treated PHPT group:* - n = 34 (94% F); age: 57.9 ± 7.1 y - SBP: 122.4 ± 12.4 mmHg; DBP: 76.5 ± 10.2 mmHg; Chol: 5.22 ± 0.93 mmol/L; LDL: 3.41 ± 0.59 mmol/L; HDL: 1.25 ± 0.28 mmol/L; TG: 1.93 ± 0.52 mmol/L - TCa*: 2.67 ± 0.14 mmol/L; PTH*: 18.9 ± 10.92 pmol/L; 25-OH-D: 42.25 ± 16.5 nmol/L *Surgically treated PHPT group:* - n = 31 (77.4% F); age: 56.8 ± 15.2 y - SBP: 126.5 ± 16.6 mmHg; DBP: 78.7 ± 12.1 mmHg; Chol: 5.01 ± 1.39 mmol/L; LDL: 3.18 ± 0.82 mmol/L; HDL: 1.23 ± 0.52 mmol/L; TG: 1.77 ± 0.43 mmol/L - TCa*: 2.84 ± 0.22 mmol/L; PTH*: 38 ± 38.26 pmol/L; 25-OH-D: 44 ± 52.7 nmol/L	- n = 30 (66.6% F); age: 56.9 ± 9.8 y - SBP: 121.9 ± 14.9 mmHg; DBP: 75.1 ± 9.2 mmHg; Chol: 5.19 ± 0.82 mmol/L; LDL: 3.23 ± 0.36 mmol/L; HDL: 1.18 ± 0.26 mmol/L; TG: 1.96 ± 0.6 mmol/L - TCa: 2.29 ± 0.08 mmol/L; PTH: 5.53 ± 1.33 pmol/L; 25-OH-D: 46.25 ± 12.75 nmol/L	Common carotid IMT (mm): Medically treated PHPT: 0.75 ± 0.14 Surgically treated PHPT: 0.81 ± 0.15 Controls: 0.74 ± 0.08 Internal carotid IMT (mm): Medically treated PHPT: 0.68 ± 0.17 Surgically treated PHPT: 0.71 ± 0.16 Controls: 0.64 ± 0.09	Fair
Kizilgül Turkey (2018) (32)	- n = 75 (87% F); age: 52.7 ± 10.9 y; BMI: 30.6 ± 5.12 kg/m^2^ - SBP*: 136.56 ± 15.1 mmHg; DBP*: 83.81 ± 7.46 mmHg; LDL: 3.2 ± 0.83 mmol/L; HDL: 1.33 ± 0.31 mmol/L; TG: 1.66 ± 0.69 mmol/L - TCa*: 2.77 ± 0.2 mmol/L; PTH*: 25.12 ± 23.75 pmol/L; 25-OH-D: 36.9 ± 31.7 nmol/L	- n = 96 (76% F); age: 53.3 ± 7.7 y; BMI: 29.5 ± 4.3 kg/m^2^ - SBP: 121.72 ± 10.57 mmHg; DBP: 78.76 ± 5.56 mmHg; LDL: 3.08 ± 0.62 mmol/L; HDL: 1.34 ± 0.3 mmol/L; TG: 1.6 ± 0.78 mmol/L - TCa: 2.33 ± 0.09 mmol/L; PTH: 6.42 ± 2.69 pmol/L; 25-OH-D: 38.27 ± 29.4 nmol/L	Carotid IMT (mm)*: PHPT: 0.67 ± 0.13 Controls: 0.6 ± 0.1	Fair
Kocabaş Turkey (2021) (56)	- n = 44 (88.6% F); age: 49.95 ± 10.55 y; BMI: 30.62 ± 5.58 kg/m^2^ - SBP: 124.77 ± 20.28 mmHg; DBP: 80.45 ± 13.92 mmHg; LDL*: 3.3 ± 0.89 mmol/L; HDL: 1.35 ± 0.5 mmol/L; TG: 2.02 ± 1.03 mmol/L - TCa*: 2.8 ± 0.18 mmol/L; PTH*: 49.07 ± 34.54 pmol/L; 25-OH-D: 35.87 ± 20.95 nmol/L	- n = 40 (90% F); age: 46.73 ± 9.62 y; BMI: 29.6 ± 6.44 kg/m^2^ - SBP: 116.25 ± 19 mmHg; DBP: 76.62 ± 12.3 mmHg; LDL: 2.87 ± 0.94 mmol/L; HDL: 1.37 ± 0.36 mmol/L; TG: 1.58 ± 1.05 mmol/L - TCa: 2.36 ± 0.08 mmol/L; PTH: 3.57 ± 0.45 pmol/L; 25-OH-D: 45.75 ± 37.42 nmol/L	Carotid IMT (mm)*: PHPT: 0.58 ± 0.1 Controls: 0.49 ± 0.1	Fair
Elbuken Turkey (2022) (57)	- n = 37 (86% F); age: 51.2 ± 8 y; BMI*: 31.1 ± 5.4 kg/m^2^ - SBP: 128 ± 14 mmHg; DBP: 82 ± 9 mmHg; Chol: 5.5 ± 0.95 mmol/L; LDL: 3.38 ± 0.82 mmol/L; HDL: 1.39 ± 0.33 mmol/L; TG: 3.77 ± 1.68 mmol/L - TCa*: 2.42 ± 0.07 mmol/L; PTH*: 8.05 ± 3.69 pmol/L; 25-OH-D: 87.75 ± 14.75 nmol/L	- n = 40 (77.5% F); age: 49.3 ± 7.5 y; BMI: 28.5 ± 5.8 kg/m^2^ - SBP: 128 ± 21 mmHg; DBP: 82 ± 13 mmHg; Chol: 5.48 ± 1.06 mmol/L LDL: 3.25 ± 0.85 mmol/L; HDL: 1.42 ± 0.36 mmol/L; TG: 3.93 ± 1.96 mmol/L - TCa: 2.34 ± 0.07 mmol/L; PTH: 5.04 ± 3.11 pmol/L; 25-OH-D: 84 ± 15.25 nmol/L	Carotid IMT (mm)*: PHPT: 0.65 ± 0.1 Controls: 0.59 ± 0.11 Carotid plaque prevalence (%): PHPT: 16.2 Controls: 20.8	Good
Naciu Italy (2025) (58)	- n = 40 (88% F); age: 56.7 ± 8.2 y; BMI: 25.3 ± 5.93 kg/m^2^ - Smokers: 13%; DM: 3%; HT: 33%; dyslipidemia: 8% - SBP: 123 ± 20 mmHg; DBP: 80 ± 11.1 mmHg - TCa*: 2.69 ± 0.12 mmol/L; PTH*: 15.91 ± 10.75 pmol/L; 25-OH-D: 62.5 ± 22.97 nmol/L	- n = 40 (83% F); age: 52.5 ± 10.6 y; BMI: 24.8 ± 3.26 kg/m^2^ - Smokers: 15%; DM: 0%; HT: 20%; dyslipidemia: 8% - SBP: 120 ± 7.4 mmHg; DBP: 80 ± 7.4 mmHg - TCa: 2.37 ± 0.07 mmol/L; PTH: 6.96 ± 2.92 pmol/L; 25-OH-D: 71.5 ± 22.05 nmol/L	Carotid IMT (mm)*: PHPT: 1.1 ± 0.22 Controls: 0.9 ± 0.15 FMD (%)*: PHPT: 3.1 ± 2.44 Controls: 8.5 ± 4.15	Good

For the study by Petramala et al., comparisons were performed using the healthy control group. In the study by Colak et al., the post-PTX and pre-PTX groups consisted of different participants.

*Variables for which p < 0.05 in PHPT vs controls;.

**Variables for which p < 0.05 between PHPT patients with levels of 25-OH-vitamin D < or ≥ 50 nmol/L.

Ref, reference; PHPT, primary hyperparathyroidism; FMD, flow-mediated vasodilation; NMD, nitroglycerin-mediated dilation; IMT, intima-media thickness; n, number of participants; F, female; y, years; BMI, body mass index; Chol, total cholesterol; TCa, total serum calcium; PTH, parathyroid hormone; DM, diabetes mellitus; HT, arterial hypertension; SBP, systolic blood pressure; DBP, diastolic blood pressure; TG, triglycerides; NA, not available; CV, cardiovascular; ICa, ionized serum calcium; CAD, coronary artery disease; LDL, LDL cholesterol; HDL, HDL cholesterol; 25-OH-D, 25-hydroxyvitamin D.

**Table 2:** Characteristics of patients enrolled in longitudinal studies.

First author, country (year) [ref]	Baseline characteristics of PHPT patients	Characteristics of PHPT patients after follow-up	Evaluated parameter before and after PTX	NHLBI rating	Mean follow-up duration (months)
Nilsson Sweden (1999) (38)	- n = 25 (72% F); age: 66 ± 60 y; BMI: 25.8 ± 4.05 kg/m^2^ - Smokers: 20%; DM: 0%; HT: 28%, CAD: 28% - SBP: 130 ± 19.5 mmHg; DBP: 80 ± 9 mmHg; Chol: 5.1 ± 1 mmol/L, TG: 1.6 ± 4 mmol/L - TCa*: 3.01 ± 0.27 mmol/L; PTH*: 15.8 ± 9.5 pmol/L	- n = 17 (76% F); age: 68 ± 45.3 y; BMI: 26.9 ± 4.9 kg/m^2^ - Smokers: 23.5%; DM: 0%; HT: 29.4%; CAD: 35.2% - SBP: 127 ± 16.5 mmHg; DBP: 78 ± 11.13 mmHg; Chol: 5.7 ± 1.4 mmol/L, TG: 1.95 ± 0.8 mmol/L - TCa: 2.34 ± 0.1 mmol/L; PTH: 5.3 ± 2.68 pmol/L	Carotid IMT (mm): Before PTX: 0.76 ± 0.15 After PTX: 0.77 ± 0.16	Fair	10
Neunteufl Austria (2000) (59)	- n = 18 (61% F); age: 51.5 ± 12.6 y; BMI: 26.2 ± 1.7 kg/m^2^ - Smokers: 38%; DM: 6%; HT: 27% - Chol: 6.19 ± 1.14 mmol/L - TCa*: 2.95 ± 0.21 mmol/L; PTH*: 25.76 ± 19.72 pmol/L	- n = 10 (% F: NA); age: 55.1 ± 13.3 y; BMI: 26.2 ± 1.4 kg/m^2^ - Smokers: 33%; DM: 6%; HT: 44% - Chol: 6.27 ± 1.32 mmol/L - TCa: 2.37 ± 0.1 mmol/L; PTH: 3.6 ± 2.65 pmol/L	FMD (%): Before PTX: 12.1 ± 3.1 After PTX: 11 ± 5 NMD (%): Before PTX: 12.5 ± 3.1 After PTX: 13.2 ± 6.2	Poor	42
Kosch Germany (2000) (39)	- n = 19 (58% F); age: 45 ± 20.5 y; BMI: 24.2 ± 13.95 kg/m^2^ - SBP: 127 ± 21.79 mmHg; DBP: 77 ± 13.08 mmHg; Chol: 5.48 ± 0.87 mmol/L; TG: 1.45 ± 0.92 mmol/L - TCa*: 3 ± 0.35 mmol/L; PTH*: 25.23 ± 24.02 pmol/L	- n = 19 (58% F); age: NA; BMI: NA - SBP: 125 ± 26.15 mmHg; DBP: 75 ± 17.44 mmHg; Chol: NA; TG: NA - TCa: 2.4 ± 0.26 mmol/L; PTH: 4.34 ± 9.24 pmol/L	Carotid IMT (mm): Before PTX: 0.64 ± 0.22 After PTX: 0.63 ± 0.22 FMD (%): Before PTX: 4.6 ± 2.62 After PTX: 16.2 ± 6.97 NMD (%): Before PTX: 22.7 ± 7.85 After PTX: 21.4 ± 7.41	Fair	6
Barletta Italy (2000) (40)	- n = 10 (100% F) - SBP: 137 ± 10 mmHg; DBP: 77 ± 7 mmHg - TCa*: 2.89 ± 0.36 mmol/L; PTH*: 20.46 ± 3.6 pmol/L	- n = 10 (100% F) - SBP: 138 ± 10 mmHg; DBP: 78 ± 8 mmHg - TCa: 2.3 ± 0.09 mmol/L; PTH: 5.19 ± 1.59 pmol/L	Carotid IMT (mm): Before PTX: 0.7 ± 0.1 After PTX: 0.7 ± 0.1	Poor	6
Lumachi Italy (2006) (43)	- n = 27 (81.5% F); age: 61 ± 12 y; BMI: 22.4 ± 4.6 kg/m^2^ - SBP: 136.7 ± 13.9 mmHg; DBP: 83.2 ± 7.1 mmHg; Chol: 3.8 ± 0.8 mmol/L; TG: 1.1 ± 0.5 mmol/L - TCa*: 2.8 ± 0.2 mmol/L; PTH*: 19.4 ± 17.7 pmol/L	- n = 27	Carotid IMT (mm): Before PTX: 0.86 ± 0.18 After PTX: 0.77 ± 0.24	Fair	20
Ekmekci Turkey (2009) (46)	- n = 40 (70% F); age: 48.5 ± 11.64 y; BMI: 26.08 ± 2.06 kg/m^2^ - Smokers: 25% - SBP: 123.6 ± 6.59 mmHg; DBP: 78.02 ± 4.41 mmHg; Chol: 4.89 ± 0.94 mmol/L; LDL: 3.06 ± 0.79 mmol/L; HDL: 1.12 ± 0.13 mmol/L; TG: 1.6 ± 0.73 mmol/L - TCa*: 2.85 ± 0.26 mmol/L; PTH*: 38.07 ± 40.23 pmol/L	- n = 40 (60% F); age: NA; BMI: NA - Smokers: 25% - SBP, DBP, Chol, LDL, HDL, TG: NA - TCa: 2.24 ± 0.17 mmol/L; PTH: 4.31 ± 1.02 pmol/L	FMD (%)*: Before PTX: 8.48 ± 1.78 After PTX: 16.19 ± 2.16	Fair	6
Ring Sweden (2012) (47)	- n = 48 (72.9% F); age*: 54 ± 8.9 y; BMI: 24 ± 3 kg/m^2^ - SBP*: 126.1 ± 15.8 mmHg; DBP*: 79.8 ± 8.8 mmHg; Chol: 5.74 ± 0.8 mmol/L; TG*: 1 ± 0.54 mmol/L - TCa*: 2.61 ± 0.12 mmol/L; PTH*: 12.72 ± 4.24 pmol/L; 25-OH-D*: 40.6 ± 17.1 nmol/L	- n = 48 (72.9% F); age: 55.3 ± 8.8 y; BMI: 24.7 ± 3.3 kg/m^2^ - SBP: 123.3 ± 15.2 mmHg; DBP: 78 ± 8.2 mmHg; Chol: 5.85 ± 0.89 mmol/L; TG: 0.93 ± 0.51 mmol/L - TCa: 2.27 ± 0.08 mmol/L; PTH*: 5.25 ± 1.66 pmol/L; 25-OH-D: 58.2 ± 19.5 nmol/L	Carotid IMT (mm): Before PTX: 0.69 ± 0.11 After PTX: 0.7 ± 0.12	Fair	15
Walker USA (2012) (60)	- n = 44 (80% F); age: 62 ± 8 y; BMI: 26 ± 4 kg/m^2^ - Smokers: 5%; DM: 2%; HT: 34% - SBP: 123 ± 15.26 mmHg; DBP: 74 ± 6.63 mmHg; Chol: 5.72 ± 1.06 mmol/L; LDL: 3.63 ± 0.86 mmol/L; HDL: 1.61 ± 0.33 mmol/L; TG: 2.49 ± 1.39 mmol/L - TCa*: 2.62 ± 0.2 mmol/L; PTH*: 10.28 ± 3.52 pmol/L; 25-OH-D*: 70 ± 26.53 nmol/L	*After 12 mo:* - n = 41 - SBP: 122 ± 15.37 mmHg; DBP: 76 ± 6.4 mmHg; Chol: 5.8 ± 1.02 mmol/L; LDL: 3.65 ± 0.83 mmol/L; HDL: 1.58 ± 0.51 mmol/L; TG: 2.8 ± 1.34 mmol/L - TCa: 2.33 ± 0.19 mmol/L; PTH: 3.6 ± 3.39 pmol/L; 25-OH-D: 95 ± 25.61 nmol/L *After 24 mo:* - n = 38 - SBP: 125 ± 16.03 mmHg; DBP: 78 ± 12.33 mmHg; Chol: 5.91 ± 0.99 mmol/L; LDL: 3.78 ± 0.8 mmol/L; HDL: 1.58 ± 0.49 mmol/L; TG: 2.64 ± 1.29 mmol/L - TCa: 2.33 ± 0.18 mmol/L; PTH: 3.6 ± 3.27 pmol/L; 25-OH-D: 100 ± 30.82 nmol/L	Carotid IMT (mm): Before PTX: 0.96 ± 0.05 12 mo after PTX: 0.97 ± 0.05 24 mo after PTX: 0.95 ± 0.05	Good	12 & 24
Petramala Italy (2012) (33)	- n = 30 (73.3% F); age: 54 ± 12 y; BMI: 27.4 ± 4.4 kg/m^2^ - SBP*: 144 ± 10.2 mmHg; DBP*: 90.4 ± 10.3 mmHg; Chol: 5.88 ± 0.68 mmol/L; LDL: 3.77 ± 0.55 mmol/L; HDL: 1.39 ± 0.16 mmol/L; TG: 1.52 ± 0.24 mmol/L - TCa*: 2.79 ± 0.3 mmol/L; ICa*: 1.51 ± 0.2 mmol/L; PTH*: 12.93 ± 5.05 pmol/L	- n = 30 (73.3% F); age: NA; BMI: NA - SBP: 117 ± 19.2 mmHg; DBP: 83 ± 14.3 mmHg; Chol: 5.4 ± 0.65 mmol/L; LDL: 3.41 ± 0.6 mmol/L; HDL: 1.47 ± 0.31 mmol/L; TG: 1.33 ± 0.39 mmol/L - TCa*: 2.22 ± 0.52 mmol/L; ICa*: 1.2 ± 0.08 mmol/L; PTH*: 5.39 ± 2.33 pmol/L	Carotid IMT (mm): Before PTX: 0.8 ± 0.3 After PTX: 0.7 ± 0.2	Fair	12
Carrelli USA (2013) (61)	- n = 45 (80% F); age: 61 ± 6.71 y; BMI: 25.3 ± 4.02 kg/m^2^ - Smokers: 5%; DM: 2%; HT: 33% - SBP: 123 ± 13.42 mmHg; DBP: 74 ± 6.71 mmHg; Chol: 5.4 ± 0.67 mmol/L - TCa*: 2.65 ± 0.2 mmol/L; PTH*: 10.5 ± 4.7 pmol/L; 25-OH-D*: 70 ± 26.83 nmol/L	*6 mo after PTX:* - n = 45 - SBP: 121 ± 20.12 mmHg; DBP: 74 ± 13.42 mmHg - TCa: 2.33 ± 0.2 mmol/L; PTH: 3.8 ± 2.01 pmol/L; 25-OH-D: 92 ± 33.54 nmol/L *12 mo after PTX:* - n = 45 - SBP: 123 ± 20.12 mmHg; DBP*: 77 ± 6.71 mmHg - TCa: 2.33 ± 0.2 mmol/L; PTH: 3.6 ± 2.01 pmol/L; 25-OH-D: 95 ± 33.54 nmol/L	Brachial artery end-diastolic diameter (mm): Before PTX: 3.4 ± 0.67 6 mo after PTX: 3.43 ± 0.67 12 mo after PTX: 3.43 ± 0.74 FMD (%): Before PTX: 4.63 ± 3.42 6 mo after PTX: 4.38 ± 5.57 12 mo after PTX: 5.07 ± 4.96	Good	6 &12
Agarwal India (2013) (48)	*All patients:* - n = 56 (60.1% F); age: 46.5 ± 13.6 y - TCa*: 2.89 ± 0.32 mmol/L; PTH: 35.87 ± 35.1 pmol/L; 25-OH-D*: 50 ± 6.25 nmol/L *Hypertensive PHPT patients:* - n = 21 - TCa*: 2.82 ± 0.27 mmol/L *Normotensive PHPT patients:* - n = 35 - TCa*: 2.86 ± 0.35 mmol/L	*All patients:* 3 mo after PTX: - n = 45 - TCa: 2.22 ± 0.22 mmol/L 6 mo after PTX: - n = 45 - TCa: 2.27 ± 0.17 mmol/L *Hypertensive PHPT patients:* 3 mo after PTX: - n = 21 - TCa: 2.27 ± 0.27 mmol/L 6 mo after PTX: - n = 21 - TCa: 2.27 ± 0.17 mmol/L *Normotensive PHPT patients:* 3 mo after PTX: - n = 35 - TCa: 2.2 ± 0.17 mmol/L 6 mo after PTX: - n = 35 - TCa: 2.27 ± 0.2 mmol/L	FMD (%): *All patients:* Before PTX: 10 ± 9 3 mo after PTX: 11 ± 9 6 mo after PTX: 13 ± 17 *Hypertensive PHPT patients:* Before PTX: 10 ± 10 3 mo after PTX: 12 ± 8 6 mo after PTX: 11 ± 6 *Normotensive PHPT patients:* Before PTX: 10 ± 9 3 mo after PTX: 10 ± 9 6 mo after PTX: 15 ± 22 NMD (%): *All patients:* Before PTX: 20 ± 17 3 mo after PTX: 18 ± 13 6 mo after PTX: 23 ± 13 *Hypertensive PHPT patients:* Before PTX: 21 ± 16.5 3 mo after PTX: 21 ± 16 6 mo after PTX*: 26 ± 17 *Normotensive PHPT patients:* Before PTX: 21 ± 18 3 mo after PTX: 16 ± 10 6 mo after PTX: 22 ± 9	Fair	3 & 6
Tuna Turkey (2015) (51)	- n = 20 - HT*: 34% - LDL: 3.42 ± 0.86 mmol/L; HDL*: 1.45 ± 0.31 mmol/L - TCa*: 2.71 ± 0.77 mmol/L; PTH*: 37.44 ± 12.93 pmol/L	- n = 20 - HT: 11% - LDL: 3.23 ± 1.09 mmol/L; HDL: 1.5 ± 0.33 mmol/L - TCa: 2.37 ± 0.17 mmol/L; PTH: 9.22 ± 3.39 pmol/L	Carotid IMT (mm)*: Before PTX: 0.7 ± 0.2 After: 0.6 ± 0.09 FMD (%)*: Before PTX: 4 ± 6 After PTX: 16 ± 5.3	Fair	6
Cansu Turkey (2016) (52)	- n = 17; age: 51 ± 8 y; BMI: 28.1 ± 3.7 kg/m^2^ - SBP: 129 ± 15 mmHg; DBP: 83 ± 9 mmHg; Chol*: 5.53 ± 0.7 mmol/L; LDL*: 3.42 ± 0.62 mmol/L; HDL: 1.4 ± 0.26 mmol/L; TG: 1.45 ± 0.45 mmol/L - TCa*: 2.73 ± 0.17 mmol/L; PTH*: 16.11 ± 9.01 pmol/L; 25-OH-D*: 66 ± 14 nmol/L	- n = 17 - SBP: 124 ± 14 mmHg; DBP: 78 ± 9 mmHg; Chol: 5.91 ± 0.85 mmol/L; LDL: 3.73 ± 0.7 mmol/L - TCa: 2.23 ± 0.07 mmol/L; PTH: 5.3 ± 1.16 pmol/L; 25-OH-D: 78 ± 22 nmol/L	Carotid IMT (mm)*: Before PTX: 0.6 ± 0.09 After PTX: 0.54 ± 0.07	Good	6
Karakose Turkey (2016) (62)	- n = 48 (79.2% F); age: 52.1 ± 11.3 y; BMI: 30.1 ± 5.3 kg/m^2^ - Smokers: 14.6%; HT: 47.9% - SBP*: 137.5 ± 13.5 mmHg; DBP*: 84 ± 7.4 mmHg; Chol: 5.16 ± 1.01 mmol/L; LDL: 3.09 ± 0.8 mmol/L; HDL: 1.3 ± 0.33 mmol/L; TG: 1.68 ± 0.73 mmol/L - TCa*: 2.71 ± 0.14 mmol/L; PTH*: 23.46 ± 16.42 pmol/L; 25-OH-D*: 38.5 ± 35.75 nmol/L	- n = 48 (79.2% F); age: 54.4 ± 12 y; BMI: NA - SBP: 129 ± 12.4 mmHg; DBP: 80.1 ± 7.2 mmHg; Chol: 5.3 ± 1 mmol/L; LDL: 3.23 ± 0.8 mmol/L; HDL: 1.28 ± 0.31 mmol/L; TG: 1.7 ± 0.94 mmol/L - TCa: 2.34 ± 0.12 mmol/L; PTH: 6.7 ± 2.57 pmol/L; 25-OH-D: 90.25 ± 45.75 nmol/L	Carotid IMT (mm)*: Before PTX: 0.68 ± 0.12 After PTX: 0.63 ± 0.1	Fair	6
Carnevale Italy (2024) (63)	*Surgical management:* - n = 22 (100% F); age: 63.24 ± 7.72 y; BMI: 27.96 ± 4.61 kg/m^2^ - Smokers: 18.2%; DM: 13.6%; HT: 63%; - SBP*: 125.38 ± 10.2 mmHg; DBP*: 71.86 ± 8.46 mmHg; Chol: 5.11 ± 0.88 mmol/L; LDL: 2.95 ± 0.85 mmol/L; HDL: 1.58 ± 0.42 mmol/L; TG: 1.07 ± 0.41 mmol/L - ICa*: 1.53 ± 0.14 mmol/L; PTH*: 22.48 ± 9.64 pmol/L; 25-OH-D*: 41.57 ± 19.1 nmol/L *Conservative management:* - n = 30 (100% F); age: 61.1 ± 8.14 y; BMI: 27.26 ± 4.53 kg/m^2^ - Smokers: 3.3%; DM: 6.6%; HT: 40% - SBP*: 121.87 ± 11.96 mmHg; DBP*: 74.4 ± 8.92 mmHg; Chol: 5.34 ± 0.83 mmol/L; LDL: 3.32 ± 0.97 mmol/L; HDL: 1.53 ± 0.42 mmol/L; TG: 1.19 ± 0.52 mmol/L - ICa*: 1.39 ± 0.05 mmol/L; PTH*: 12.42 ± 5.34 pmol/L; 25-OH-D: 51.25 ± 22 nmol/L	*Surgical management:* - n = 22 (100% F); age, BMI: NA - SBP: 132.18 ± 16.02 mmHg; DBP: 76.8 ± 11.13 mmHg; Chol: 4.87 ± 1.06 mmol/L; LDL: 2.8 ± 0.93 mmol/L; HDL: 1.55 ± 0.37 mmol/L; TG: 1.06 ± 0.31 mmol/L - ICa: 1.19 ± 0.08 mmol/L; PTH: 4.96 ± 2.32 pmol/L; 25-OH-D: 75.25 ± 36.35 nmol/L *Conservative management:* - n = 30 (100% F); age, BMI: NA - SBP: 131.67 ± 14.16 mmHg; DBP: 79.67 ± 7.65 mmHg; Chol: 5.24 ± 1 mmol/L; LDL: 3.05 ± 0.94 mmol/L; HDL: 1.61 ± 0.42 mmol/L; TG: 1.23 ± 0.55 mmol/L - ICa: 1.4 ± 0.07 mmol/L; PTH: 14.28 ± 5.57 pmol/L; 25-OH-D: 61.37 ± 23.52 nmol/L	*Surgical management:* Carotid IMT (mm): Before PTX: 0.85 ± 0.14 After PTX: 0.89 ± 0.22 Carotid plaque prevalence (%): Before PTX: 40.91 After PTX: 45.45 *Conservative management:* Carotid IMT (mm)*: Baseline: 0.8 ± 0.18 After PTX: 0.93 ± 0.23 Carotid plaque prevalence (%): Baseline: 26.67 After PTX: 36.67	Good	*Surgical management:* 32 *Conservative management*: 28
Çakıl Turkey (2024) (64)	- n = 35 (91.4% F); age: 52.03 ± 9.54 y; BMI: 30.41 ± 4.63 kg/m^2^ - SBP*: 127.29 ± 18.4 mmHg; DBP: 80.43 ± 14.42 mmHg; LDL: 3.32 ± 0.9 mmol/L; HDL: 2.06 ± 1.08 mmol/L; TG: 2.09 ± 0.92 mmol/L - TCa*: 2.92 ± 0.23 mmol/L; PTH*: 28.7 ± 15.4 pmol/L	- n = 35 (91.4% F); age: NA; BMI: 30.46 ± 4.68 kg/m^2^ - SBP: 138.57 ± 19.9 mmHg; DBP*: 85.11 ± 13.06 mmHg; LDL: 3.2 ± 0.8 mmol/L; HDL: 1.45 ± 0.38 mmol/L; TG: 2.48 ± 1.73 mmol/L - TCa: 2.36 ± 0.09 mmol/L; PTH: 5.9 ± 2.69 pmol/L	Carotid IMT (mm)*: Before PTX: 0.7 ± 0.15 After PTX: 0.57 ± 0.13	Fair	18

*Variables for which p < 0.05 in PHPT patients before surgery vs after surgery.

ref, reference; PHPT, primary hyperparathyroidism; PTX, parathyroidectomy; FMD, flow-mediated vasodilation; NMD, nitroglycerin-mediated dilation; IMT, intima-media thickness; n, number of participants; F, female; y, years; BMI, body mass index; Chol, total cholesterol; TCa, total serum calcium; PTH, parathyroid hormone; DM, diabetes mellitus; HT, arterial hypertension; SBP, systolic blood pressure; DBP, diastolic blood pressure; TG, triglycerides; NA, not available; CV, cardiovascular; ICa, ionized serum calcium; CAD, coronary artery disease; LDL, LDL cholesterol; HDL, HDL cholesterol; 25-OH-D, 25-hydroxyvitamin D; mo, months.

Two studies included only postmenopausal women (50, 63). Most investigations included hypercalcemic PHPT patients, with the majority of cohorts exhibiting mild hypercalcemia. One study was limited to normocalcemic PHPT (57) and one stratified patients into two groups (hyper- and normocalcemic PHPT) (52). Additionally, four studies (51, 54, 55, 63) stratified PHPT patients according to the treatment strategy (conservative vs surgical). Across most studies, patients with PHPT and controls were comparable in sex distribution, age, BMI, blood pressure, and lipid profiles. However, Walker et al. (45) reported lower SBP, DBP, triglyceride levels, and diabetes prevalence in the PHPT group. In contrast, several studies observed a higher burden of CV risk factors in the PHPT group: greater prevalence of hypertension and diabetes (49), higher hypertension prevalence and lower HDL levels (51), elevated SBP (47) or both SBP and DBP (32, 33, 50, 52, 53), higher BMI (50, 57), or increased LDL (33), total cholesterol and triglyceride levels (33, 56).

Carotid IMT was generally assessed by high-resolution carotid artery US using linear array transducers with frequencies ranging from 5 to 15 MHz, most studies using B-mode US, while one (52) used RF-based quality IMT assessment. Most studies reported the mean carotid IMT measured at the far wall. Elbuken et al. (57) and Fallo et al. (42) also provided maximum CCA IMT values, while three studies (33, 35, 54) included near-wall assessments. Fifteen studies performed IMT measurements exclusively at the level of the CCA; among these, eight reported bilateral measurements, three reported unilateral measurements (two on the left side and one on the right side), and four did not specify the side of assessment. Composite carotid IMT values were reported in seven studies, all of which performed bilateral assessment. In six of these studies, the authors reported a mean value from both sides, whereas in the study by Nuzzo et al. (41), separate right and left values were provided and were averaged to obtain a bilateral estimate. Studies reporting 6-month follow-up data performed measurements at the level of the CCA (two reported left-sided values, two did not specify the side, and one reported the average of bilateral measurements). Across the included studies, carotid plaque definitions varied considerably, ranging from relative wall thickening (> 50% compared with the adjacent wall) to criteria based on absolute protrusion or thickness thresholds, as well as approaches classifying plaque using IMT cut-offs (e.g. > 1.3 mm). In one study (33), data on carotid plaque prevalence were reported only for the PHPT group and not for the control or post-PTX groups, precluding inclusion in the meta-analysis.

FMD was assessed in 13 studies, using high-resolution brachial artery US, most of them imaging the right brachial artery longitudinally above the antecubital fossa using linear array transducers (5–14 MHz) after several minutes of rest. Two studies did not report the side of measurement (46, 61). Reactive hyperemia was typically induced by forearm or upper-arm cuff inflation to suprasystolic pressures for 4–5 minutes, followed by cuff release and serial post-deflation diameter measurements. Most studies measured peak diameter at approximately 60 seconds after deflation, although some performed repeated measurements to identify the maximal response (37, 39, 50, 54, 59). NMD was assessed in four studies using high-resolution US of the brachial artery under resting conditions. Three studies used the right brachial artery, while one (48) did not specify the side of measurement. After baseline diameter acquisition, endothelium-independent vasodilation was induced by sublingual nitrate administration (nitroglycerin or isosorbide dinitrate). Arterial diameter was then reassessed at predefined time points ranging from serial measurements (e.g., 1, 3, and 5 minutes) to a single post-dose assessment (typically around 5 minutes), with some protocols performing repeated measurements every 30 seconds and defining the peak response as the mean of consecutive maximal values. Across studies, brachial artery NMD and FMD were consistently expressed as the percentage change in brachial artery diameter from baseline.

Several studies examined associations between PTH or calcium levels and vascular markers. Most studies did not observe significant correlations with carotid IMT. The only significant associations were reported by Lumachi et al. (43) for PTH levels and by Asik et al. (49) for serum calcium levels. Regarding FMD, several studies identified significant associations with PTH (46, 51, 53, 54) and with calcium levels (36, 44, 46, 51, 53, 54, 58).

### 3.2 Quality assessment

A total of 26 articles were assessed using the NHLBI quality assessment tool for observational cohort and cross-sectional studies. In addition, 16 articles were assessed using the NHLBI quality assessment tool for before-after (pre-post) studies with no control group. Among the observational cohort and cross-sectional studies, 8 studies were rated as “Good”, 17 studies were rated as “Fair”, and 1 study was rated as “Poor.” (**Table 1**) Among the before-after (pre-post) studies, 4 studies were rated as “Good, “ 10 studies were rated as “Fair, “ and 2 studies were rated as “Poor.” (**Table 2**) Overall, all studies clearly stated their research question or objective. However, several methodological limitations were identified. In the observational cohort and cross-sectional studies, sample size justification or power calculations were rarely reported, and participation rates were often unclear or not reported. Although exposure and outcome measures were generally well defined and consistently assessed across participants, adjustment for key potential confounding variables was not performed in several studies, which might introduce residual confounding. Regarding the before-after studies, most studies clearly described the intervention and outcome measures, and statistical methods assessing pre- to post-intervention changes were reported in all studies. However, sample sizes were generally small, and blinding of outcome evaluators was rarely reported. In addition, several studies had incomplete information regarding participant representativeness or enrollment of all eligible participants.

### 3.3 Meta- analysis

#### 3.3.1 CCA IMT in PHPT patients vs controls

**Figure 2:**
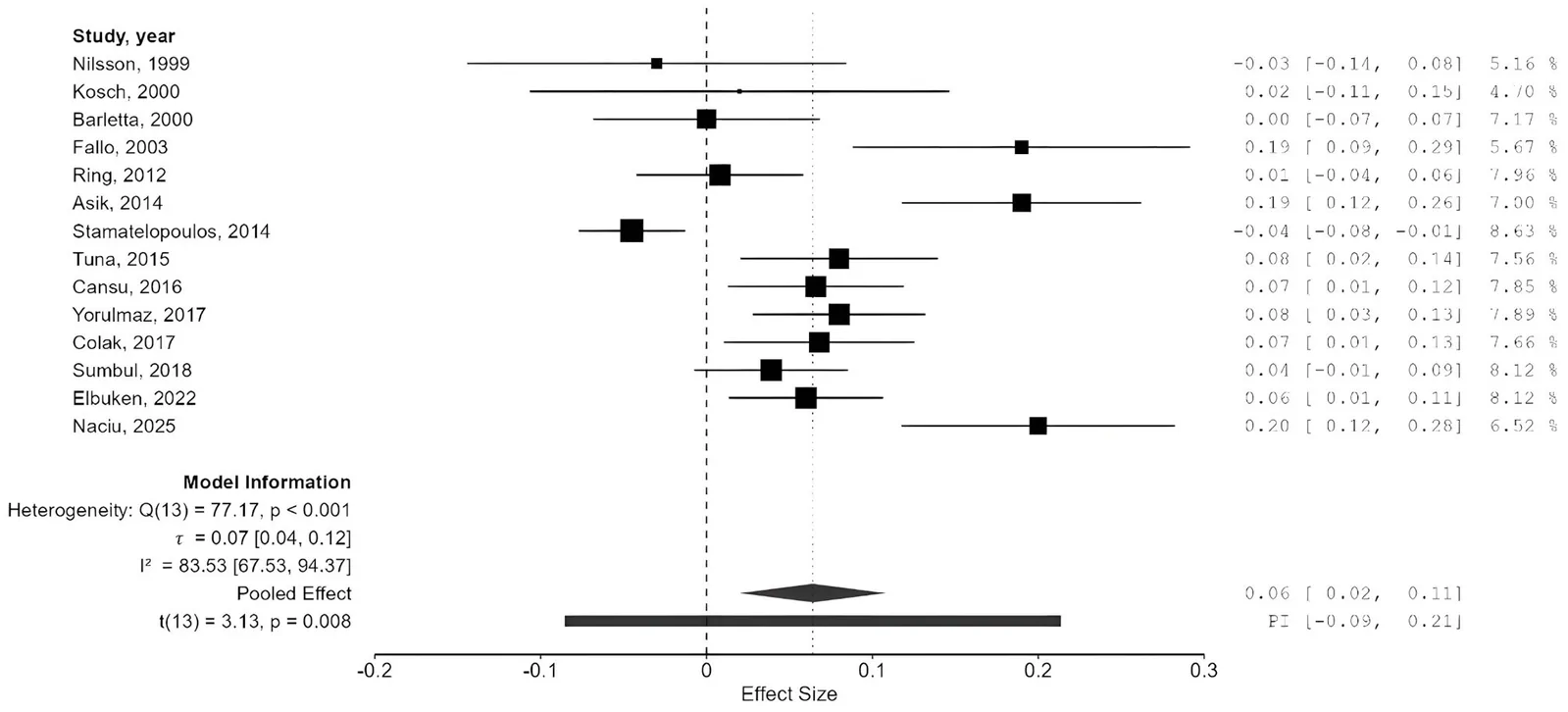
Forest plot of the meta-analysis of common carotid artery intima–media thickness in patients with PHPT versus controls.

In the meta-analysis of 14 studies, including 574 patients with PHPT and 480 controls, CCA IMT was found to be significantly increased in the former group (MD = 0.06 mm, 95% CI: 0.02–0.11, p = 0.008). However, heterogeneity between studies was high (I² = 83.53%, p < 0.001). (**Figure 2**) In contrast with these findings, the study by Yilmaz et al. (36) revealed a slightly higher CCA IMT in controls, but the difference was not statistically significant.

Leave-one-out sensitivity analyses indicated that the pooled MD was not driven by any single study. Excluding individual studies resulted only in minor changes in the pooled estimate (range: 0.04–0.06 mm), the association remained statistically significant in all analyses and heterogeneity remained high. Visual inspection of the funnel plot suggested slight asymmetry (**Supplementary Figure 1**), which was also identified by Egger’s test (p = 0.035).

Subgroup analysis by CV risk factors (**Supplementary Figure 2**) showed increased CCA IMT in PHPT patients, both in studies including patients with such risk factors (MD = 0.10 mm, 95% CI: 0.006–0.19, p = 0.046) and in those without them (MD = 0.04 mm, 95% CI: 0.01–0.07, p = 0.013). Heterogeneity was high in the former studies (I² = 91.58%), but low in the latter studies (I² = 15.81%). However, the test for subgroup differences was not statistically significant (p = 0.162), indicating that the effect of PHPT on CCA IMT did not differ significantly according to the presence of CV risk factors. Similarly, subgroup analysis by CCA IMT measurement laterality (**Supplementary Figure 3**) showed no significant differences between studies using bilateral, unilateral, or unspecified measurements (p = 0.67). Although all showed increased CCA IMT in PHPT patients, the number of studies within each subgroup was small and the effects did not reach statistical significance. In the subgroup analysis according to PHPT phenotype (**Supplementary Figure 4**), CCA IMT was increased in both hypercalcemic PHPT (MD = 0.07 mm, 95% CI 0.02–0.11, p = 0.013) and normocalcemic PHPT (MD = 0.06 mm, 95% CI 0.04–0.08, p = 0.019). However, the latter estimate was derived from only two studies—Elbuken et al. (57) and the normocalcemic stratum reported by Cansu et al. (52)—and should therefore be interpreted with caution. The test for subgroup differences was not statistically significant (p = 0.859), suggesting that the association between PHPT and CCA IMT did not differ according to PHPT phenotype.

Sensitivity analyses using stratum-specific data from studies that categorized PHPT patients by treatment strategy (51, 54, 55), as well as from the study which stratified patients into hyper- and normocalcemic (52), yielded similar estimates with those obtained in the primary analysis.

Meta-regression analyses were conducted to explore potential sources of heterogeneity, including the following variables: age, sex, mean levels of calcium, PTH, total cholesterol, BMI, SBP and DBP. No significant moderators were identified. (**Supplementary Table 2**)

#### 3.3.2 Composite carotid IMT in PHPT patients vs controls

**Figure 3:**
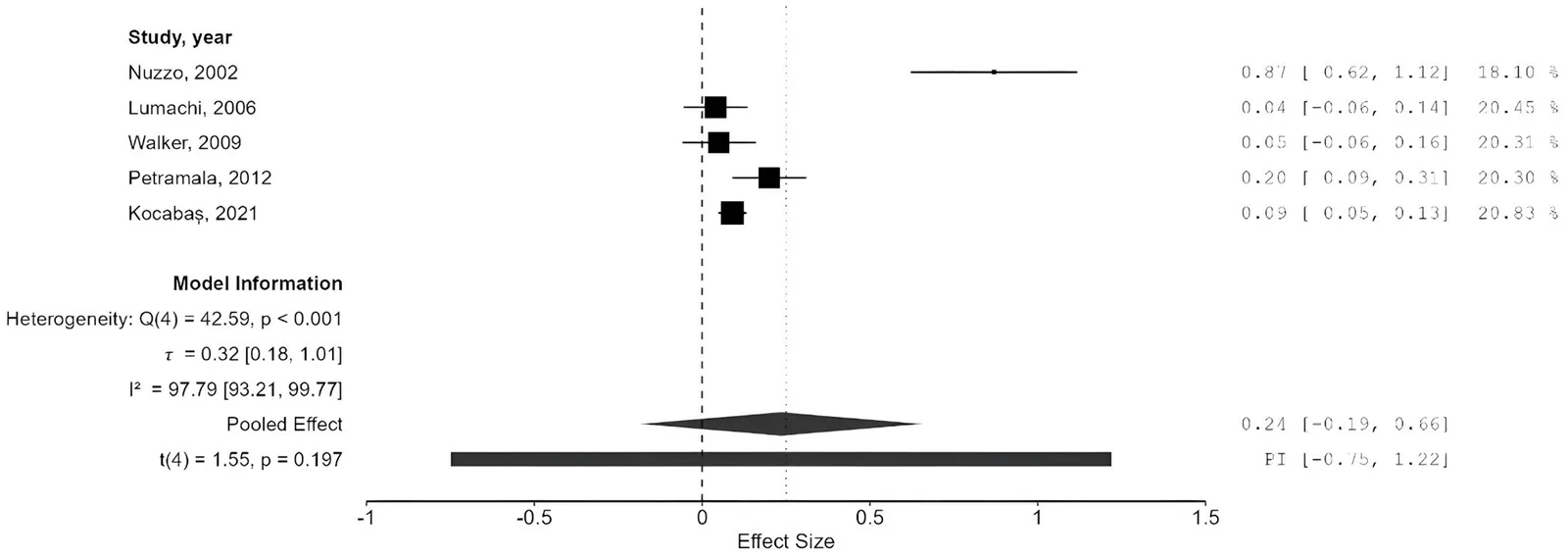
Forest plot of the meta-analysis of composite carotid intima–media thickness in patients with PHPT versus controls.

The pooled analysis of 5 studies including 170 patients with PHPT and 1108 controls, using composite carotid IMT measurements, also showed increased IMT in the former group; however, the difference did not reach statistical significance (MD = 0.24 mm, 95% CI: -0.19 to 0.66, p = 0.197). Heterogeneity was extremely high (I² = 97.79%). (**Figure 3**) One influential study was identified (41) and although its exclusion reduced heterogeneity (I² = 37.36%), the pooled estimate remained non-significant (MD = 0.09 mm, 95% CI: -0.01 to 0.19, p = 0.058). Leave-one-out analyses showed that removing other studies did not materially affect the results.

In the sensitivity analyses in which data from Kizilgül et al. (32) were alternatively included in the CCA IMT and composite carotid IMT groups, the pooled effects (MD = 0.06 mm, p = 0.004 and MD = 0.20 mm, p = 0.165, respectively) and heterogeneities (I^2^ = 83.26%, p < 0.001 and I^2^ = 98.49%, p < 0.001) were not materially changed compared with the primary analyses.

#### 3.3.3 Carotid IMT in PHPT patients pre- vs post-PTX at 6 months

**Figure 4:**
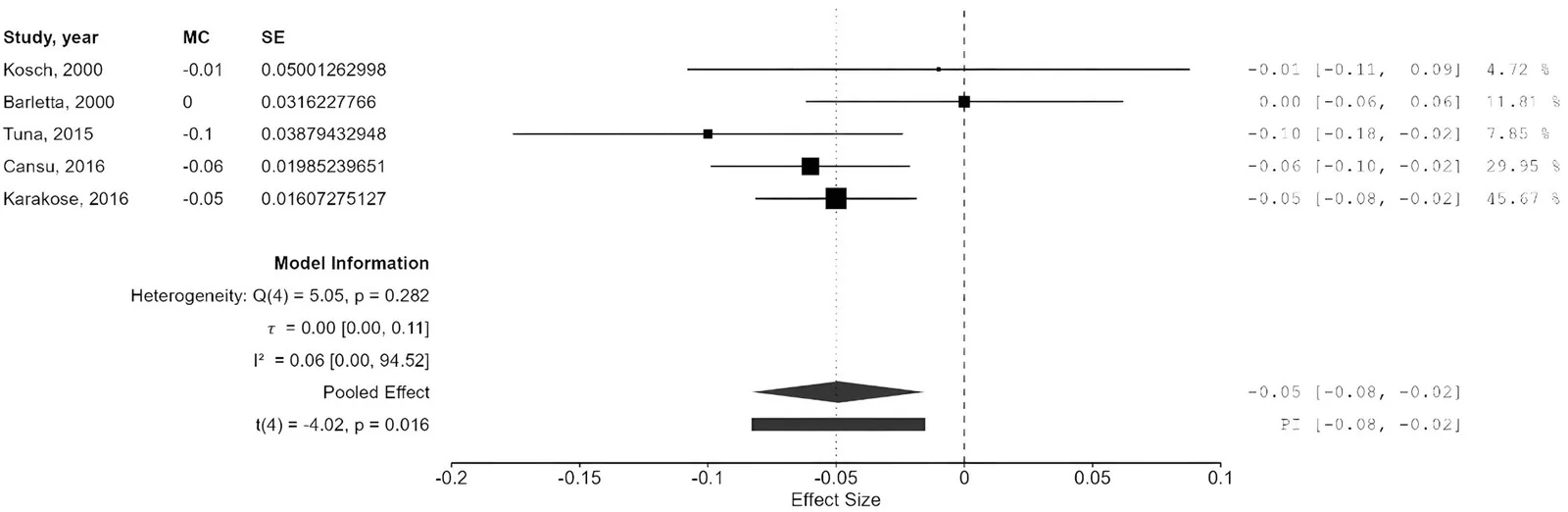
Forest plot of the meta-analysis of carotid intima–media thickness in PHPT patients pre- and post-PTX at 6 months.

Data on carotid IMT measurements obtained 6 months after surgery, all performed exclusively at the level of the CCA, were reported in five studies involving 114 PHPT patients who underwent PTX. This intervention was associated with a significant reduction in carotid IMT (MD= -0.05 mm, 95% CI: -0.08 to -0.02) and heterogeneity was low (I² = 0%). (**Figure 4**) Case-wise influence diagnostics did not identify any potential outliers. However, leave-one-out analyses showed that exclusion of two individual studies (52, 62) rendered the pooled effect non-significant, although the direction of the effect remained consistent.

#### 3.3.4 Carotid IMT in PHPT patients pre- vs post- PTX after > 6 months

**Figure 5:**
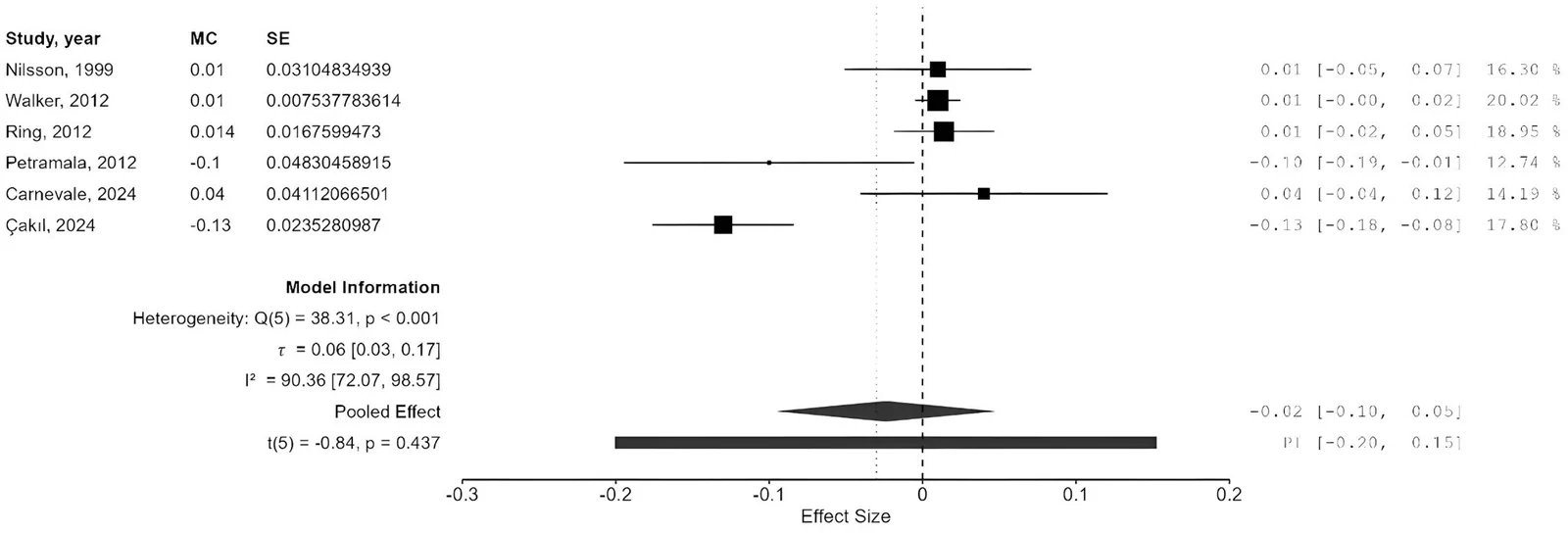
Forest plot of the meta-analysis carotid intima–media thickness in PHPT patients pre- and post-PTX after > 6 months.

Six studies, evaluating 193 PHPT patients who underwent PTX, provided longer follow-up periods after surgery. Four of them evaluated CCA IMT and two assessed composite carotid IMT (33, 60). The pooled effect on carotid IMT was not statistically significant (MD= -0.02 mm, 95% CI: -0.1 to 0.05, p = 0.437), with substantial heterogeneity (I² = 90.36%). (**Figure 5**) Sensitivity analysis including values at 24 months from the study of Walker et al. (60) did not affect the pooled estimate and heterogeneity.

One study was identified as influential (64) and leave-one-out analysis demonstrated that exclusion of this study reversed the direction of the pooled effect (MD = 0.01 mm, 95% CI: -0.05 to 0.07, p = 0.719) and reduced I² to 0%.

In both short- and long-term follow-ups, sensitivity analyses using different assumed pre–post correlations (r = 0.3 and 0.7) did not materially alter the results. When all studies were pooled irrespective of follow-up duration, the reduction in carotid IMT did not reach statistical significance (MC = -0.04 mm, 95% CI: -0.08 to 0; p = 0.059) and heterogeneity was substantial (I² = 77.48%).

#### 3.3.5 Carotid plaque prevalence in PHPT patients vs controls

**Figure 6:**
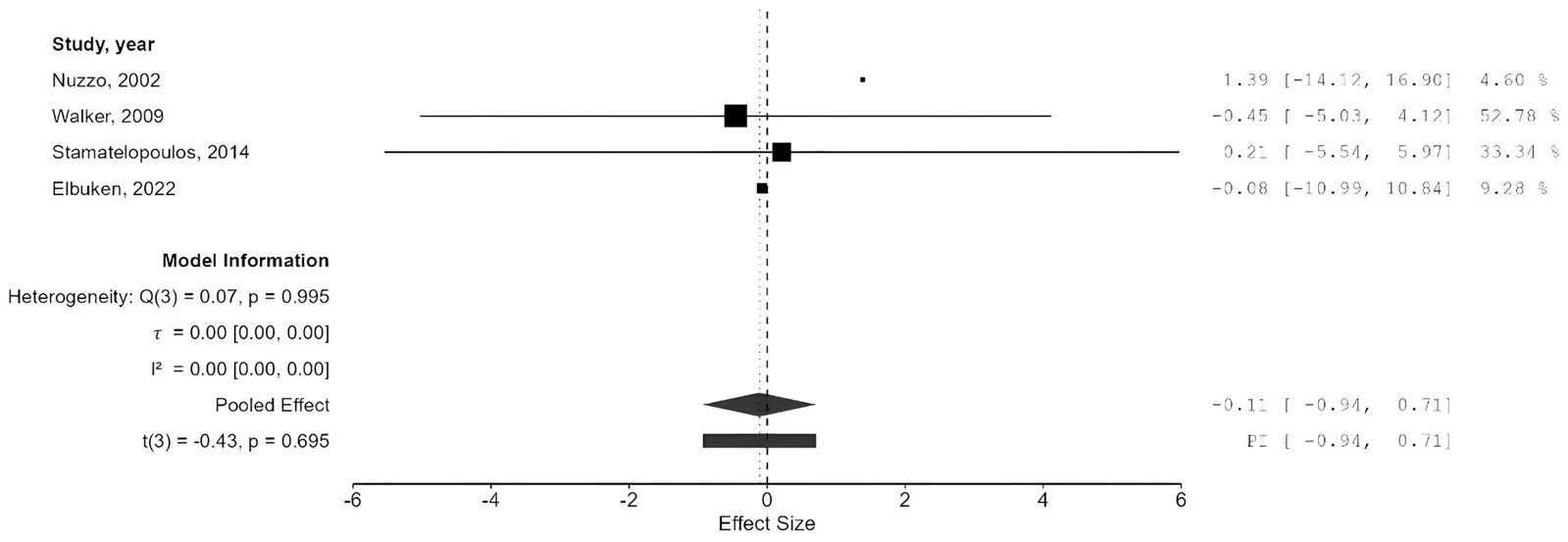
Forest plot of the meta-analysis of carotid plaque prevalence in patients with PHPT versus controls.

The meta-analysis of four studies assessing carotid plaque prevalence in subjects with PHPT (208 patients) and controls (1153 individuals) showed no significant difference between groups (OR = 0.90, 95% CI: 0.39–2.03, p = 0.695). Heterogeneity was negligible (I² = 0%). (**Figure 6**) One study (45) was identified as influential and leave-one-out analysis indicated that removal of this study resulted in a change in the direction of the pooled effect estimate (OR = 1.31, 95% CI: 0.41–4.23, p = 0.423). Exclusion of any other individual study did not affect the magnitude, direction, or statistical significance of the pooled estimate.

#### 3.3.6 FMD in PHPT patients vs controls

**Figure 7:**
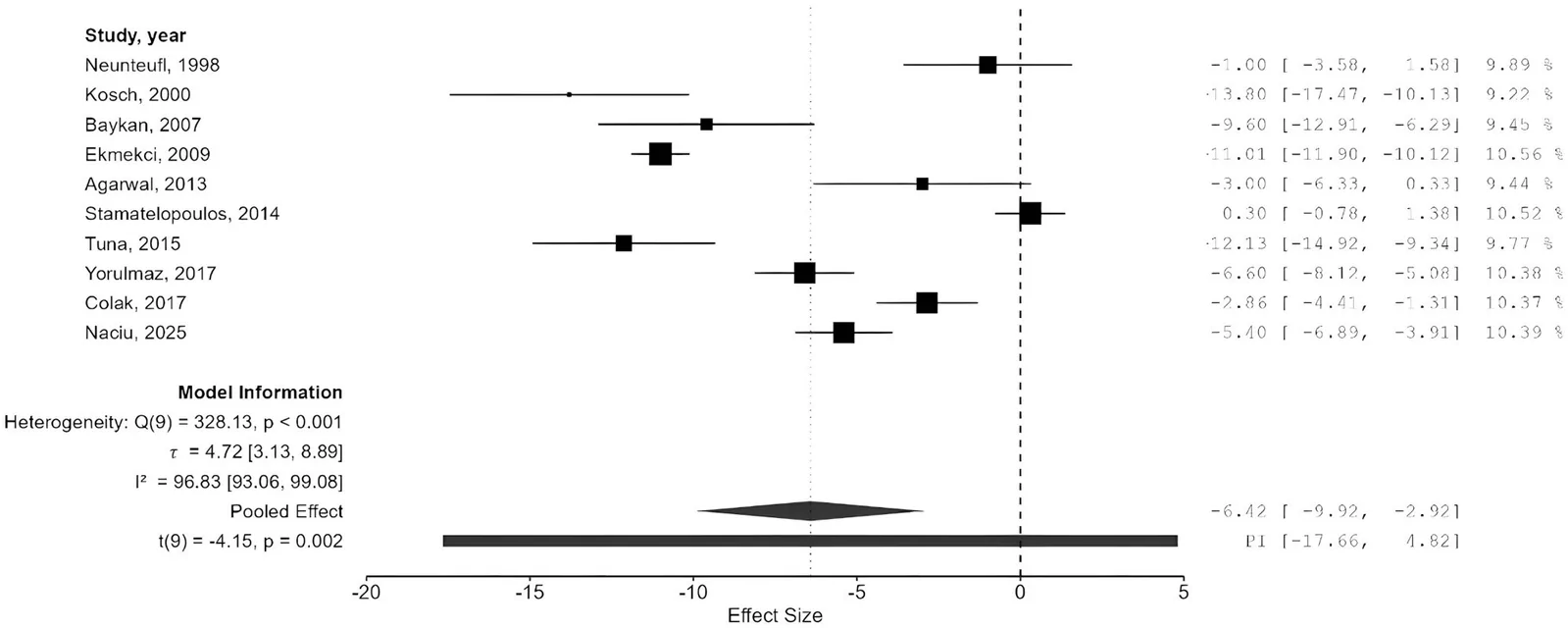
Forest plot of the meta-analysis of flow-mediated dilation in patients with PHPT versus controls.

Across 10 studies, including 431 patients with PHPT and 367 controls, FMD was significantly lower in the former group (MD = -6.42%, 95% CI: -9.92 to -2.92, p = 0.002); however, heterogeneity was high (I² = 96.8%). (**Figure 7**) Case-wise influence diagnostics did not identify any potential outliers. Leave-one-out analyses did not result in changes in the pooled effect and heterogeneity remained high across models. The study by Yilmaz et al. (36) also revealed statistically significant lower values of FMD in PHPT patients compared to controls.

Subgroup analysis by CV risk factors (**Supplementary Figure 5**) showed significantly lower FMD in PHPT patients compared with controls, both in studies including patients with such risk factors (MD = -5.01%, 95% CI: -9.48 to 0.55, p = 0.033) and in those without them (MD = -9.74%, 95% CI: -18.74 to -0.74, p = 0.043). The test for subgroup differences was not statistically significant (p = 0.089). Sensitivity analyses using stratum-specific data from the studies which divided PHPT patients according to treatment strategy (51, 54) yielding comparable effect estimates with the primary analysis.

Meta-regression analyses were conducted to explore potential sources of heterogeneity, including the same variables presented for CCA IMT. Mean age was identified as a significant moderator of the difference in FMD between PHPT patients and controls. (**Supplementary Table 3**)

The funnel plot appeared visually asymmetric, with several studies showing large negative effect sizes (**Supplementary Figure 6**). However, Egger’s test did not indicate significant funnel plot asymmetry (p = 0.997). Given the very high heterogeneity (I² = 96.8%) and the small number of included studies (k = 10), these results should be interpreted with caution.

#### 3.3.7 FMD in PHPT patients pre- vs post- PTX at 6 months

**Figure 8:**
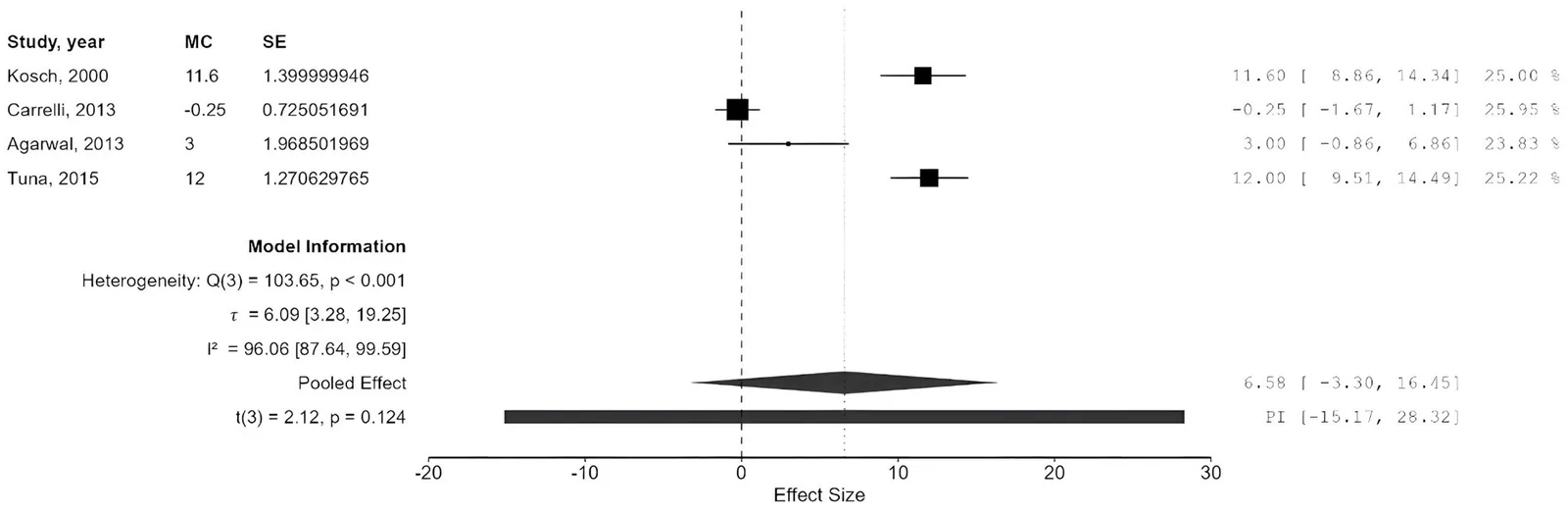
Forest plot of the meta-analysis of flow-mediated dilation in PHPT patients pre- and post -PTX at 6 months.

The meta-analysis of studies assessing FMD before and 6 months after PTX, including 129 patients, demonstrated an increase in FMD, but the result was not statistically significant (MD = 6.58%, 95% CI: -3.3 to 16.5, p = 0.124). Between-study heterogeneity was high (I² = 96%) (**Figure 8**).

Sensitivity analyses using different assumed pre–post correlations (r = 0.3 and 0.7) did not materially alter the results. Leave-one-out analysis showed that exclusion of individual studies did not alter the direction or statistical significance of the pooled effect and high heterogeneity persisted (I² > 90%). Using 3-month values instead of 6-month values from the study by Agarwal et al. (48) did not affect the pooled estimate or heterogeneity, nor did substituting the overall PHPT group data with that of the normotensive (MD = 7.1%, 95% CI: -2.4 to 16.6; I² = 95%) or hypertensive groups (MD = 6.0%, 95% CI: -4.5 to 16.6; I² = 97%) from the same study.

#### 3.3.8 FMD in PHPT patients pre- vs post-PTX after > 6 months

**Figure 9:**
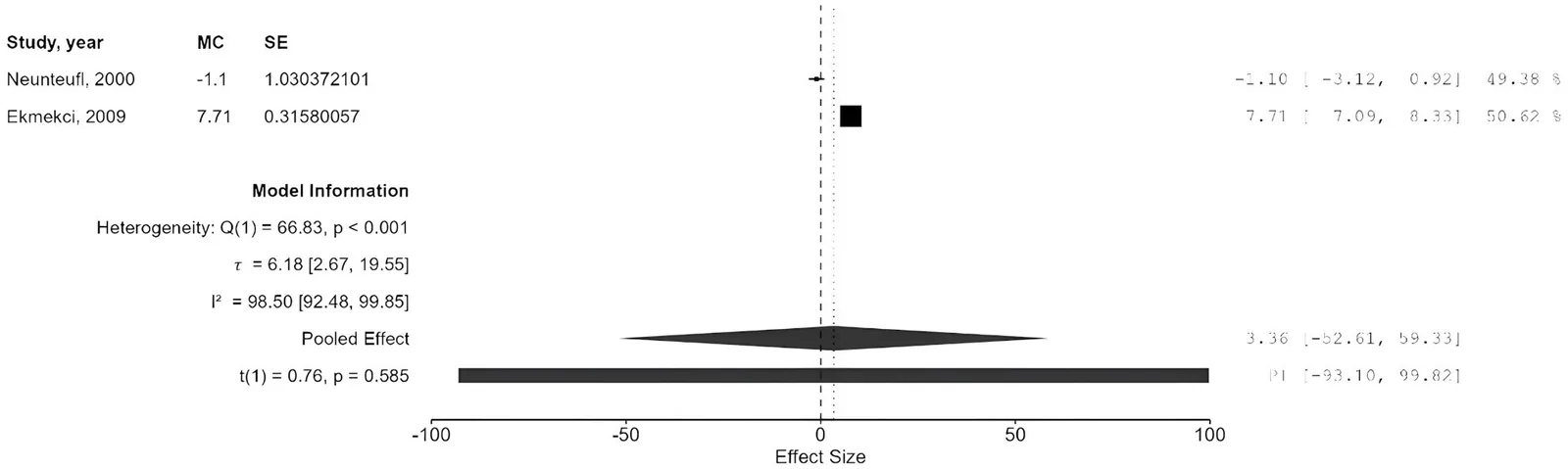
Forest plot of the meta-analysis of flow-mediated dilation in PHPT patients pre- and post-PTX after > 6 months.

Two studies, including 50 PHPT patients, provided longer postoperative follow-ups. The pooled effect on FMD was not statistically significant (MD = 3.36%, 95% CI: -52.61 to 59.33, p = 0.585) and showed high heterogeneity (I² = 98.5%) (**Figure 9**).

In the sensitivity analysis including all available follow-up durations, PTX was associated with a trend towards improvement in FMD (MC = 5.48%, 95% CI: -0.60 to 11.56). However, the result was not statistically significant (p = 0.068) and heterogeneity was considerable (I² ≈ 98%). Exclusion of one study (59) resulted in a stronger and statistically significant pooled effect (MD = 6.81%, 95% CI: 0.14 to 11.56, p = 0.047). Sensitivity analyses using different assumed pre–post correlations (r = 0.3 and 0.7) yielded similar results.

#### 3.3.9 NMD in PHPT patients vs controls

**Figure 10:**
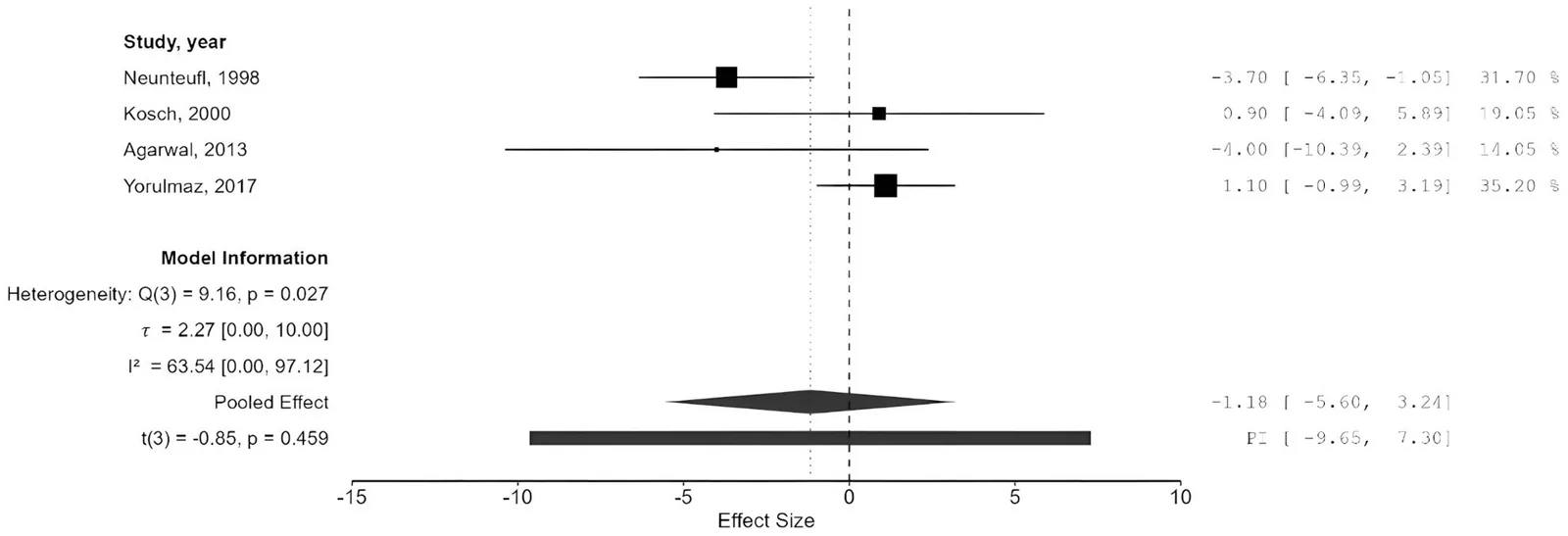
Forest plot of the meta-analysis of nitroglycerin-mediated dilation in patients with PHPT versus controls.

Meta-analysis of the four studies evaluating NMD, including 121 PHPT patients and 83 controls, showed no significant difference between the two groups (MD= -1.18%, 95% CI: -5.60 to 3.24, p = 0.459), with moderate heterogeneity (I² = 63.5%). (**Figure 10**) Case-wise diagnostics identified one study (59) as influential and leave-one-out analysis demonstrated that exclusion of this study reduced heterogeneity from I² = 63.5% to 0%. However, it did not materially alter the overall non-significant pooled effect. Removal of other studies did not meaningfully reduce heterogeneity or change statistical significance.

#### 3.3.10 NMD in PHPT patients pre- vs post- PTX at 6 months

**Figure 11:**
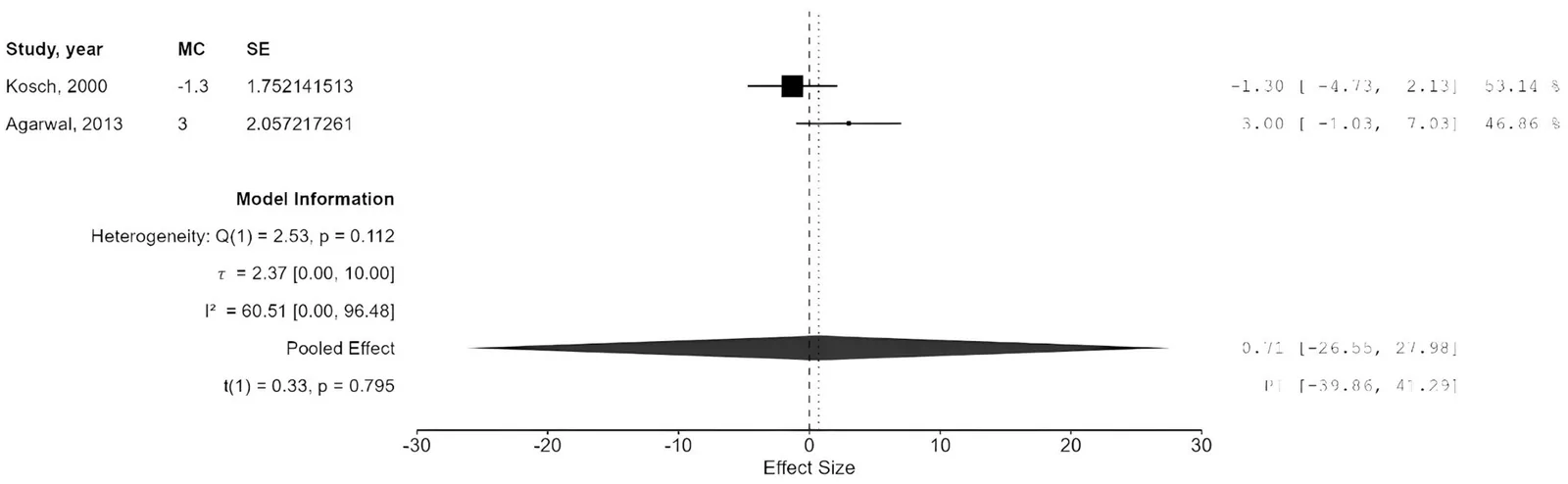
Forest plot of the meta-analysis of nitroglycerin-mediated dilation in PHPT patients pre- and post-PTX at 6 months.

The meta-analysis of the only two available pre–post PTX studies, including 64 PHPT patients, showed no statistically significant change in NMD 6 months after PTX (MD = 0.71%, 95% CI: −26.5 to 27.9, p = 0.795). Between-study heterogeneity appeared moderate (I²= 60.5%) (**Figure 11**).

Sensitivity analyses using different assumed pre–post correlations (r = 0.3 and 0.7) did not materially alter the results, nor did substituting the overall PHPT group data with that of the normotensive (MD = -0.4%, 95% CI: -14.7 to 14.1) or hypertensive groups (MD = 1.7%, 95% CI: -38.3 to 41.7) for the study of Agarwal et al. (48).

## 4 Discussion

The present systematic review and meta-analysis revealed that PHPT patients exhibit lower FMD and increased carotid IMT compared to control subjects. Current evidence does not indicate a clear effect of PHPT on carotid plaque prevalence or NMD. Regarding the impact of PTX on these parameters, surgery was associated with a modest reduction in carotid IMT after 6 months, but no significant improvement in endothelial function.

### 4.1 Structural changes

Carotid IMT is a reproducible, non-invasive measure that correlates with major CV risk factors, established atherosclerotic disease, and future CV events and provides prognostic information beyond traditional risk factors (65–67). Several major guidelines endorse carotid IMT as a tool for detecting subclinical atherosclerosis and refining CV risk assessment in selected intermediate-risk populations (68, 69). Serum calcium concentrations within the normal range have been shown to correlate positively with carotid IMT in overweight and obese populations (70). Similarly, several studies have identified associations between circulating PTH levels and carotid IMT (43, 71–74); however, this relationship has not been consistently observed (75, 76) and most studies included in the present review did not demonstrate such an association.

Even modest changes in carotid IMT have been associated with important changes in CV outcomes. For instance, the risk of myocardial infarction (MI) increased by 11% with each 0.1 mm increase in maximum CCA IMT (77) in one study, while each increase in CCA IMT of 0.16 mm was associated with about 41% higher odds of stroke in another (78). Moreover, a meta-analysis (67) demonstrated that each 0.1 mm increment in carotid IMT resulted in a 10–15% increase in MI risk and a 13–18% increase in stroke risk. Therefore, even modest differences in carotid IMT such as those identified in our meta- analysis may translate into an increased risk of CV events in patients with PHPT.

The decision to analyze CCA IMT and composite carotid IMT separately was supported by accumulating evidence that these metrics capture different aspects of vascular pathology. Because atherosclerotic plaques preferentially develop at the carotid bifurcation and ICA (79), composite carotid IMT measures were proposed as a better marker of overall atherosclerotic burden than single-segment assessments and indeed showed a stronger association with CV events (80). Moreover, another analysis demonstrated that IMT measured at the level of the carotid bulb, as well as plaque burden, improved prediction of incident atherosclerotic events beyond traditional risk factors, whereas CCA IMT alone was less discriminative (81). Consistently, the IMPROVE study reported superior risk stratification with multi-segment composite carotid IMT measures compared with CCA IMT alone (82).

The composite carotid IMT analysis revealed the same trend towards increased values in PHPT patients compared to controls, albeit not statistically significant and with a very high between-study heterogeneity. Exclusion of the study by Nuzzo et al. (41) markedly reduced heterogeneity, possibly reflecting differences in sample size, greater biochemical disease severity, a selected normotensive cohort, and a distinct US protocol targeting maximal wall thickness. Notably, for this study, the mean from left and right measurements was not available and was calculated for the primary analysis.

In the analysis of CCA IMT in PHPT vs controls, the heterogeneity was also considerable. Measurement protocol variability was identified in the studies included in this meta-analysis, in particular concerning differences in the side of measurement, use of far- vs near-wall measurements, transducer frequency and US techniques, and could account for the increased heterogeneity (83–85). Previous evidence showed that the left carotid wall is typically thicker than the right one in individuals aged 35–65 years, with right-sided thickening occurring roughly a decade later, highlighting inherent side-to-side variability (86). Therefore, in order to improve reliability and reproducibility of carotid IMT assessment, bilateral measurements are generally recommended (87). Subgroup analyses showed no significant differences in the effect of PHPT on CCA IMT according to measurement side. Nevertheless, studies using bilateral carotid measurements demonstrated an increase in CCA IMT of borderline statistical significance in patients with PHPT compared with controls (p = 0.05), whereas unilateral measurements showed non-significant effects, potentially reflecting the greater precision achieved when both carotid arteries are evaluated.

Another potential source of heterogeneity, particularly in outcomes with small sample sizes or skewed distributions, might be the inclusion of studies in which means and SDs were estimated from available summary statistics, as these methods assume normality and SD estimates are generally less reliable than mean estimates (88). In addition, other factors such as ethnic background, sex distribution, renal function, disease duration or vitamin D status might have further contributed to between-study heterogeneity. In a population-based cohort of older adults, serum 25(OH) vitamin D levels showed an inverse dose-response relationship with ICA IMT, whereas no such association was observed for 1, 25(OH)_2_ vitamin D or PTH (89). However, other studies found no significant association between 25(OH) vitamin D and carotid IMT or carotid plaque, and vitamin D insufficiency or deficiency did not appear to contribute to the development of early vascular changes in overweight and obese children (73, 75). Accordingly, in the study by Walker et al. (35) carotid IMT and carotid plaque prevalence did not differ significantly between PHPT patients with levels below or above 50 nmol/L.

Irrespective of the carotid segment evaluated (CCA alone or CCA, bulb and/or ICA), findings from both analyses underscore the need for rigorous standardization of carotid IMT acquisition and reporting protocols in PHPT research, to improve comparability, reduce heterogeneity, and clarify the true vascular impact of the disease. Several protocols are available, including the Mannheim consensus, which recommends measuring mean carotid IMT on the far wall of the distal CCA in plaque-free segments, typically within a 10-mm region proximal to the carotid bulb, with bilateral averaging and preference for semi-automated edge detection to enhance reproducibility (90). Another widely used tool is the ARIC protocol, which derives mean carotid IMT from plaque-free far-wall measurements obtained across multiple carotid segments- including the CCA, bifurcation, and ICA- often using multiple interrogation angles and manual or semi-automated readings (91).

Although elevated PTH levels are a defining feature of all PHPT phenotypes, the presence or absence of increased calcium levels distinguishes classic hypercalcemic from normocalcemic PHPT (3), and these two entities may exert distinct effects on the vasculature through the differential contribution of hypercalcemia. To investigate this possibility, we performed an exploratory subgroup analysis according to calcemic status for CCA IMT data, which showed similar effect sizes and no significant difference between normocalcemic and hypercalcemic PHPT subgroups. Moreover, sensitivity analyses in which the normocalcemic and hypercalcemic strata from Cansu et al. (52) were entered separately instead of the combined cohort, as well as analyses excluding the study presenting only patients with normocalcemic PHPT (57), did not materially alter the pooled CCA IMT estimate. In the meta-regression analysis, mean calcium levels did not emerge as a significant moderator. These findings suggest that the currently available evidence does not support a differential effect of PHPT on CCA IMT according to calcium status. Nevertheless, given the limited number of studies evaluating normocalcemic PHPT, the possibility of true phenotype-specific differences cannot be excluded.

In renal transplant recipients, carotid IMT correlated independently with circulating intact PTH and improved after correction of the hyperparathyroid state (71). In the present analysis, PTX was associated with a statistically significant reduction in carotid IMT at 6 months, with low heterogeneity across studies, supporting the hypothesis that correction of hyperparathyroidism might favorably influence early vascular remodeling. However, the long-term (>6 months) estimate was non-significant overall, showed substantial heterogeneity, and reversed direction upon exclusion of a single influential study (64). Given that a true structural improvement in arterial wall thickness would generally be expected to persist beyond the early postoperative period, the differences between the short- and long-term findings are more likely to reflect methodological heterogeneity—variations in patient selection, follow-up duration, carotid segment and side of measurement, and the small number and size of the available longitudinal studies—rather than a time-dependent change in the effect of PTX on CCA IMT. Collectively, these findings suggest that while PTX may exert short-term favorable effects on carotid wall thickness, the durability and magnitude of this effect remain uncertain. Larger, well-standardized longitudinal studies with uniform follow-up intervals are needed to clarify the temporal vascular impact of surgery in PHPT.

It was suggested that a risk stratification strategy that combines both maximal plaque thickness and mean CCA IMT, used alongside traditional risk factors, might improve identification of individuals who would benefit from pharmacological and lifestyle interventions (92, 93). In the Manhattan study, high serum calcium was linked to greater carotid plaque thickness in a multi-ethnic sample of older adults (94). Notably, in two studies included in this review, no differences were observed in maximum plaque thickness between PHPT patients and controls (45) or between PHPT patients with levels of 25(OH) vitamin D below or above 50 nmol/L (35).

The present analysis did not identify significant differences regarding plaque prevalence between patients with PHPT and controls, which might indicate that PHPT may preferentially contribute to diffuse arterial wall thickening rather than advanced focal atherosclerotic plaque formation. However, this result should be interpreted with caution given the small number of studies included and the limited total sample size. In addition, plaque assessment is inherently more variable than carotid IMT and depends on differences in plaque definition, imaging protocols, and population risk profiles across studies (95, 96). Due to the various definitions used in the included studies, some emphasizing focal morphology and others relying primarily on thickness threshold, it is likely that they identified different degrees of lesion burden.

### 4.2 Functional changes

FMD of the brachial artery has been reported as an independent predictor of CV events in several investigations (97–99) although this association has not been consistently observed across all studies (100, 101). Yeboah et al. attributed the heterogeneity in findings to inherent biological and technical variability in FMD assessment, the inclusion of highly selected participant groups in some cohorts, and inadequate statistical power in some analyses (99). One study found that acute hypercalcemia was associated with dose-dependent impairment of endothelial vasodilatory function (102). Moreover, associations between elevated PTH levels and reduced brachial artery FMD have been reported in several studies (46, 51, 53, 54, 74, 103); however, this relationship has not been consistently observed across investigations (104).

The present meta-analysis demonstrated a marked reduction in FMD among patients with PHPT, indicating clinically meaningful endothelial dysfunction. Despite considerable between-study heterogeneity, the direction and statistical significance of the pooled effect were stable across sensitivity analyses, supporting a genuine association.

Normal aging is linked to a gradual decline in endothelial function, and this deterioration tends to begin earlier in men than in women (105). Meta-regression identified mean age as a significant moderator, with attenuation of the PHPT–control difference in older cohorts. This pattern may reflect age-related endothelial impairment in control groups that reduces the observable contrast with PHPT, although residual confounding related to study-level analyses cannot be excluded (106).

A key question is whether the vascular alterations observed in PHPT represent a direct consequence of the disease or are primarily explained by the higher prevalence of traditional CV risk factors reported in several of the included cohorts (32, 33, 47, 49–53, 57). To address this potential source of confounding, for FMD and CCA IMT we performed subgroup analyses stratified according to the burden of CV risk factors. For both outcomes, the association between PHPT and vascular alterations remained statistically significant in studies including patients with a substantial burden of CV risk factors as well as in those enrolling populations largely free of such risk factors, with no statistically significant difference between subgroups. Furthermore, meta-regression analyses did not identify blood pressure, BMI, or lipid levels as significant moderators. Taken together, these findings suggest that the observed differences in vascular phenotype cannot be explained solely by the burden of conventional CV risk factors. Nevertheless, this conclusion should be interpreted with caution, as the number of studies within each subgroup was limited, the definitions and prevalence of CV risk factors varied across studies, and residual or unmeasured confounding, including factors such as insulin resistance or physical inactivity, cannot be excluded.

Variability in brachial artery FMD assessment methodology across the included studies likely contributed to between-study heterogeneity as this parameter is highly sensitive to acquisition protocols, including participant preparation, cuff position and duration, US methodology, and analytic approach, as highlighted in methodological guidelines (107, 108). The main differences found in the evaluated studies included transducer frequency, cuff position (forearm versus upper arm), occlusion pressure and duration, and the timing and frequency of post-deflation diameter measurements. Peak FMD was most commonly obtained at ~ 60 seconds, but several studies performed serial measurements over longer intervals, which is relevant because time-to-peak dilation varies substantially between individuals (107). Measurements were generally performed on the right brachial artery, and exclusion of the study by Ekmekci et al. (46), which did not report the measurement side, did not significantly reduce heterogeneity in the leave-one-out analysis. Differences in ECG gating, operator blinding, fasting and medication withdrawal protocols, and whether automated or manual edge detection was used could represent additional sources of variability. Similar to the carotid IMT meta-analysis, heterogeneity might have been amplified by variability in disease duration, the need to convert provided data into means and SDs and the relatively small samples of some studies.

Several guidelines provide standardized recommendations for brachial artery FMD assessment to improve reproducibility and comparability across studies. The Corretti et al. statement provided the first widely adopted framework for standardized brachial artery FMD assessment (108). It recommended strict participant preparation, high-resolution longitudinal US with ECG-gated diameter measurements, induction of reactive hyperemia using ~5 minutes of suprasystolic cuff inflation and expressing FMD as percent change from baseline. The guideline also recommended assessing NMD whenever possible. The Thijssen et al. guideline expanded and refined earlier recommendations by incorporating physiological insights and updated methodological standards (107). It strongly advocated continuous or repeated post-deflation diameter measurements to capture true peak dilation, highlighted the importance of shear stimulus quantification, rigorous quality control, standardized subject preparation, and detailed reporting of protocols and discussed the influence of cuff position on NO dependency of the response (107).

PTX was associated with a directional increase in FMD at 6 months postoperatively, but the pooled effect was not statistically significant and remained highly heterogeneous. Sensitivity analysis including all follow-up durations suggested a trend toward improvement as well. Exclusion of the study by Neunteufl et al. (59) from this analysis rendered the effect statistically significant while maintaining the same direction, likely reflecting the study’s markedly longer follow-up (~3 years) and small sample size. Overall, these findings indicate a possible postoperative improvement in endothelial function, but larger prospective studies with standardized brachial FMD measurement protocols and prespecified follow-up intervals are needed to confirm the magnitude and durability of the effect.

NMD was found to be an independent predictor of future CV events (109) and one study suggested that the combined assessment with FMD might improve the prediction of CV events compared with FMD alone (25). One study even reported that a larger brachial artery diameter and a reduced vasodilatory response to nitroglycerin, but not impaired FMD, were associated with subclinical coronary atherosclerosis (110). Notably, the diameter of the brachial artery did not differ significantly between PHPT patients and controls and was not significantly influenced by PTX in the studies included in the present systematic review. NMD is commonly used to verify that a reduced FMD response reflects endothelial dysfunction rather than impaired smooth muscle function (111). In contrast to the marked impairment observed in FMD, NMD did not differ significantly between PHPT patients and controls and was not influenced by PTX, suggesting preserved endothelium-independent vasodilatory function.

The discordance between impaired FMD and preserved NMD supports a predominantly endothelial mechanism of vascular dysfunction in PHPT rather than primary VSM impairment. Although the number of available studies is limited, these observations are broadly consistent with experimental evidence showing that local brachial artery infusion of PTH does not significantly alter endothelium-independent vasodilation to nitroprusside, while acute hypercalcemia may even enhance such responses (102, 112). Further evidence is provided by Nilsson et al. (38), who assessed vascular function using forearm venous occlusion plethysmography with intra-arterial infusion of methacholine and sodium nitroprusside. Patients with PHPT exhibited a significantly reduced endothelial function index compared with matched controls, but this difference appeared to be driven primarily by relatively increased endothelium-independent responses rather than a marked reduction in endothelium-dependent vasodilation. Importantly, in this study, endothelial function normalized following PTX, suggesting that these alterations are at least partially reversible (38). Similarly, Broulik et al. reported increased resting blood flow in the calf and forearm of PHPT patients, consistent with reduced peripheral vascular resistance (113).

### 4.3 Potential mechanisms for structural and functional changes

**Figure 12:**
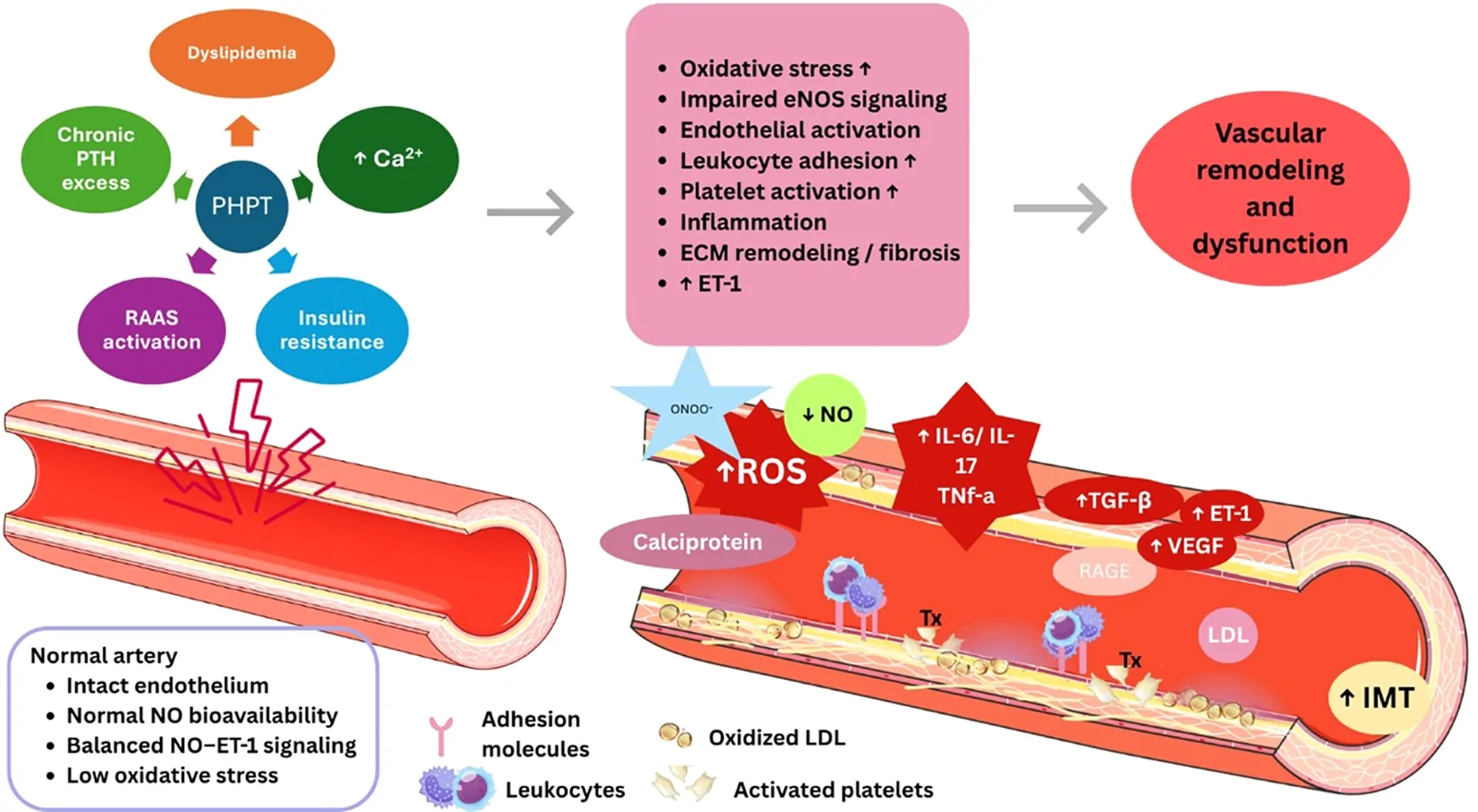
Mechanisms linking PHPT to vascular dysfunction and increased intima-media thickness. The figure illustrates the transition from a physiologically normal vessel to a state of endothelial dysfunction and early vascular remodeling in PHPT. Under normal conditions, vascular homeostasis is maintained by adequate nitric oxide (NO) bioavailability, low oxidative stress, minimal cellular adhesion and an intact endothelium. In PHPT, the combined effects of PTH excess, hypercalcemia, and associated metabolic and inflammatory disturbances promote oxidative stress, reduce NO bioavailability, increase endothelial expression of adhesion molecules and receptor for advanced glycation end-products (RAGE) and enhance platelet activation. These changes alter vascular permeability and enhance leukocyte and platelet–endothelial interactions, deposition of oxidized LDL particles and inflammation-driven extracellular matrix remodeling and fibrosis, ultimately leading to endothelial dysfunction and increased arterial wall thickness. Tx, thromboxane; IMT, intima media thickness; ROS, reactive oxygen species; NO, nitric oxide; ONOO^-^, peroxynitrite; eNOS, endothelial nitric oxide synthase; Ca2+, calcemia; RAAS, Renin-Angiotensin-Aldosterone System; ET-1, endothelin 1; ECM, extracellular matrix; VEGF, vascular endothelial growth factor; TGF-β, transforming growth factor-β; RAGE, receptor for advanced glycation end-products; IL-6, interleukin-6; IL-17, interleukin-17; TNFα, tumor necrosis factor α.

PHPT appears to exert complex effects on the vasculature, potentially mediated by the combined influence of PTH, hypercalcemia, and accompanying metabolic and inflammatory alterations (**Figure 12**).

The vascular actions of PTH are likely dependent on the duration of exposure, with acute and chronic elevations exerting distinct effects. Under normal physiological conditions, particularly during short-term exposure, PTH and PTH-related peptide (PTHrP) have been shown to induce vasodilation and reduce blood pressure (114–116). One proposed mechanism involves the inhibition of endothelin-1 (ET-1), a potent vasoconstrictor, with evidence demonstrating an inverse correlation between circulating levels of these two molecules (117, 118). As the vasorelaxant effects of PTH do not appear to depend on an intact endothelial layer, it has also been hypothesized that this hormone could act directly on VSM cells, likely through inhibition of L-type calcium channels (119). In contrast to its acute vasodilatory actions, chronic PTH excess, as observed in PHPT, appears to shift the profile toward vascular dysfunction. Continuous PTH infusion in healthy individuals has been associated with increased blood pressure and it was proposed that prolonged exposure to PTH could attenuate subsequent vasodilatory responses through a mechanism called “homologous desensitization” (120–122). Elevated ET-1 levels reported in PHPT may further counteract PTH-mediated vasodilation (123). Findings from human VSM cell studies suggest that impaired VSM responsiveness is unlikely to be the primary mechanism underlying vascular alterations in PHPT (124). Furthermore, while proliferative effects of PTH on VSM cells have been demonstrated in animal models, such findings have not been clearly established in humans (124, 125).

Endothelium-derived NO is essential for vascular homeostasis, regulating vascular tone, inhibiting platelet aggregation and adhesion of lymphocytes, monocytes or granulocytes to the endothelium, while also acting as a free radical scavenger (126). In VSM cells, NO promotes vasodilation through activation of soluble guanylate cyclase and increased cGMP, which in turn leads to inhibition of voltage-gated Ca²^+^ channels, with decreased Ca²^+^ entry in the cells and increased sequestration in the sarcoplasmic reticulum (127, 128). Impaired NO signaling is linked to hypertension and vasospasm (129, 130). Although PTH appears to be able to stimulate endothelial nitric oxide synthase (eNOS) and increase NO production (131), chronic exposure likely leads to adverse effects. Sustained NO generation in the presence of increased oxidative stress could promote the formation of reactive nitrogen species such as peroxynitrite, leading to reduced NO bioavailability (132). Moreover, this highly reactive compound induces protein nitration, lipid peroxidation, and mitochondrial dysfunction (132–135), endothelial cells being more susceptible to peroxynitrite-mediated damage, compared with VSM cells (135). Endothelial injury, together with reduced availability of NO could therefore lead to impaired endothelium-dependent vasodilation. Supporting this, reduced NO bioavailability and a compensatory shift toward alternative vasodilatory pathways, such as endothelium-derived hyperpolarizing factors, have been demonstrated in PHPT, with restoration of NO-dependent responses following PTX (58, 136, 137).

Oxidative stress was found to be increased in PHPT (58, 138). This process is widely recognized as a key driver of atherogenesis and could represent a central mechanism linking PTH excess to vascular dysfunction. Experimental evidence shows that PTH increases reactive oxygen species (ROS) production (139). This increase was also documented in endothelial cells, leading to oxidative modification of specific membrane receptors. In particular, oxidation of the bradykinin B2 receptor impairs bradykinin-induced Ca²^+^ signaling required for eNOS activation and NO production, while acetylcholine-mediated signaling remains largely unaffected (140). Oxidized forms of PTH appear to exhibit greater pathogenic relevance for CV disease than the native hormone, further linking oxidative stress to CV risk (141). Therefore, it was proposed that distinguishing between different PTH subtypes is important when evaluating the role of this hormone as a CV risk factor (142).

Chronic low-grade inflammation could also contribute to increased oxidative stress, as pro-inflammatory cytokines such as interleukin-6 (IL-6), interleukin-17 (IL-17), and tumor necrosis factor (TNF)-α were found to be increased in patients with PHPT (143–145) and were also shown to induce ROS production in vascular cells (146–148). Moreover, *in vitro*, PTH increased the endothelial expression of molecules with proinflammatory and proatherogenic properties (149, 150) such as the receptor for advanced glycation end-products (RAGE) and IL-6, via PKC/PKA-dependent and NO–related pathways (151). Inflammation also initiates signaling pathways that promote fibrosis and vascular remodeling (152). For instance, IL-6 enhances the expression of transforming growth factor-β (TGF-β), which drives fibroblast activation, increasing the synthesis of extracellular matrix proteins and integrins while reducing matrix metalloproteinase activity, ultimately leading to fibrosis (153). Moreover, PTH also upregulates vascular endothelial growth factor (VEGF)-165 mRNA expression, which may promote the initiation and progression of vascular remodeling (8, 154). Evidence regarding the effects of PTX on inflammatory markers is conflicting, with some studies reporting partial improvement (155, 156), while others showed no significant benefit (157, 158).

Hypercalcemia provides an additional and synergistic mechanism. Elevated extracellular calcium can directly activate inflammatory signaling pathways in endothelial cells, increasing the expression of adhesion molecules and leukocyte adhesion (159). In addition, hypercalcemia could also impair NO metabolism and induce oxidative stress through formation of calciprotein particles (160). Calcium homeostasis is also known to be important for regulating endothelial permeability and maintaining its integrity (161). It was proposed that elevated ionized serum calcium could increase vascular sensitivity to vasoconstrictors and activate pathways such as TGF-β, promoting fibrosis and arterial stiffening (162). A large epidemiological study showed that serum calcium levels within the normal range were positively associated with carotid plaque thickness, independent of traditional CV risk factors (94).

PHPT appears to be associated with a pro-adhesive vascular phenotype, characterized by increased markers of endothelial activation, including soluble E-selectin and von Willebrand factor (58, 163, 164) alongside enhanced platelet activation with elevated soluble P-selectin and thromboxane B_2_ (58). The concomitant increase in adhesion molecules such as ICAM-1 and VCAM-1 (58) further supports augmented leukocyte and platelet–endothelial interactions, contributing to vascular inflammation and atherothrombotic risk.

Furthermore, metabolic disturbances frequently associated with PHPT, including insulin resistance and dyslipidemia (7, 165, 166) may further exacerbate these vascular alterations. Insulin resistance is known to promote endothelial dysfunction by increasing oxidative stress, reducing NO bioavailability, and activating inflammatory pathways within the vascular wall (167). In addition, hyperinsulinemia and associated metabolic disturbances can stimulate VSM proliferation and extracellular matrix deposition, contributing to vascular remodeling and arterial wall thickening (168). Moreover, elevated LDL cholesterol plays a central role in atherosclerosis by accumulating within the arterial wall and undergoing oxidative modification (oxidized LDL), which promotes vascular inflammation and endothelial dysfunction (169, 170).

It has been demonstrated that PTH promotes aldosterone secretion by increasing intracellular calcium levels in adrenal cells through binding to the PTH/PTHrP receptor, and indirectly by enhancing the effects of angiotensin II (171). In patients with PHPT, aldosterone levels correlate positively with preoperative PTH concentrations (172). PTH has also been shown to increase plasma renin activity in normotensive individuals, an effect that normalizes after PTX (173). Aldosterone can impair endothelial function by increasing oxidative stress and reducing NO bioavailability (174). In addition, aldosterone promotes vascular remodeling through mineralocorticoid receptor–mediated effects on VSM cells and extracellular matrix, which may contribute to arterial wall thickening (175, 176).

### 4.4 Strengths and limitations

This study provides a comprehensive and mechanistically integrated synthesis of vascular alterations in PHPT, incorporating both functional (FMD, NMD) and structural (IMT, plaque) markers, evaluated in comparison with controls as well as longitudinally following PTX. Rigorous sensitivity analyses—including leave-one-out procedures, alternative pre–post correlation assumptions, and subgroup substitutions—demonstrated that the direction of most associations was generally robust. Meta-regression analyses further enabled exploration of potential sources of heterogeneity, strengthening the interpretability of the findings. In addition, separation of endothelium-dependent and endothelium-independent vascular measures enabled more precise pathophysiological interpretation.

However, several limitations should be acknowledged. Many analyses were based on a small number of studies, limiting statistical power and the precision of pooled estimates. Subgroup analyses should be interpreted as exploratory considering the limited number of studies within each stratum, variability in the burden and severity of CV risk factors even among similarly categorized populations and the lack of consistent reporting of measurement side. Likewise, meta-regression analyses were underpowered due to the small number of studies included. The observational nature of most included studies precludes causal inference regarding the vascular effects of PHPT or the impact of PTX. Regarding the quality of the studies, while the majority were rated as “Fair” or “Good”, several methodological limitations—including small sample sizes, insufficient reporting of participation rates, limited adjustment for confounders, and lack of repeated exposure assessment and blinded outcome assessment —should be considered when interpreting the results of the included studies.

Substantial between-study heterogeneity was observed in several models and is likely multifactorial. Study-level methodological variability represents one of the principal limitations of the available literature on vascular biology in PHPT, affecting the precision and direct comparability of effect estimates. Adoption of standardized protocols—such as the Mannheim consensus (90) or ARIC protocol (91) for carotid IMT, and the Corretti (108) and Thijssen (107) recommendations for FMD and NMD—will be essential for future studies to generate estimates that are comparable across cohorts and improve the robustness of evidence synthesis.

Given the high heterogeneity and limited dataset, assessments of publication bias should also be interpreted with caution. For most outcomes, the 95% prediction interval (PI) crossed zero (**Figures 2**-**11**), highlighting the uncertainty regarding the effects that may be observed in future studies and the limited generalizability of the pooled estimates.

## 5 Conclusion

Our meta-analytic pattern suggests that PHPT is associated with both functional and structural vascular alterations, characterized by impaired endothelial function, as reflected by reduced FMD in the presence of preserved NMD, and increased carotid IMT without a clear increase in carotid plaque prevalence. Although PTX demonstrated a tendency toward improved vascular function and reduced carotid IMT, some of the observed effects failed to reach statistical significance or were not robust in sensitivity analyses. Consequently, the current evidence remains insufficient to draw firm conclusions regarding the magnitude and durability of the vascular effects of surgery.

The observed vascular alterations in PHPT may occur at an early, potentially reversible stage of the atherosclerotic process. Accordingly, assessment of endothelial function and subclinical vascular changes may provide additional information for CV risk stratification in patients with PHPT. Although current guidelines do not consider CV involvement an indication for surgery, these observations suggest that vascular markers could help identify patients who could benefit from closer monitoring or more individualized management. Future prospective studies employing standardized vascular assessment protocols are warranted to improve interstudy comparability and reproducibility, and to determine whether these vascular markers are associated with clinically meaningful differences in CV outcomes and could inform risk-adapted management strategies in patients with PHPT.

## Data Availability

The original contributions presented in the study are included in the article/**Supplementary Material**. Further inquiries can be directed to the corresponding author.
