# Supplementary material for: Changes in vascular structure and function in primary hyperparathyroidism: a systematic review and meta-analysis
Source: Front Endocrinol (Lausanne). 2026 Aug 11;17:1896180. doi: 10.3389/fendo.2026.1896180 (PMC13503531; doi:10.3389/fendo.2026.1896180)
Supplement: Supplementary file 1 [file SupplementaryFile1.docx]

Supplementary Material

**Supplementary Figure S1**. Funnel plot for studies evaluating common carotid artery IMT in PHPT patients compared to controls

**Supplementary Figure S2.** Subgroup analysis according to CV risk factors presence for common carotid artery IMT in PHPT patients compared to controls

**Supplementary Figure S3.** Subgroup analysis according to side of measurement for common carotid artery IMT in PHPT patients compared to controls

**Supplementary Figure S4.** Subgroup analysis according to PHPT phenotype for common carotid artery IMT in PHPT patients compared to controls

**Supplementary Figure S5**. Subgroup analysis according to cardiovascular risk factors presence for FMD in PHPT patients compared to controls

**Supplementary Figure S6**. Funnel plot for meta-analysis of flow-mediated dilation in PHPT patients compared to controls

**Supplementary Table S1. Inclusion and exclusion criteria, definitions and comments regarding CV risk factors, ultrasound assessment methodologies and other vascular outcomes evaluated in the studies**. ref, reference; PHPT, primary hyperparathyroidism; FMD, flow-mediated vasodilation; NMD, nitroglycerin-mediated dilation; US, ultrasound; IMT, intima-media thickness; PTX, parathyroidectomy; PWV, pulse wave velocity; NA, not available; SBP, systolic blood pressure; DBP, diastolic blood pressure; CV, cardiovascular; ACE, angiotensin converting enzyme; ARBs, angiotensin receptor blockers; IM-GSM, Intima-media gray-scale median; MEN, multiple endocrine neoplasia; GFR, glomerular filtration rate.

| First author (year)  [ref] | Inclusion/ exclusion criteria | Other measurements | Measurement technique | Definitions and comments regarding CV risk factors |
| --- | --- | --- | --- | --- |
| Neunteufl (1998)  [55] | Inclusion:  **PHPT:** Patients without evidence of coronary artery disease. All of them underwent bilateral cervical exploration with excision of abnormal parathyroid glands.  **Controls:** Individuals with normal calcium levels and normal coronary arteries on angiography performed for atypical chest pain.  Exclusion:  NA | Brachial artery diameter (mm):  PHPT: 4.1 ± 0.8; Controls: 3.9 ± 0.8  Hyperemia (%):  PHPT: 122 ± 38; Controls: 126 ± 63  Hyperemia diameter (mm):  PHPT: 4.6 ± 0.8; Controls: 4.4 ± 0.8  NMD diameter (mm):  PHPT: 4.6 ± 0.8; Controls: 4.5 ± 0.8  FMD/NMD*:  PHPT: 0.98 ± 0.19; Controls: 0.81 ± 0.25 | - Measurements performed after at least 10 minutes of rest in the supine position  - High-resolution US (Acuson 128XP/10, 7-MHz linear transducer): **right** brachial artery diameter assessed between media–adventitia interfaces, proximal to the bifurcation of the radial and the ulnar artery, at least three times at baseline and every 30 s following reactive hyperemia (250 mmHg cuff, 4.5 min), and every 30 s after sublingual nitroglycerin (0.8 mg; administered 20 min post-deflation) by a blinded operator  - Maximum FMD and NMD diameters were defined as the mean of 3 consecutive maximal values. FMD and NMD were calculated as percentage diameter change from baseline from repeated measurements. Reactive hyperemia was expressed as percent change in flow index versus baseline | Smokers: individuals who had smoked ≥1 cigarette/day for ≥1 year within the past 5 years  Hypertension: current use of antihypertensive medication or SBP ≥145 mmHg and/or DBP ≥95 mmHg  Hypercholesterolemia: total serum cholesterol level > 5.2 mmol/ l. |
| Nilsson (1999)  [112] | Inclusion:  **PHPT:** Patients referred for parathyroid surgery at the County Hospital of Sundsvall (Sweden)  **Controls:** Normocalcemic subjects randomly selected from the general population of Sundsvall and matched to patients by age and sex.  Exclusion:  NA | Endothelial function index*/**: 1) PHPT: 1.01 ± 0.26; Controls: 1.27 ± 0.31  2) Before PTX: 1.01 ± 0.26; After PTX: 1.31 ± 0.39 | - Participants were asked to discontinue β-blockers, calcium channel blockers, and ACE inhibitors one week before the study; however, withdrawal was not possible in six patients and five controls due to coronary artery disease  - High-resolution carotid ultrasound (HDI 3000/5000, ATL; 10.5/12.5-MHz broadband transducer): IMT measurements were obtained at the level of the **far wall** of the **common** carotid artery IMT measured **bilaterally** 1 cm proximal to the carotid bifurcation; the mean of left and right arteries was analyzed  - Forearm venous occlusion plethysmography: vasodilatory agents were infused through a cannula placed in the cubital artery, with the contralateral arm serving as the control. Bilateral forearm blood flow was assessed with mercury-in-silastic strain gauges during upper-arm cuff inflation (40 mmHg, ~4 inflations/min, each inflation ~ 7 sec). Endothelium-dependent vasodilation was evaluated during metacholine infusion (2 and 4 µg/min) and endothelium-independent vasodilation during sodium nitroprusside infusion (5 and 10 µg/min), each for 5 min with 20-min washout. Flow values were averaged from five recordings, and the endothelial function index was calculated as the ratio of peak metacholine- to nitroprusside-induced flow | Seven patients and seven controls were receiving anti-hypertensive therapy. One patient with borderline hypertension initiated treatment after surgery (ACE inhibitor and calcium channel blocker). |
| Neun  teufl (2000)  [54] | Inclusion:  **PHPT:** No evidence of coronary artery disease based on medical history and 12-lead electrocardiography. All patients underwent bilateral cervical exploration with removal of abnormal parathyroid glands resulting in normocalcemia.  Exclusion:  NA | Hyperemia (%):  Before PTX: 123 ± 42; After PTX: 130 ± 58  Hyperemia diameter (mm):  Before PTX: 4.6 ± 0.9; After PTX: 4.5 ± 0.8  NMD diameter (mm):  Before PTX: 4.6 ± 0.9; After PTX: 4.7 ± 0.6  FMD/NMD:  Before PTX: 0.98 ± 0.14; After PTX: 0.86 ± 0.29  Brachial artery diameter (mm):  Before PTX: 4.1 ± 0.8; After PTX: 4.1 ± 0.9 | - Measurements performed after withdrawal of all vasoactive drugs for at least 24 h and at least 10 minutes of rest in the supine position  - High-resolution US (7-MHz linear array transducer): The **right** brachial artery diameter assessed between media–adventitia interfaces, proximal to the bifurcation of the radial and the ulnar artery, at least three times at baseline and every 30 s after cuff deflation following supra-systolic compression of the upper arm (250 mmHg for 4.5 minutes), and after sublingual administration of 0.8 mg nitroglycerin. The highest three consecutive post-hyperemia and post-nitroglycerin values were averaged to determine maximum FMD and NMD diameters. FMD and NMD were calculated as percentage diameter change from baseline from repeated measurements. Blood flow velocity was recorded at baseline and within 15 s after cuff release, and a flow index was derived to quantify reactive hyperemia | Smokers: individuals who had smoked ≥1 cigarette/day for ≥1 year within the past 5 years  Hypertension: use of antihypertensive medication or SBP ≥145 mmHg and/or DBP ≥95 mmHg |
| Kosch (2000)  [56] | Inclusion:  **PHPT:** Patients admitted for PTX between December 1997 and August 1999, consecutively enrolled  **Controls:** Twenty healthy volunteers matched for age, sex, and blood pressure  Exclusion:  Individuals were excluded if they exhibited coronary heart disease, heart failure, valvular heart disease, cerebrovascular disease, peripheral arterial disease, diabetes mellitus, renal impairment (serum creatinine >1.2 mg/dL), hypercholesterolemia (cholesterol >230 mg/dL), if they were smoking, or using of antihypertensive medication or had a systolic blood pressure ≥140 mmHg or a diastolic blood pressure ≥90 mmHg | Carotid artery end-diastolic diameter (mm):  1) PHPT: 6 ± 2.62; Controls: 5.9 ± 1.34  2) Before PTX: 6 ± 2.62; After PTX: 6.3 ± 1.74  Brachial artery end-diastolic diameter (mm):  1) PHPT: 3.4 ± 0.87; Controls: 3.3 ± 1.34  2) Before PTX: 3.4 ± 0.87; After PTX: 3.5 ± 1.31  Absolute FMD (mm)*/**:  1) PHPT: 0.16 ± 0.26; Controls: 0.6 ± 0.45  2) Before PTX: 0.16 ± 0.26; After PTX: 0.58 ± 0.61  Absolute NMD (mm):  1) PHPT: 0.78 ± 0.35; Controls: 0.74 ± 0.49  2) Before PTX: 0.78 ± 0.35; After PTX: 0.76 ± 0.52  Brachial IMT (mm):  1) PHPT: 0.49 ± 0.22; Controls: 0.47 ± 0.27  2) Before PTX: 0.49 ± 0.22; After PTX: 0.47 ± 0.31  Reactive hyperemia (%):  1) PHPT: 447 ± 217.94; Controls: 462 ± 317.52  2) Before PTX: 447 ± 217.94; After PTX: 436 ± 270.25 | - Measurements were performed between 8 and 12 a.m. in supine position, after at least 15 min at rest  - Multigate pulsed Doppler US (Pie Medical, Scanner 2000; 7.5-MHz linear array probe) was used to image the **left common** carotid artery (1, 2, and 2.5 cm proximal to the bifurcation) and the **right** brachial artery (2–6 cm proximal to the elbow) in B/M-mode. End-diastolic diameters were measured using ECG triggering. Mean carotid IMT was calculated as the average of three measurements obtained at three sites of the **far** wall. Brachial artery diameter was recorded at baseline (mean of three measurements), during the final minute of cuff occlusion (300 mmHg for 4 min), and at 1, 2, 3, 5, 7, and 10 minutes after cuff deflation. Brachial artery flow was also measured at baseline and during the first 15 seconds of reactive hyperemia. After return to baseline (approximately 11 minutes), 0.4 mg sublingual glycerol trinitrate was administered and arterial diameter reassessed at 1, 3, and 5 minutes  - FMD and NMD were defined as the maximal absolute and relative increase in end-diastolic diameter from baseline. |  |
| Barletta (2000)  [177] | Inclusion:  **PHPT:** consecutive patients with **mild asymptomatic** PHPT diagnosed at the Ambulatory of Metabolic Bone Diseases, Endocrine Unit, University of Florence, between 1994 and 1996 during osteoporosis screening. All patients had persistently elevated serum calcium levels (2.62–3.15 mmol/L), inappropriately elevated PTH levels, and high-turnover bone disease (osteopenia/osteoporosis) in the absence of secondary causes of osteoporosis, fulfilling criteria for surgical treatment. PTX was performed 1- to-3 months after baseline evaluation  **Controls:** age- and sex-matched healthy subjects  Exclusion:  Individuals were excluded if they had impaired renal function, arterial hypertension (according to the 1993 WHO guidelines), other coexisting diseases or smoked >5 cigarettes/ day | Brachial artery end-diastolic diameter (mm):  1) PHPT: 3.2 ± 0.5; Controls: 3.5 ± 0.4  2) Before PTX: 3.4 ± 0.5; After PTX: 3.2 ± 0.6  Brachial IMT (mm):  1) PHPT: 0.48 ± 0.06; Controls: 0.48 ± 0.07  2) Before PTX: 0.48 ± 0.06; After PTX: 0.48 ± 0.05  Brachial artery distention (%):  1)PHPT: 16.6 ± 5.7; Controls: 15.5 ± 3.9  2) Before PTX: 16.6 ± 5.7; After PTX: 16.3 ± 6.5  Carotid artery end-diastolic diameter (mm):  1) PHPT: 5.7 ± 0.8; Controls: 5.3 ± 1.5  2) Before PTX: 5.6 ± 0.8; After PTX: 5.8 ± 0.9  Carotid artery distention (%):  1) PHPT: 11.9 ± 1.4; Controls: 12.4 ± 3.3  2) Before PTX: 11.9 ± 1.4; After PTX: 11.2 ± 3.5  PWV (m/sec):  1) PHPT: 8.8 ± 1.6; Controls: 8.2 ± 3.5  2) Before PTX: 9 ± 1.5; After PTX: 10 ± 4.4  Brachial artery compliance (cm/ mmHg):  1)PHPT: 3.2 ± 1.6; Controls: 3.9 ± 2.4  2) Before PTX: 3.3 ± 1.7; After PTX: 3 ± 1.7  Brachial artery stiffness index:  1) PHPT: 0.09 ± 0.04; Controls: 0.08 ± 0.02  2) Before PTX: 0.09 ± 0.04; After PTX: 0.09 ± 0.04  Brachial artery elastic modulus:  1) PHPT: 348.6 ± 159.1; Controls: 364.5 ± 103  2) Before PTX: 358.6 ± 159.1; After PTX: 441 ± 205.5  Carotid artery compliance (cm/ mmHg):  1) PHPT: 5.6 ± 2.9; Controls: 5.9 ± 4.4  2) Before PTX: 5.6 ± 3.2; After: 5.5 ± 3.1  Carotid artery stiffness index:  1) PHPT: 0.07 ± 0.01; Controls: 0.07 ± 0.02  2) Before PTX: 0.07 ± 0.01; After PTX: 0.06 ± 0.02  Carotid artery elastic modulus:  1) PHPT: 502 ± 55; Controls: 459 ± 154  2) Before PTX: 492 ± 15; After PTX: 642 ± 299 | - Previous medications were withheld ≥1 month before the study. Smoking was prohibited for 5 days before and during the study. Assessments were performed at 08:00 after overnight fasting  - US evaluation: end-diastolic measurements were obtained at the level of the **far wall** of the **left** **common** carotid artery, 1 cm proximal to bifurcation, and the **left** brachial artery at the elbow.  - Arterial distension was defined as the percentage increase in vessel diameter from diastole to systole  - Blood pressure values were used to derive the arterial elastic modulus and stiffness index  - Arterial compliance was calculated from pulse wave velocity, measured from simultaneous brachial and radial pulse recordings obtained with piezoelectric transducers, and adjusted for mean arterial diameter and blood density |  |
| Nuzzo (2002)  [50] | Inclusion:  **PHPT:** consecutive patients with untreated PHPT, evaluated at the Department of Molecular and Clinical Endocrinology and Oncology, Federico II University, Naples. PHPT was diagnosed based on persistently elevated serum calcium levels and inappropriately high intact PTH levels. The mean interval between first detection of hypercalcemia and study enrollment was 10 months  **Controls:** healthy subjects matched for age, sex, and body mass index recruited among individuals referred for thyroid ultrasonography without relevant pathology  Exclusion:  Individuals were excluded if they had hypertension (according to the 1993 WHO guidelines), elevated pancreatic enzyme levels, impaired renal function, coronary artery disease, or a history of prior neck surgery |  | High-resolution carotid echo-Doppler US (10-MHz transducer): **bilateral** longitudinal scans of the **common** carotid artery, carotid **bulb**, and **proximal internal** carotid artery. M-mode recordings obtained after B-mode optimization. IMT was measured at the site of maximal thickness from three imaging angles; each measurement was performed three times, and the mean of the obtained values was calculated  - IMT was categorized as normal (<1 mm), increased (1–1.3 mm), or suggestive of stenosis (>1.3 mm)  - Plaques (presence, size, location) were assessed in all segments  - All measurements were performed by a single blinded operator and images stored for offline analysis | Among the included participants who smoked, all were defined as mild smokers (<10 cigarettes/day).  None of the participants were receiving CV medications |
| Fallo (2003)  [49] | Inclusion:  **PHPT:** patients with solitary parathyroid adenomas consecutively enrolled and divided into two groups**.** The proportion of patients with mild hyperparathyroidism (serum calcium ≤12 mg/dL) was similar in these groups.  **Group 1:** patients without classical CV risk factors  **Group 2:** patients with one or more CV risk factors.  **Controls:** subjects evaluated for dizziness, cervical bruits, or headache, age- and sex- matched patients from group 2, but also statistically comparable (age, sex, and BMI) with patients from group 1.  Exclusion:  Individuals were excluded from group 1 if they exhibited CV risk factors (diabetes, hyperlipidemia, hypertension, obesity, or smoking), coronary or peripheral artery disease, prior CV or cerebrovascular events, or renal impairment (creatinine >1.2 mg/dL). Patients with heart failure were excluded from both groups. | Carotid artery end-diastolic diameter (mm):  PHPT 1: 6.37 ± 0.54; PHPT 2: 5.7 ± 0.64; Controls: 6.46 ± 0.6 | - High-resolution B-mode US (Sonos 5000, Hewlett-Packard; 7.5-MHz probe): bilateral longitudinal images of the distal 20 mm of the **common** carotid arteries, proximal to the bulb, were obtained at end-diastole. Three measurements of IMT and lumen diameter at each artery were averaged  - IMT was defined as the distance between the lumen–intima and media–adventitia interfaces of the **far wall**  - Mean IMT= the average of all measurements from both carotid arteries; maximum IMT = the average of the highest IMT measurement from each side  - Carotid plaque defined as far-wall IMT ≥1.3 mm at any carotid segment  - All measurements were performed by a single trained physician blinded to case–control status, with an intra-observer coefficient of variation <5% | Diabetes mellitus: use of hypoglycemic medications or fasting plasma glucose ≥126 mg/dL (7.0 mmol/L) on two separate measurements.  Hyperlipidemia: fasting total cholesterol >220 mg/dL (5.7 mmol/L) and/or triglycerides >1.56 mmol/L, or use of lipid-lowering therapy.  Hypertension: use of antihypertensive medication or systolic blood pressure ≥140 mmHg and/or diastolic blood pressure ≥90 mmHg. Secondary causes of hypertension were excluded through biochemical, hormonal and instrumental evaluation  Obesity: BMI ≥30 kg/m².  Smokers: smoked at least one cigarette per day for 1 year within the previous 5 years |
| Lumachi (2006)  [58] | Inclusion**:**  **PHPT:** patients with biochemically confirmed PHPT prospectively enrolled  **Controls:** age- and sex-matched healthy volunteers  Exclusion:  NA |  | - Measurements performed in supine position  - High-resolution carotid B-mode US (Esaote AU4, Genova, Italy; 7.5-MHz linear probe): **bilateral** longitudinal scans of the **near and far walls** of the distal **common** carotid artery (last 1 cm), **bifurcation**, and **proximal internal** carotid artery (first 1 cm) were performed with subjects supine, using anterior, lateral, and posterior projections. IMT and lumen diameter were measured in real time on unfrozen images. Three measurements per segment were averaged (between the lumen-intima and the media-interfaces), and maximal IMT per segment recorded. |  |
| Baykan (2007)  [59] | Inclusion**:**  **PHPT:** patients without a history or clinical signs of coronary heart disease or congestive heart failure, with ECG showing sinus rhythm without ischemic changes, prospectively enrolled  Exclusion:  Individuals were excluded if they had diabetes mellitus, hypertension, coronary heart disease, bundle branch block, atrial fibrillation, paced rhythm, atrioventricular block, moderate–severe valvular heart disease, restrictive, hypertrophic or dilatative cardiomyopathies, congenital heart disease, hyperthyroidism and hypothyroidism, were smokers, or were receiving CV medications (ACE inhibitors, ARBs, calcium channel blockers, beta blockers, or statins**)** | Brachial artery end-diastolic diameter (mm):  PHPT: 0.33 ± 0.42; Control: 0.33 ± 0.59  Hyperemia diameter (mm):  PHPT: 0.36 ± 0.92; Controls: 0.39 ± 0.63 | - Participants fasted for at least 8 hours before the study and were examined in a quiet, temperature-controlled room. Caffeine intake and smoking were prohibited for at least 4–6 hours before the assessment. The right arm was immobilized using 2 cushions supporting the elbow and the wrist.  - High-resolution B-mode US: the **right** brachial artery imaged longitudinally with the probe fixed in position. Three baseline ECG-gated end diastolic diameters were obtained, followed by forearm cuff–induced ischemia (100 mmHg greater than SBP, 5 min) and serial post-deflation measurements at 30 s, 1 min, 2 min, 3 min, and 4 min.  - FMD was calculated as the percent change from baseline to maximal post-hyperemia diameter, with the 1-min value used as the index of endothelial function. |  |
| Walker (2009)  [43] | Inclusion**:**  **PHPT:** patients aged 45–75 years with **mild hypercalcemia** (serum calcium >10.2 and <12.0 mg/dL) and elevated or inappropriately normal PTH levels, consecutively recruited from the Metabolic Bone Diseases Unit at Columbia University Medical Center. The calcium/creatinine clearance ratio was measured to exclude familial hypocalciuric hypercalcemia, and none had thiazide-induced hypercalcemia  **Controls:** selected from the Northern Manhattan Study (NOMAS), a population-based cohort investigating vascular risk factors in 3298 stroke-free individuals. All NOMAS subjects had normal serum calcium levels, were aged 45–75 years, and were of similar race/ethnicity to cases (Caucasian Hispanic or non-Hispanic)  Exclusion:  Individuals were excluded if they had current bisphosphonate therapy and initiation or modification of lipid-lowering treatment within the previous 2 years | Maximal carotid plaque thickness (mm):  PHPT: 2.09 ± 1; Controls: 2.08 ± 0.76  Carotid strain (%):  PHPT: 9.1 ± 21.5; Controls: 8.5 ± 5  Carotid stiffness*:  PHPT: 6.2 ± 24.2; Controls: 8.3 ± 5.8  Carotid distensibility:  PHPT: 0.19 ± 0.54; Controls: 0.17 ± 0.13 | - High-resolution carotid B-mode US (GE Healthcare LogIQ 700; 9/13-MHz transducer): both extracranial carotid arteries were examined in supine subjects using standardized axial and longitudinal views. Resting brachial blood pressure was measured before and after imaging and averaged.  - IMT was assessed **bilaterally** as a composite of **near and far** walls of the **common** carotid artery, **bifurcation**, and **internal** carotid artery. Values > 0.9 mm were considered abnormal  - Carotid plaque was defined as focal wall thickening >50% greater than adjacent wall, and maximal plaque thickness was determined from multi-angle images using semiautomated software  - Carotid mechanics were evaluated in the 10-mm segment of the right CCA below the origin of the carotid bulb by measuring systolic and diastolic diameters over 5 consecutive cardiac cycles to derive strain, stiffness index, and IMT-adjusted distensibility; lower strain or distensibility and higher stiffness indicated impaired vascular compliance. Stiffness ≥ 6, strain < 6%, distensibility ≤ 0.425 = abnormal. | Hypertension: systolic pressure was ≥140 mmHg, diastolic pressure ≥90 mmHg, history of hypertension or use of antihypertensive drugs  Coronary artery disease: history of myocardial infarction, angina, angioplasty, or coronary artery bypass surgery  Hypercholesterolemia: elevated lipid levels or being on a lipid-lowering medication  Diabetes mellitus: fasting plasma glucose ≥126 mg/dL or a self-reported diagnosis or treatment with insulin or oral glucose-lowering agents.  Adjustment for sex, diabetes status, systolic and diastolic blood pressure, triglyceride levels, and body mass index was performed. |
| Ekmekci (2009)  [52] | Inclusion:  **PHPT:** patients prospectively recruited from the Endocrinology Clinic of Internal Medicine at Istanbul Faculty of Medicine. All patients fulfilled NIH 2002 criteria for PTX. Parathyroid adenomas were successfully removed in all cases  **Controls:** healthy participants matched for age, sex, body mass index (BMI), lipid profile, and blood pressure  Exclusion:  Individuals were excluded if they had diabetes mellitus, hypertension, renal failure, established CV disease, other chronic conditions affecting endothelial function, or were using medications known to influence endothelial function |  | - Assessments were performed on an outpatient basis after a 12-hour overnight fast. Menstruating women were examined during the early follicular phase of the menstrual cycle. None of the postmenopausal patients and controls were receiving estrogen replacement therapy. Measurements were performed in the supine position after 10 min rest and participants abstained from smoking and caffeine before testing  - High-resolution US B-mode assessment (10-MHz linear transducer, Vingmed System Five): the **dominant** brachial artery was imaged longitudinally, and three measurements of ECG-gated end-diastolic diameters were recorded at baseline and after forearm cuff–induced ischemia. Reactive hyperemia was generated by 5-min cuff inflation up to 200 mmHg, with arterial diameter measured 60 s after deflation. FMD was expressed as the percent change from baseline diameter  - Measurements were obtained by a single experienced operator with an intra-observer variability of 3.3% |  |
| Ring (2012)  [46] | Inclusion:  **PHPT:** patients with **mild PHPT** consecutively enrolled  **Controls:** age- and sex-matched healthy subjects randomly selected from the Stockholm population registry. Potential participants were contacted by mail and invited to participate. Those who met the predefined inclusion criteria were enrolled, and if a selected individual declined participation, another randomly selected control was contacted  Exclusion:  Individuals were excluded if serum calcium was ≥3.0 mmol/L, or if they had familial hypocalciuric hypercalcemia, hypertension, diabetes mellitus, renal disease, were using medications affecting the CV system, were current smokers, had BMI ≥28 kg/m², or were younger than 18 or older than 70 years. Additionally, several patients were excluded due to withdrawal from surgery, carotid image storage failure, or use of lipid-lowering therapy | IM-GSM (mean):  1) PHPT: 82.3 ± 17.2; Controls: 86.5 ± 15.3  2) Before PTX: 82.3 ± 17.2; After PTX: 81.8 ± 15.9  Radial IMT (mm):  1) PHPT: 0.27 ± 0.06; Controls: 0.26 ± 0.05  2) Before PTX: 0.27 ± 0.06; After PTX: 0.27 ± 0.05  Radial artery end-diastolic diameter (mm)**:  1) PHPT: 1.79 ± 0.3; Controls: 1.87 ± 0.35  2) Before PTX: 1.79 ± 0.3; After PTX: 1.9 ± 0.38  Augmentation Index (%):  1) PHPT: 28.6 ± 12.2; Controls: 27.7 ± 12.8  2) Before PTX: 28.6 ± 12.2; After PTX: 29.8 ± 11.9  Aortic PWV (m/s)*:  1) PHPT: 8.68 ± 1.5; Controls: 8.13 ± 1.55  2) Before PTX: 8.68 ± 1.5; After PTX: 8.61 ± 1.37  Aortic SBP (mmHg):  1) PHPT: 114.4 ± 17.3; Controls: 109.8 ± 15.3  2) Before PTX: 114.4 ± 17.3; Afte PTX: 114.8 ± 15.8  Aortic DBP (mmHg):  1) PHPT: 78.1 ± 11.3; Controls: 75 ± 9.7  2) Before PTX: 78.1 ± 11.3; After PTX: 78 ± 8.7  Carotid artery end-diastolic diameter (mm):  1) PHPT: 6.03 ± 0.45; Controls: 5.94 ± 0.62  2) Before PTX: 6.03 ± 0.45; After PTX: 6.03 ± 0.47 | - Measurements performed in the morning after an overnight fast, following a 30–60-minute rest in the supine position. US examinations and pulse wave analyses were conducted by the same investigator  - B-mode US (Vivid 7, GE Healthcare, Horten, Norway; 8-MHz linear transducer): **bilateral** longitudinal images of the **common** carotid artery were acquired 1–2 cm proximal to the carotid bulb at end-diastole (ECG R-wave). A 10-mm region of interest was analyzed using automated edge-detection software (Artery Measurement Software). Mean IMT was defined as the distance between the lumen–intima and media–adventitia interfaces of the **far wall**, and lumen diameter between near- and far-wall interfaces. IM-GSM was derived from grayscale pixel analysis (0–255), using blood and adventitia as references.  - Applanation tonometry with pulse wave analysis (SphygmoCor system, AtCor Medical, Sydney, Australia; Millar SPT-301B tonometer): radial artery waveforms were recorded noninvasively and transformed into central aortic waveforms using a validated transfer function. Augmentation index was calculated as the difference between the first and second systolic peaks of the aortic waveform, expressed as a percentage of pulse pressure. Aortic pulse wave velocity was obtained from sequential carotid and femoral recordings using ECG R-wave gating; transit time was calculated between sites and path length derived from body surface measurements using a standardized formula. PWV was calculated as 0.8 × distance/transit time (m/s). Measurements were calibrated to brachial blood pressure (Omron M7), and the mean of two recordings was used.  - Ultra–high-resolution radial artery US (Vevo 770, VisualSonics, Toronto, Canada; 55-MHz transducer): longitudinal B-mode images of the right radial artery were obtained 1–2 cm proximal to the wrist crease. Intima-media thickness of the far wall was measured at end-diastole using the leading-edge principle, based on nine measurements from three representative images. Lumen diameter was assessed from M-mode images as the distance between near- and far-wall lumen interfaces, averaged over three cardiac cycles. |  |
| Walker (2012)  [61] | Inclusion:  **PHPT:** patients aged 45–75 years with **mild PHPT** (serum calcium levels between 2.55 and 3.0 mmol/L and elevated or inappropriately normal PTH levels)  Exclusion:  Individuals were excluded if they had familial hypocalciuric hypercalcemia, thiazide- or lithium-associated hyperparathyroidism, current bisphosphonate therapy, or recent initiation or modification of lipid-lowering treatment within the preceding two years | Maximal carotid plaque thickness (mm):  Before PTX: 0.89 ± 0.46; 12 mo after PTX: 1.07 ± 0.45; 24 mo after PTX: 1 ± 0.43  Carotid plaque number:  Before PTX: 0.95 ± 1.59; 12 mo after PTX**: 1.19 ± 1.54; 24 mo after PTX: 1.14 ± 1.48  Carotid stiffness:  Before PTX: 6.3 ± 2.65; 12 mo after PTX: 6.4 ± 2.56; 24 mo after PTX: 5.2 ± 2.46 | - High-resolution B-mode US (LogIQ 700, GE Healthcare; 9/13-MHz transducer): **bilateral** carotid arteries were evaluated by a trained sonologist using the NOMAS validated protocol. IMT was assessed as a composite of **near and far wall** measurements from the **common** carotid artery, **bifurcation**, and **internal** carotid artery on both sides, with IMT ≥0.9 mm considered abnormal  - Carotid plaque was defined as focal wall thickening >50% greater than adjacent wall, and maximal plaque thickness was quantified from the site of greatest prominence using semiautomated software  - Carotid stiffness was determined in a standardized segment of the right common carotid artery by measuring systolic and diastolic diameters over consecutive cardiac cycles to calculate a stiffness index, with higher values indicating reduced arterial compliance | Hypertension: systolic pressure was ≥140 mmHg, diastolic pressure ≥90 mmHg, history of hypertension or use of antihypertensive drugs.  Diabetes mellitus: fasting plasma glucose ≥126 mg/dL or a self-reported diagnosis or treatment with insulin or oral glucose-lowering agents.  Lipid-lowering therapy was adjusted during follow-up at the discretion of the treating physician |
| Petramala (2012)  [33] | Inclusion**:**  **PHPT:** consecutive patients with newly diagnosed asymptomatic PHPT evaluated at the Department Unit of Secondary Hypertension, Sapienza University of Rome, Italy. Diagnosis was based on persistently elevated total calcium, ionized calcium, and PTH levels. All patients underwent neck ultrasonography and/or Tc99m-sestamibi scintigraphy. Patients were considered asymptomatic in the absence of clinical complications such as kidney stones or radiologically evident bone lesions. Enrolled patients had histopathologically confirmed single adenoma and lack of recurrence after follow-up.  **Controls:** 1. healthy subjects  2. patients with essential hypertension  Exclusion:  Individuals were excluded if they had MEN 1 or MEN 2A, renal insufficiency, diabetes mellitus, thyroid dysfunction or major CV disease. None of the control subjects exhibited hypertension, metabolic syndrome, diabetes mellitus, CV diseases or secondary hypertension. | Carotid plaque prevalence (%):  1) PHPT: 9.7; Controls: NA  2) Before PTX: 9.7; After PTX: NA | - High-resolution US: both carotid arteries were examined **bilaterally,** including the **common** carotid artery, carotid **bifurcation**, and **internal** carotid artery. Mean carotid IMT was calculated as the average of 36 measurements obtained from the **near and far** walls of these segments on both sides, with three sampling points per segment  - Carotid plaques were defined as focal protrusions exceeding 50% of the surrounding arterial wall. Arterial wall thickening was defined as mean carotid IMT *>* 0.9 mm | Hypertension: SBP *>* 140mmHg and DBP *>* 90mmHg on repeated BP measurements |
| Carrelli (2013)  [179] | Inclusion**:**  **PHPT:** patients aged 45–75 years with **mild** hypercalcemia (serum calcium levels < 3.0 mmol/L), referred from the Metabolic Bone Diseases Unit at Columbia University Medical Center between 2005 and 2008.  Exclusion:  Individuals were excluded if they used bisphosphonates within the previous 2 months and if they initiated or changed lipid-lowering therapy within the prior 2 years. From the surgical cohort, 1 participant was excluded due to inability to undergo FMD measurement, and 4 additional participants were excluded from longitudinal analysis (1 lost to follow-up, 1 without post-surgical FMD data, and 2 with persistent PHPT). | Hyperemia diameter (mm):  Before PTX: 3.55 ± 0.6; 6 mo after PTX: 3.57 ± 0.67; 12 mo after PTX: 3.59 ± 0.67 | - Participants fasted 12 h and avoided exercise for 4–6 h before testing.  - High-resolution US (Philips 5500, Philips; 7–15 MHz linear probe): The brachial artery was imaged ~6 cm above the antecubital fossa, and baseline diameter recorded. A forearm cuff was inflated above systolic pressure for 5 min and released to induce reactive hyperemia; arterial diameter was remeasured 1 min after deflation  - FMD was calculated as the percentage change from baseline diameter, with three consecutive assessments averaged. The method demonstrated low intra-observer variability and was consistent with procedures used in large cohort studies. All measurements were performed by a blinded technician | Hypertension: systolic pressure was ≥140 mmHg, diastolic pressure ≥90 mmHg, history of hypertension or use of antihypertensive drugs  Diabetes mellitus: fasting plasma glucose ≥126 mg/dL or a self-reported diagnosis or treatment with insulin or oral glucose-lowering agents |
| Agarwal (2013)  [57] | Inclusion**:**  **PHPT:** consecutive consenting patients who underwent successful PTX between October 2010 and September 2012  **Controls:** healthy, normotensive and normocalcemic individuals  Exclusion:  From the initially eligible PHPT patients, one patient with a structural cardiac defect, three with incomplete follow-up, and one with persistent PHPT after mediastinal adenoma excision were excluded |  | - High-resolution brachial artery Doppler US (Vivid 7, GE Healthcare; 10-MHz linear transducer): The brachial artery was imaged 3 cm above the antecubital fossa, and baseline diameter was recorded. Reactive hyperemia was induced by inflation of forearm cuff 50 mmHg above systolic pressure for 5 min followed by deflation, with diameter measured 30 s after release. FMD was calculated as the percentage change from baseline diameter. Impaired FMD was defined as values <4.5%. NMD was assessed at the same arterial segment 30 s after sublingual administration of 5 mg isosorbide dinitrate (given 15 minutes after deflation of the blood pressure cuff), with post-drug diameter compared with baseline  - Examinations were performed by an experienced cardiologist | Hypertension: history of hypertension or use of antihypertensive drugs |
| Asik (2014)  [44] | Inclusion**:**  **PHPT:** newly diagnosed patients with persistently elevated serum calcium and inappropriately elevated intact PTH levels  **Controls:** age-, sex-, and BMI-matched subjects  Exclusion:  Individuals were excluded if they had a history of CV disease, impaired renal function (creatinine >1.2 mg/dL or creatinine clearance <50 mL/min/1.73 m²), or any systemic illness other than diabetes mellitus or hypertension. Patients with diabetes mellitus were excluded from the control group |  | High-resolution carotid US (Vivid 7, GE Healthcare; linear-array probe): mean IMT of the **right** **common** carotid artery was assessed with the probe aligned longitudinally. A 10- mm segment proximal to the carotid bifurcation was imaged, and **far-wall** IMT was measured as the distance between the lumen–intima and media–adventitia interfaces. Measurements were obtained at three adjacent points, 1-mm apart and averaged  - All scans were performed by a single blinded investigator |  |
| Stamatelopoulos (2014)  [38] | Inclusion**:**  **PHPT:** postmenopausal women with biochemically confirmed PHPT (elevated albumin-corrected serum calcium with concomitantly elevated or inappropriately normal intact PTH levels) and positive Tc99m-sestamibi parathyroid scan  **Controls:** consecutively recruited subjects matched 1:1 by age and duration of menopause, with normal PTH, calcium, and phosphorus levels and no clinically overt CV disease  Exclusion:  Individuals were excluded if menopause duration was < 1 year, or if they had CV disease (prior stroke, coronary artery disease, or symptomatic peripheral or carotid artery disease), reduced renal function (GFR ≤60 mL/min/1.73 m²), acute or chronic inflammatory disorders, or malignancy | Common femoral IMT (mm):  PHPT: 0.65 ± 0.15; Controls: 0.7 ± 0.22  Femoral plaque prevalence (%):  PHPT: 19.8; Controls: 18  Augmentation Index (%):  PHPT: 29.69 ± 7.38; Controls: 29.22 ± 7.34  Aortic PWV (m/s):  PHPT: 9.05 ± 1.7; Controls: 8.58 ± 1.55  Aortic SBP (mmHg):  PHPT: 121.2 ± 21.9; Controls*: 112 ± 15.2  Aortic DBP (mmHg):  PHPT: 79.6 ± 12.4; Controls*: 73.15 ± 9.5 | - All participants abstained from food, smoking, and alcohol for at least 12 hours before the assessment  - High-resolution vascular US (Vivid 7 Pro, GE Healthcare; 14-MHz linear probe): the **right** brachial artery was imaged longitudinally at the antecubital fossa after 10 min supine rest with ECG gating. A forearm cuff was inflated to 250 mmHg for 5 min and then deflated to induce reactive hyperemia. Arterial diameter was measured at 30, 60, and 90 s after deflation, and FMD was expressed as the maximal percent increase from baseline diameter  - IMT of the **common** carotid and femoral arteries and the presence of atherosclerotic plaques were assessed using the same US system. 3 measurements of the maximal IMT of the **far wall** were averaged to obtain mean IMT  - Plaques were defined as focal protrusions into the arterial lumen measuring at least 0.5 mm or ≥50% greater than the adjacent IMT, or having a thickness of ≥1.5 mm.  - Arterial stiffness was determined by carotid–femoral pulse wave velocity (Complior, Artech Medical) and radial applanation tonometry to derive central aortic waveforms and augmentation index (SphygmoCor, AtCor Medical). Peripheral blood pressure was measured at rest and used to calibrate central hemodynamic parameters. | Hypertension: systolic blood pressure ≥140 mmHg and/or diastolic blood pressure ≥90 mmHg based on repeated office measurements, or current use of antihypertensive medication. |
| Walker (2014)  [35] | Inclusion**:**  **PHPT:** patients, both symptomatic and asymptomatic, aged ≥40 years with hypercalcemia (serum calcium ≥10.2 mg/dL), elevated or inappropriately normal PTH levels and available 25-hydroxyvitamin D level, recruited between June 2011 and January 2013.  Exclusion:  Individuals were excluded if they had familial hypocalciuric hypercalcemia, thiazide-induced hyperparathyroidism, current cinacalcet use, malignancy within the previous 5 years (except non-melanoma skin cancer), granulomatous diseases, HIV infection, serum creatinine ≥1.8 mg/dL, liver disease, gastrointestinal disorders affecting calcium metabolism, pregnancy, or congenital heart disease | Maximal carotid plaque thickness (mm):  PHPT with 25-OH-D < 50 nmol/L: 2.18 ± 0.41; PHPT with 25-OH-D ≥ 50 nmol/L: 2.17 ± 0.8  Carotid strain (%):  PHPT with 25-OH-D < 50 nmol/L: 9.6 ± 3.5; PHPT with 25-OH-D ≥ 50 nmol/L: 8.5 ± 3.1  Carotid stiffness*:  PHPT with 25-OH-D < 50 nmol/L: 6.4 ± 2.9; PHPT with 25-OH-D ≥ 50 nmol/L: 7.2 ± 3.8 | High-resolution carotid B-mode US (GE Healthcare LogIQ 700; 9/13-MHz transducer): both extracranial carotid arteries were examined in supine subjects using standardized axial and longitudinal views. Resting brachial blood pressure was measured before and after imaging and averaged. IMT was assessed **bilaterally** as a composite of **near and far** walls of the **common** carotid artery, **bifurcation**, and **internal** carotid artery. Carotid plaque was defined as focal wall thickening >50% greater than adjacent wall, and maximal plaque thickness was determined from multi-angle images using semiautomated software. Carotid mechanics were evaluated in the right common carotid artery by measuring systolic and diastolic diameters over consecutive cardiac cycles to derive carotid strain and stiffness |  |
| Tuna (2015)  [45] | Inclusion**:**  **PHPT:** patients with **mild PHPT** evaluated at Ankara Numune Training and Research Hospital between May 2012 and June 2013. Patients who met one or more criteria from the 2009 National Institutes of Health Consensus Third International Workshop underwent PTX  **Controls:** healthy subjects  Exclusion:  Individuals were excluded if they had diabetes mellitus, coronary heart disease, renal impairment, thyroid dysfunction (hyper- or hypothyroidism), or a history of smoking. Fifteen PHPT patients were excluded from the longitudinal analysis (12 subjects lost during follow-up and 3 who did not have FMD and IMT measurements after PTX) | Brachial artery end-diastolic diameter (mm):  PHPT: 0.34 ± 0.44; Control: 0.33 ± 0.27 | - Measurements performed in fasting state, after at least 10 min of rest, in a quiet, temperature-controlled room  - High-resolution B-mode Doppler US: mean IMT of the **common** carotid artery was obtained from three measurements at different sites and averaged. Endothelial function was assessed by **right** brachial artery FMD using a standardized protocol, with baseline end-diastolic diameters recorded, followed by forearm cuff–induced ischemia (20 – 50 mmHg above systolic arterial pressure for 3 min) and diameter measurement 1 min after cuff deflation. FMD was expressed as the percent change from baseline diameter  - All measurements were performed by the same experienced technician with documented intra-observer reproducibility. | Hypertension: systolic blood pressure ≥140 mmHg, diastolic blood pressure ≥90 mmHg, or a self-reported history of hypertension or use of antihypertensive medication. |
| Yilmaz (2015)  [36] | Inclusion**:**  **PHPT:** patients with asymptomatic PHPT, defined by hypercalcemia with inappropriately normal or elevated plasma intact PTH levels in the absence of classical manifestations such as osteitis fibrosa cystica, fractures, or renal stones  **Controls:** sex-, age- and CV risk factors matched subjects  Exclusion:  Individuals were excluded if they had familial benign hypocalciuric hypercalcemia, a history of coronary artery disease (prior myocardial infarction, angina, angioplasty, or coronary artery bypass surgery), peripheral arterial disease, or diabetes mellitus |  | - Measurements performed between 08.00 - 12.00 after 12-hours of fasting, in a temperature controlled (20–25 °C), quiet room. Caffeine and smoking were prohibited for at least 4–6 hours before the examination, and participants rested in the supine position for at least 10 minutes before assessment.  - High-resolution B-mode Doppler US (General Electric; 5–12 MHz linear-array transducer): endothelial function was assessed by **right** brachial artery FMD. The artery was imaged longitudinally 5 cm above the antecubital fossa; baseline end-diastolic diameter was recorded, followed by upper-arm cuff inflation above systolic pressure for 5 min. Arterial diameter was measured 1 min after cuff deflation, and FMD was expressed as the percent change from baseline diameter.  - Carotid IMT was measured **bilaterally** from longitudinal images of the **common** carotid arteries at a segment ~2 cm proximal to the bifurcation. IMT was defined as the distance between the lumen–intima and media–adventitia interfaces, and the final value represented the average of three consecutive measurements. | Hypercholesterolemia: current or previous use of lipid-lowering medication |
| Cansu  (2016)  [40] | Inclusion:  **PHPT:** newly diagnosed **asymptomatic PHPT patients** (elevated albumin-corrected serum calcium with elevated or inappropriately normal intact PTH levels, in the absence of radiologically confirmed bone lesions or kidney stones)  Patients with PHPT were categorized according to adjusted serum calcium concentrations into a **hypercalcemic group,** with values exceeding the reference range**, and a normocalcemic group**. In normocalcemic PHPT patients, secondary causes of hyperparathyroidism (renal failure, vitamin D deficiency, thiazide or lithium use, renal hypercalciuria, and gastrointestinal disorders associated with calcium malabsorption) were excluded  **Controls:** age- and BMI- matched healthy subjects  Exclusion:  Individuals were excluded if they had MEN type 1 or 2A, familial hypocalciuric hypercalcemia, diabetes mellitus, coronary, cerebrovascular disease, peripheral artery disease, renal impairment (GFR <60 mL/min/1.73 m²), malignancy, inflammatory disorders, smoking history or were using medications that affect PTH or calcium metabolism (estrogens, glucocorticoids, anticonvulsants, loop/ thiazide diuretics, lithium, bisphosphonates). | Augmentation Index aorta (%):  1) Hypercalcemic PHPT: 26 ± 15; Normocalcemic PHPT: 29 ± 12; Controls: 26 ± 7  2) Before PTX: 29 ± 15; After PTX: 24 ± 10  Aortic PWV (m/s)*/**:  1) Hypercalcemic PHPT: 9.6 ± 1.8; Normocalcemic PHPT: 9.3 ± 1.2; Controls: 7.8 ± 0.8  2) Before PTX: 9.6 ± 0.8; After PTX: 8.4 ± 1.5  Aortic SBP (mmHg):  1) Hypercalcemic PHPT: 125 ± 17; Normocalcemic PHPT*: 129 ± 19; Controls: 113 ± 15  2) Before PTX: 125 ± 17; After PTX: 119 ± 15 | - High-resolution carotid US (Mylab 50, Esaote; 5–13 MHz linear probe): **common** carotid quality IMT was measured in supine subjects using radiofrequency-based automated analysis software. A standardized 1.5 cm segment 1 cm proximal to the carotid bifurcation was imaged longitudinally, and **far-wall** quality IMT was automatically derived at systole, averaged over multiple points and cardiac cycles  - All scans were performed by the same experienced, blinded operator.  - Arterial stiffness assessment (TensioClinic Arteriograph, TensioMed): noninvasive measurements provided aortic pulse wave velocity, aortic augmentation index, central systolic blood pressure, and related hemodynamic parameters. Blood pressure and pulse wave recordings were obtained under controlled conditions, and the operator was blinded to group allocation. | Hypertension: systolic blood pressure ≥140 mmHg, diastolic blood pressure ≥90 mmHg or use of antihypertensive medication.  Hyperlipidemia: triglycerides ≥ 1.69 mmol/l, total cholesterol ≥ 5.18 mmol/l after a 12-h fast or use of any lipid lowering treatments |
| Karakose (2016)  [51] | Inclusion:  **PHPT:** newly diagnosed consecutive, symptomatic PHPT patients (elevated serum calcium levels with inappropriately high serum PTH concentrations)  Exclusion:  Individuals were excluded if they had CV disease, diabetes mellitus, Paget’s disease, multiple endocrine neoplasia, renal impairment (GFR <90 mL/min/1.73 m²), secondary hyperparathyroidism, familial hypocalciuric hypercalcemia, endogenous or iatrogenic hypercortisolism, thyroid dysfunction, malignancy, alcohol abuse, or were receiving medications known to alter serum calcium levels. |  | - High-resolution B-mode US (EUB 7000 HV, Hitachi; 12-MHz linear probe): IMT was measured **bilaterally** from three segments of the **common** carotid artery located ~1 cm proximal to the carotid bulb using a standardized lateral imaging angle. Mean IMT was calculated as the average of these measurements  - All scans were performed by the same operator |  |
| Yorulmaz (2017)  [47] | Inclusion**:**  **PHPT:** patients with elevated serum calcium and PTH levels, hypophosphatemia, increased urinary calcium excretion, reduced tubular phosphate reabsorption and parathyroid adenomas confirmed by both parathyroid US and technetium-99m scanning  **Controls:** age- and sex-matched subjects with normal values of biochemical parameters  Exclusion:  Individuals were excluded if they had diabetes mellitus, hypertension, or established CV disease. |  | - Measurements performed after withholding vasoactive medications for at least four half-lives and following a 12-hour overnight fast. Participants were instructed to avoid exercise, caffeine, vitamin C, and tobacco for at least 6 hours before the assessment.  - High-resolution duplex Doppler US (Toshiba Applio; 7.5-MHz linear probe): **Right** brachial artery FMD and NMD were assessed according to standardized guidelines. Baseline arterial diameter was recorded, followed by forearm cuff inflation to 50 mmHg above systolic pressure for 4 minutes to induce ischemia; diameter was measured ~60 s after cuff release for FMD calculation. After a resting interval of 10 min, 0.4 mg sublingual nitroglycerin was administered, and arterial diameter was measured 5 min later to determine NMD. Vasodilatory responses were expressed as percent change from baseline diameter.  - Carotid IMT was measured **bilaterally** from multiple longitudinal projections of the **common** carotid arteries, with three measurements obtained 2 cm proximal to the bulb and averaged to derive the representative carotid IMT value for each subject |  |
| Colak (2017)  [42] | Inclusion**:**  **PHPT:** consecutively recruited patients, divided into 2 groups according to management strategy:   1. Medical 2. Surgical   A third group included patients already operated on (Post-PTX)  Patients evaluated in the preoperative period were not reevaluated prospectively after PTX.  **Controls:** normocalcemic subjects  Exclusion:  Individuals with uncontrolled diabetes mellitus, hyperlipidemia, substance or alcohol dependence, or psychiatric disorders were excluded |  | Studies were conducted in a quiet, temperature-controlled room after fasting and abstinence from smoking or exercise  - High-resolution US assessment (GE Healthcare system; 12L linear probe): the brachial artery was imaged above the antecubital fossa, and baseline diameter recorded. A forearm cuff was inflated above systolic pressure for 5 min and released to induce reactive hyperemia; arterial diameters were measured 1, 2, and 3 min after deflation, with the highest value used to calculate FMD as the percent change from baseline diameter.  Mean carotid IMT was assessed with the same system by the same experienced operator. Both extracranial carotid arteries were imaged in axial and longitudinal views, and carotid IMT was derived as a composite of **near and far wall** measurements of the **common** carotid artery. |  |
| Sambul (2018)  [178] | Inclusion:  **PHPT**: patients were classified in two groups according to management strategy and phenotype: asymptomatic PHPT managed medically without surgical indication; and symptomatic PHPT or asymptomatic PHPT who met criteria for surgery.  **Controls**: healthy subjects  Exclusion:  Individuals were excluded if they were pregnant or smokers or if they had alcohol dependence, cerebrovascular, coronary, or peripheral vascular disease, heart failure, significant valvular heart disease, abdominal aortic aneurysm or dissection, inflammatory or hematologic disorders, cancer, renal failure, obesity, hypertension, diabetes mellitus or hyperlipidemia | Aortic IMT (mm)*:  Medically treated PHPT: 1.4 ± 0.3; Surgically treated PHPT: 1.65 ± 0.26; Controls: 1.24 ± 0.18 | - High-resolution B-mode ultrasonography (EPIQ 7, Philips; 12-MHz linear and 5-MHz convex probes): the abdominal aorta and **bilateral** **common** and **internal** carotid arteries were examined in longitudinal and transverse planes. IMT was defined as the distance between the anterior margins of the first (intima-lumen interface) and the second (collagen-containing top layer of adventitia) echogenic lines and was assessed on the **far wall** from frozen high-quality images. Measurements were obtained from standardized segments of the common carotid artery 1-2 cm proximal to the bifurcation, the internal carotid artery distal to the bifurcation, and the abdominal aorta between the renal and iliac bifurcations  - Multiple measurements were averaged, and analyses were performed by two independent blinded observers. |  |
| Kizilgul (2018)  [32] | Inclusion:  **PHPT:** patients diagnosed between 2012 and 2016 based on persistent hypercalcemia with normal or non-suppressed PTH concentrations  Exclusion:  Individuals were excluded if they had multiple endocrine neoplasia syndromes, parathyroid or thyroid malignancy, hyperparathyroidism–jaw tumor syndrome, or were receiving medications that interfere with calcium or vitamin D metabolism |  | - High-resolution B-mode US (EUB 7000 HV, Hitachi; 13-MHz linear array transducer): carotid IMT was defined as the distance between the lumen–intima and media–adventitia interfaces on B-mode imaging  - All US assessments were performed by a single experienced operator |  |
| Kocabaș (2021)  [48] | Inclusion**:**  **PHPT:** patients with hypercalcemia and high or inappropriately normal PTH levels  **Controls:** healthy volunteers admitted for routine medical assessment  Exclusion:  Individuals were excluded if they had clinical CV disease, major systemic illness (hepatic or renal failure, malignancy, autoimmune or infectious disease), diabetes mellitus, recent trauma or surgery, pregnancy, or were younger than 18 or older than 65 years |  | - High-resolution B-mode US (iE33 xMATRIX system, Philips; 2.5–3.5 MHz transducer): Carotid IMT was assessed by an experienced, blinded cardiologist following guideline-based protocols. With subjects supine and the neck slightly extended and rotated, measurements were obtained bilaterally from the **common** carotid artery, carotid **bulb**, and **internal** carotid artery over standardized 1-cm segments. Mean IMT was defined as the average of all repeated measurements from these sites across both carotid arteries |  |
| Elbuken (2022)  [39] | Inclusion:  **PHPT:** patients with normocalcemic hyperparathyroidism (normal albumin-corrected and ionized calcium levels on two measurements at least 3 months apart with elevated PTH levels), GFR >60 mL/min/1.73 m², 25-OH vitamin D >30 ng/mL, and absence of renal hypercalciuria, malignancy, or other conditions affecting calcium or PTH metabolism.  **Controls:** age- and sex-matched controls with normal albumin-corrected calcium and PTH levels  Exclusion:  Individuals were excluded if they had diabetes mellitus, hypertension, hyperlipidemia, or were using medications for these conditions, as well as if they had malignancy, renal hypercalciuria, impaired renal function, vitamin D deficiency, or any disorder affecting calcium or parathyroid hormone metabolism | Carotid artery end-diastolic diameter (mm):  PHPT: 5.4 ± 0.77; Controls: 5.5 ± 0.8  Maximum carotid IMT (mm)*:  PHPT: 0.8 ± 0.1; Controls: 0.75 ± 0.11 | - High-resolution B-mode US (ACUSON 2000, Siemens; 7.5-MHz probe): **bilateral** **common** carotid arteries were imaged longitudinally at end-diastole by a blinded operator. IMT was measured on the **far wall** as the distance between the lumen–intima and media–adventitia interfaces, and lumen diameter was recorded as the distance between luminal borders. Three measurements were obtained from each side; mean IMT represented the average of these values, and the maximum carotid IMT corresponded to the highest recorded IMT  - IMT >1.3 mm in any segment was considered plaque |  |
| Carnevale (2024)  [37] | Inclusion:  **PHPT:** postmenopausal women evaluated between April 2015 and April 2018. Patients were stratified into surgically and conservatively managed. Re-evaluation after ≥24 months was performed in all patients, except for five women who met surgical criteria but declined intervention and 12 patients who were lost to follow-up  Exclusion:  Individuals were excluded if they were male or premenopausal women |  | - High-resolution B-mode US (MyLab 30, Esaote; 7.5-MHz linear probe): **Far-wall** mean IMT of both **common** carotid arteries was measured in plaque-free segments ≥5 mm from the bulb, averaging three measurements over a 10 mm standardized segment; values from **both** sides were combined for analysis. Plaque was defined as focal wall thickening >1.5 mm and recorded as present or absent  - All measurements performed by a single blinded operator with an intra-observer intra-session coefficient of variability of 2.6% |  |
| Çakıl (2024)  [62] | Inclusion:  **PHPT:** patients aged ≥ 18 years  Exclusion:  Individuals were excluded if they had alternative causes of hypercalcemia (e.g., malignancy-related), secondary or tertiary hyperparathyroidism |  | - High-resolution B-mode US: IMT of **both common** carotid arteries was measured by a single experienced operator with subjects supine. Measurements were obtained from segments approximately 1 cm proximal to the carotid bifurcation, with three readings on each side averaged to derive mean IMT for analysis |  |
| Naciu (2025)  [60] | Inclusion:  **PHPT**: patients with elevated or unsuppressed PTH concentrations and persistently elevated total, albumin-corrected, or ionized serum calcium levels on more than two occasions. All participants in the study had previously received oral cholecalciferol to ensure sufficient 25-hydroxyvitamin D levels  **Controls:** subjects with normal calcium, phosphate, and PTH levels consecutively recruited among individuals referred for unrelated conditions (e.g., euthyroid thyroid nodules) and matched to patients using age- and BMI-based nearest-neighbor matching with the Euclidean distance metric. Matching was performed without replacement to maintain a 1:1 ratio, and balance in age and BMI was verified after matching  Exclusion:  Individuals were excluded if they had disorders influencing bone or calcium metabolism (e.g., familial hypocalciuric hypercalcemia, vitamin D deficiency in controls, malabsorption, thyrotoxicosis, chronic liver or bowel disease, Cushing history, depression, alcoholism, smoking, diabetes, obesity, eating disorders, rheumatologic, hematologic, inflammatory, or autoimmune diseases), were using medications affecting calcium or bone metabolism (thiazides, lithium, bisphosphonates, recent glucocorticoids, or similar therapies), had a history or high risk of CV disease or active malignancy, except low-risk well-differentiated thyroid cancer. | Brachial artery end-diastolic diameter (mm):  PHPT: 3.3 ± 0.29; Controls: 3.1 ± 0.44  Hyperemia diameter (mm):  PHPT: 4.6 ± 0.8; Controls: 4.4 ± 0.8 | - Participants abstained from alcohol, caffeine, smoking, and food for at least 6 hours before the study. Vasoactive medications (nitrates, ACE inhibitors/ARBs, and calcium channel blockers) were discontinued for at least 48 hours prior to the assessment. Examinations performed in a quiet, temperature-controlled room after 10–15 minutes of supine rest.  - High-resolution vascular US (10-MHz linear transducer): Brachial artery FMD was measured from a longitudinal segment above the antecubital fossa at baseline and 1 min after the 5-min cuff inflation to a pressure 50 mmHg above systolic levels and expressed as the percent change in diameter averaged over five consecutive cardiac cycles. Carotid IMT was evaluated **bilaterally** using B-mode imaging of plaque-free **far-wall** segments of the **common** carotid arteries proximal to the bifurcation, defined as the distance between the lumen–intima and media–adventitia interfaces, with averaged values from both sides used for calculation of mean IMT |  |

*Variables for which p < 0.05 in PHPT vs controls

** Variables for which p < 0.05 in PHPT before vs after parathyroidectomy

**Supplementary Table S2.** **Results of meta-regression analysis for common carotid artery IMT in studies comparing PHPT patients with controls**. CI, confidence interval; β, regression coefficient; BMI, body mass index; SBP, systolic blood pressure; DBP, diastolic blood pressure.

| **Moderator** | **β** | **95% CI** | **p** |
| --- | --- | --- | --- |
| PTH | 0.004 | - 0.002 − 0.009 | 0.174 |
| Age | -0.007 | -0.014 – 1.74 * 10^-4^ | 0.055 |
| % Females | 2.33 * 10^-4^ | -0.005 – 0.005 | 0.922 |
| Total calcium | 0.072 | -0.182 – 0.327 | 0.548 |
| BMI | 7.138 * 10^-4^ | -0.027 – 0.029 | 0.955 |
| SBP | - 4.681 * 10^-4^ | -0.01 – 0.010 | 0.918 |
| DBP | 0.007 | -0.023 – 0.037 | 0.604 |
| Total cholesterol | -0.136 | -0.430 – 0.158 | 0.301 |

**Supplementary Table S3. Results of meta-regression analysis for FMD in studies comparing PHPT patients with controls**. CI, confidence interval; β, regression coefficient; BMI, body mass index; SBP, systolic blood pressure; DBP, diastolic blood pressure.

| **Moderator** | **β** | **95% CI** | **p** |
| --- | --- | --- | --- |
| PTH | -0.164 | -0.497 − 0.169 | 0.288 |
| Age | 0.693 | 0.107 – 1.280 | 0.026 |
| % Females | 0.130 | -0.099 – 0.359 | 0.221 |
| Total calcium | -8.768 | -38.49 – 20.95 | 0.516 |
| BMI | -0.151 | -3.132 – 2.831 | 0.906 |
| SBP | 0.280 | -1.462 – 2.022 | 0.678 |
| DBP | 1.672 | -1.640 – 4.984 | 0.234 |
| Total cholesterol | 8.826 | -14.10 – 31.76 | 0.308 |
